# Supplementary material for: Demographic and health community-based surveys to inform a malaria elimination project in Magude district, southern Mozambique
Source: BMJ Open. 2020 May 5;10(5):e033985. doi: 10.1136/bmjopen-2019-033985 (PMC7228537; doi:10.1136/bmjopen-2019-033985)

Supplementary Tables

S.Table 1: **Selected demographic and socioeconomic characteristics of Magude's population (2015).**

Variable / by sex	Males		Females	
	N	Col %	N	Col %
**Age groups (years)**	21775		26673	
< 1	826	3.8	898	3.4
1-4	3391	15.6	3475	13.0
5-14	7345	33.8	7262	27.2
15-64	9334	42.9	13205	49.5
>=65	864	4.0	1824	6.8
**Education Level (>= 6 years old)**	16712		21461	
Illiterate	7925	47.4	11136	51.9
5-9 grade	6221	37.2	7616	35.5
10-12 grade	1595	9.5	1825	8.5
University	43	0.3	20	0.1
Missing Information	928	5.6	864	4.0
**Occupation (> 18 years old)**	5243		5532	
Farmer or Fisherman	1371	26.1	3909	70.7
Salesperson	466	8.9	609	11.0
Construction work occupations	1159	22.1	39	0.7
Coal maker / Lumberjack	603	11.5	173	3.1
Guards/Police/Military	539	10.3	45	0.8
Health professional	85	1.6	133	2.4
Teacher	263	5.0	180	3.3
Miner	106	2.0	9	0.2
Other	651	12.4	435	7.9
**Marital Status (> 14 years old)**	10157		14986	
Single (never married)	6168	60.7	7772	51.9
Married or de-facto union	3690	36.3	4702	31.4
In a polygamic relationship	202	2.0	-	-
Divorced	7	0.1	13	0.1
Separated	138	1.4	541	3.6
Widow	154	1.5	1958	13.1

S. Table 2: Modified household poverty index (PI) based on the following deprivation indicators: 1) lack of electricity, 2) lack or sharing of an improved sanitation facility; 3) lack of access to improved drinking water source, or source only available more than 30-minute walk, round trip; 4) dirt, sand or dung household floors; 5) dung, wood or charcoal used for cooking fuel; and 6) households that do not own a car or truck AND do not own more than one of the following: radio, TV, telephone, bike, motorbike, or refrigerator.

Deprivations	Magude	Motaze	Panjane	Mahele	Mapulanguene	District
0-2	32.1	9.8	1	0.9	13	25.2
3-4	49.4	58.3	55.1	61	70.8	52.3
5-6	18.5	31.9	43.9	38.1	16.2	22.4
Median	3	4	4	4	3	4
Mean	3.1	3.9	4.2	4.2	3.5	3.4

S.Table 3: Number of individuals by age group who reported spending one night outside of Magude the day before the census visit was performed (2015).

		Men		Women		All	
Age group	Previous night spent outside of Magude	N	(%)	N	(%)	N	(%)*
0 - 14	Yes	592	5.1	580	5.0	1172	43,0*
	No	10874	94.0	10970	94.3		
	NA±	96	0.8	85	0.7		
15 – 29	Yes	324	6.3	362	5.6	686	25.1*
	No	4774	92.7	6022	93.3		
	NA±	52	1.0	70	1.1		
30 – 44	Yes	298	11.3	197	5.3	495	18.1*
	No	2323	87.8	3497	93.9		
	NA±	24	0.9	30	0.8		
45 – 64	Yes	133	8.6	149	4.9	282	10.3*
	No	1393	90.5	2839	93.8		
	NA±	13	0.8	39	1.3		
> 65	Yes	36	4.2	57	3.1	93	3.4*
	No	818	94.7	1744	95.6		
	NA±	10	1.2	23	1.3		
All ages	Yes	1383	6.4	1345	5.0	2728	5.6
	No	20182	92.7	25072	94,0	45254	93.5
	NA±	195	0.9	247	1.0	442	0.9

* Percentage of those who reported travelling (2,728)

± Missing Information

S.Table 4: **Household-level health and malaria prevention indicators in Magude district (2015).**

Administrative Post	Magude-Sede		Motaze		Panjane		Mahele		Mapulanguene	
Household size	4 [3-6]		4 [3-7]		4 [2-6]		4 [2-6]		3 [2-5.5]	
Members per net Median [IQR]	2.0 [1.4-4.0]		2.0 [1.3-3.0]		2.0 [1.6-5.5]		2.0 [1.5-4.0]		2.0 [1.3-4.0]	
Universal ITN coverage *	52.7%		59.9%		44.1%		52.7%		52.1%	
IRS in past 12 months	N	%	N	%	N	%	N	%	N	%
Yes	3925	49.0	1162	79.0	258	41.1	230	61.0	147	30.7
No	3627	45.3	293	19.9	352	56.1	133	35.3	314	65.6
Unknown	459	5.7	16	1.1	17	2.7	14	3.7	18	3.8

** One net for every 2 members of the households

Supplementary Figures

S.Figure 1: Household Assets in Magude district in 2015.

Percentage of households in Magude district with at least one item per asset, and median and interquartile range of the assets for which the number of items available was higher than 1.

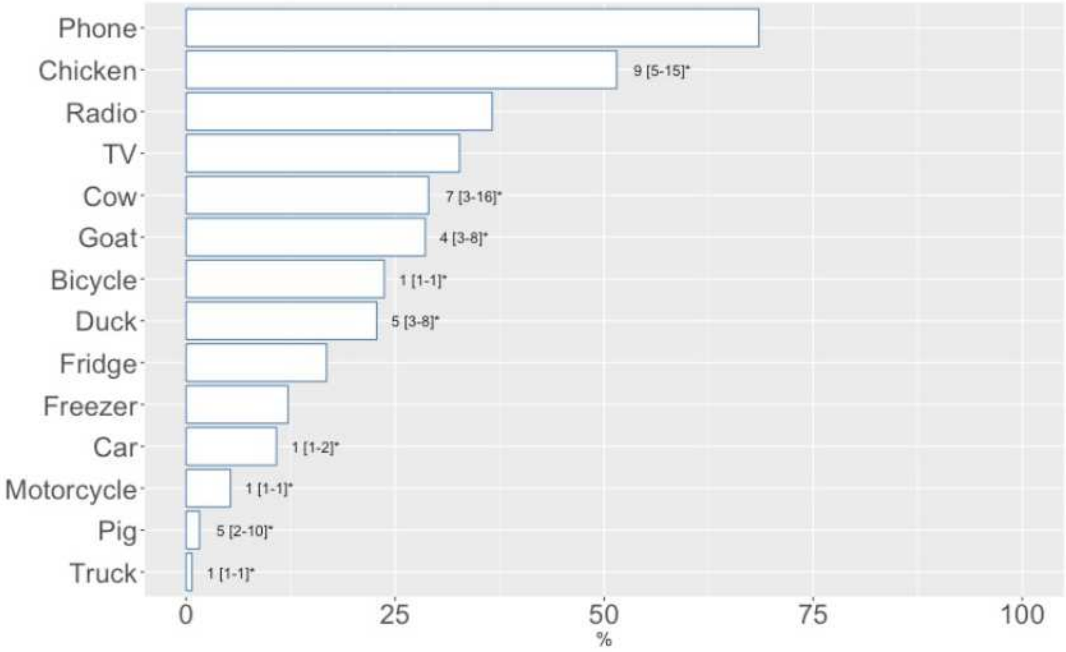

S.Figure 2: Mobility patterns of Magude's population in 2015.

Main destinations per age group among those who reported travelling outside of Magude in 2015.

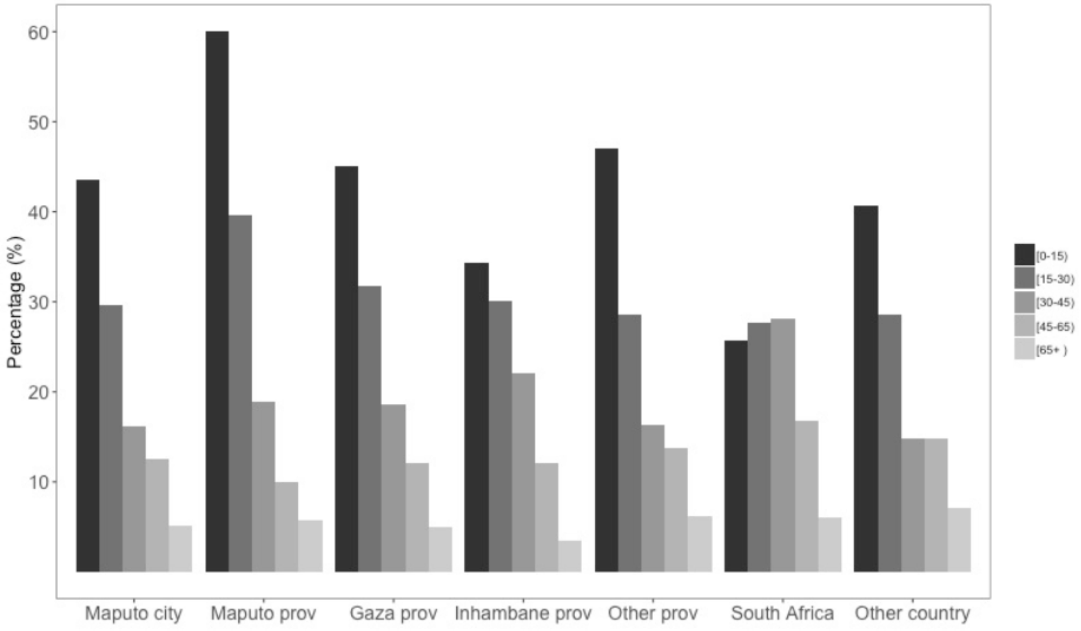

Supplement: Supplementary data [file bmjopen-2019-033985supp001.pdf]
