# Supplementary material for: Demographic and health community-based surveys to inform a malaria elimination project in Magude district, southern Mozambique
Source: BMJ Open. 2020 May 5;10(5):e033985. doi: 10.1136/bmjopen-2019-033985 (PMC7228537; doi:10.1136/bmjopen-2019-033985)
Supplement: Author's manuscript [file bmjopen-2019-033985.draft_revisions.pdf]

BMJ Open is committed to open peer review. As part of this commitment we make the peer review history of every article we publish publicly available.

When an article is published we post the peer reviewers' comments and the authors' responses online. We also post the versions of the paper that were used during peer review. These are the versions that the peer review comments apply to.

The versions of the paper that follow are the versions that were submitted during the peer review process. They are not the versions of record or the final published versions. They should not be cited or distributed as the published version of this manuscript.

BMJ Open is an open access journal and the full, final, typeset and author-corrected version of record of the manuscript is available on our site with no access controls, subscription charges or pay-per-view fees (http://bmjopen.bmj.com).

If you have any questions on BMJ Open's open peer review process please email info.bmjopen@bmj.com

BMJ Open

Demographic and health characteristics of Magude district, Southern Mozambique

Journal:	*BMJ Open*
Manuscript ID	bmjopen-2019-033985
Article Type:	Original research
Date Submitted by the Author:	04-Sep-2019

Complete List of Authors:	Galatas, Beatriz; Instituto de Salud Global Barcelona, Malaria Elimination Initiative; Centro de Investigacao em Saude de Manhica, ISGLobal<br>Nhacolo, Ariel; Centro de Investigacao em Saude de Manhica<br>Munguambe, Humberto; Centro de Investigacao em Saude de Manhica<br>Marti, Helena; Instituto de Salud Global Barcelona, Malaria Elimination Initiative<br>Jamise, Edgar; Centro de Investigaçao em Saúde de Manhiça<br>Guinovart, Caterina; Barcelona Institute for Global Health<br>Cirera, Laia; Instituto de Salud Global Barcelona, Malaria Elimination Initiative<br>Amone, Felimone; Centro de Investigacao em Saude de Manhica<br>Macete, Eusebio; Centro de Investigação em Saúde de Manhiça<br>Bassat, Quique; BARCELONA INSTITUTE FOR GLOBAL HEALTH<br>Rabinovich, Regina; Instituto de Salud Global Barcelona, Malaria Elimination Initiative<br>Alonso, Pedro; WHO, GMP<br>Aide, Pedro; Manhiça Health Research Centre<br>Saute, Francisco; Centro de Investigação em Saúde de Manhiça<br>Sacoor, Charfudin; Centro de Investigacao em Saude de Manhica, Department of Demography

Keywords:	Public health < INFECTIOUS DISEASES, Demography < TROPICAL MEDICINE, Magude project, Malaria elimination, Population and health

SCHOLARONE™
Manuscripts

I, the Submitting Author has the right to grant and does grant on behalf of all authors of the Work (as defined in the below author licence), an exclusive licence and/or a non-exclusive licence for contributions from authors who are: i) UK Crown employees; ii) where BMJ has agreed a CC-BY licence shall apply, and/or iii) in accordance with the terms applicable for US Federal Government officers or employees acting as part of their official duties; on a worldwide, perpetual, irrevocable, royalty-free basis to BMJ Publishing Group Ltd ("BMJ") its licensees and where the relevant Journal is co-owned by BMJ to the co-owners of the Journal, to publish the Work in this journal and any other BMJ products and to exploit all rights, as set out in our licence.

The Submitting Author accepts and understands that any supply made under these terms is made by BMJ to the Submitting Author unless you are acting as an employee on behalf of your employer or a postgraduate student of an affiliated institution which is paying any applicable article publishing charge ("APC") for Open Access articles. Where the Submitting Author wishes to make the Work available on an Open Access basis (and intends to pay the relevant APC), the terms of reuse of such Open Access shall be governed by a Creative Commons licence – details of these licences and which Creative Commons licence will apply to this Work are set out in our licence referred to above.

Other than as permitted in any relevant BMJ Author's Self Archiving Policies, I confirm this Work has not been accepted for publication elsewhere, is not being considered for publication elsewhere and does not duplicate material already published. I confirm all authors consent to publication of this Work and authorise the granting of this licence.

For peer review only

Demographic and health characteristics of Magude district, Southern Mozambique

Authors

Beatriz Galatas[1, 2]*¶, Ariel Nhacolo¶[1], Helena Martí-Soler[1,2], Humberto Munguambe[1], Edgar Jamise[1], Caterina Guinovart[2], Laia Cirera[1,2], Felimone Amone[1], Eusébio Macete[1,3], Quique Bassat[1,2,5,6], N. Regina Rabinovich[2,7], Pedro L. Alonso[1,2,], Pedro Aide[1,4], Francisco Saute[1], Charfudin Sacoor[1]

Affiliations

1. Centro de Investigação em Saúde da Manhiça (CISM), Manhiça, Mozambique;
2. Barcelona Institute for Global Health (ISGlobal), Hospital Clínic-Universitat de Barcelona, Barcelona, Spain.
3. National Directorate of Health, Ministry of Health, Mozambique
4. National Institute of Health, Ministry of Health, Maputo, Mozambique
5. ICREA, Pg. Lluís Companys 23, 08010 Barcelona, Spain.
6. Pediatric Infectious Diseases Unit, Pediatrics Department, Hospital Sant Joan de Déu (University of Barcelona), Barcelona, Spain
7. Harvard T.H. Chan School of Public Health, Boston, MA, USA.

*Corresponding Author

E-mail: Beatriz.galatas@isglobal.org

¶ These authors contributed equally to this work

Emails of Authors

Beatriz.galatas@isglobal.org

Ariel.nhacolo@manhica.net

Humberto.munguambe@manhica.net

Helena.marti@isglobal.org

Laia.cirera@isglobal.org

Felimone.amone@manhica.net

Eusebio.macete@manhica.net

quique.bassat@isglobal.org

regina.rabinovich@isglobal.org

Alonsop@who.int

Pedro.aide@manhica.net

Francisco.saute@manhica.net

For peer review only

Charfudin.sacoor@manhica.net

Key words

Magude district, population, health, malaria control tools, malaria elimination

Word count

5082

For peer review only - http://bmjopen.bmj.com/site/about/guidelines.xhtml

Abstract

(Word count: 289)

Objectives: A Demographic and Health Platform (DHP) was established in Magude in 2015, prior to the deployment of the project's interventions. This platform aimed to inform the design, implementation and evaluation of a malaria elimination project planned for the district of Magude, through the identification of households, and population; and the collection of demographic, health and malaria information.

Setting: Magude is a rural district of Southern Mozambique which borders South Africa. It has nine rural health facilities and one referral health center with an inpatient ward.

Intervention: A baseline census geolocated and enumerated all the households, residents and non-residents, collected demographic and socio-economic information at the household and individual levels, as well as information on the coverage and usage of malaria control tools. Inpatient and outpatient data were obtained from the district health authorities. The demographic platform was updated in 2016.

Results: The baseline census conducted in 2015 reported 48,448 (92.1%) residents and 4,133 (7.9%) non-residents, and 10,965 households in Magude. Magude's population is predominantly young, half of the population is illiterate and the main economic activities are agriculture and fishing. Houses are mainly built with traditional non-durable materials and have poor sanitation facilities. Between 2010 and 2014, malaria was the most common cause of inpatient admissions (representing up to 20-40% of all inpatient admissions), followed by HIV (12-22%), and anemia (12-15%). In early 2015, all-age bed-net usage was between 21.8%-27.1% and the reported coverage of indoor residual spraying varied across the district between 30.7% and 79%.

Conclusion: This study revealed that Magude has limited socio-economic conditions, poor access to health care services and low coverage of malaria vector control interventions. Thus, Magude represented an area where it is most pressing to demonstrate the feasibility of malaria elimination strategies.

For peer review only - http://bmjopen.bmj.com/site/about/guidelines.xhtml

Strengths and limitations of this study

- Prior to the implementation of the malaria elimination project named the *Magude project*, the most recent census data available had been collected during the national census that took place in 2007. Thus, a Demographic and Health Platform (DHP) was established in Magude in 2015 to inform and accurately plan the interventions contemplated under the elimination project. The DHP geolocated and enumerated all the households, residents and non-residents of Magude, collected demographic and socio-economic information at the household and individual levels, as well as information on the coverage and usage of malaria control tools.
- A second census round was conducted one year later acknowledging that detailed population data collected at individual level at one point in time may miss individuals or households that can be captured after a later update. As a result, this DHP found 48,448 residents and 10,965 households in Magude in 2015, and 56,943 residents and 11,960 households in 2016. Socio-economic and population mobility patterns were depicted by location, sex or age. In 2015, all-age bed-net usage was between 21.8%-27.1% and the reported coverage of indoor residual spraying (IRS) varied across the district between 30.7% and 79%.
- The census rounds were planned in close collaboration with the community leaders and district authorities, and counted with intensified training of field workers, and a strong component of field and data supervision by experienced demographers. Nevertheless, the data collected through the DHP could have been affected by inaccuracies during data collection or entry, or by recall or desirability bias of census participants.
- To gather baseline data for the Magude project, and understand the health profile of the district, inpatient and outpatient data were obtained from the Weekly epidemiological bulletin (BES) reported from nine active rural health facilities; and monthly inpatient data was obtained from the health center with an inpatient ward. Between 2010 and 2014, malaria was the most common cause of inpatient admissions (representing up to 20-40% of all inpatient admissions), followed by HIV (12-22%), and anemia (12-15%).

For peer review only - http://bmjopen.bmj.com/site/about/guidelines.xhtml

- Inpatient information was limited by the quality and accuracy of the data at the time it was collected, by disease-specific interventions or changes in diagnostics, referral or reporting practices. Cases reported by BES are subject to RDTs stock-outs or reporting inaccuracies, and usually exclude cases identified by community health workers. This supported the plans to establish a stronger surveillance system in Magude to fully capture changes in malaria during the malaria elimination project.

For peer review only

For peer review only - http://bmjopen.bmj.com/site/about/guidelines.xhtml

Background

Mozambique is one of the countries with the highest malaria burden in the world [1]. Malaria prevalence is heterogeneous within the country ranging from high transmission intensity in the North (>50%) to less than 3% in the South [2], which has experienced great progress against malaria in the past decades, partly as a result of the regional initiatives aiming for malaria elimination in the area. In line with the vision of a malaria free world established by the World Health Organization in its Global Technical Strategy for 2016-2030 [3], the National Malaria Control Program of Mozambique (NMCP) decided to redefine its strategic objectives in order to include the implementation of malaria elimination activities in the south. In this context, a malaria elimination project named the *Magude Project* was designed and evaluated in Magude district, Maputo province, by the Manhiça Health Research Center (CISM) and the Barcelona Institute of Global Health (ISGlobal), to assist the NMCP in adopting a malaria elimination strategy based on local evidence [4].

Prior to the initiation of the Magude project in 2015, there was limited and outdated information with regards to the number of individuals living in Magude, as well as to their demographic and socio-economic characteristics [5]. Thus, detailed information from the whole district was deemed crucial to inform the elimination strategies that had been planned for the following years in the district. The process of filling in this knowledge gap also aimed to identify and contact key leaders at provincial, district, and at community level, to inform and engage them in the activities prior to their deployment. In this context, a Demographic and Health Platform adapted from the Health and Demographic Surveillance System (HDSS) method was established in the district of Magude, in February of 2015 with the objective of providing reliable and updated demographic data to inform the project. This platform allowed to plan the activities and to provide a sampling frame to measure indicators in the community such as malaria prevalence, and to estimate the coverage of indoor residual spraying (IRS), coverage and usage of long-lasting insecticide treated nets (LLIN) and mass drug administration (MDA) campaigns. The DHP's permanent identification numbers were used to track individual's participation in each intervention of the Magude project longitudinally, thus allowing to identify and quantify potential challenges to the project, such as reasons

For peer review only - http://bmjopen.bmj.com/site/about/guidelines.xhtml

for non-participation, or probable sources of imported infections. These data was also used to accurately measure prevalence at the community stratified by age groups and areas of residence. Overall, these findings were crucial to the design of the Magude project, and offered robust evidence to guide malaria elimination strategies in southern Mozambique.

This article presents the demographic, socio-economic and health characteristics of the population of Magude, as well as the coverage of malaria control interventions estimated through the baseline census conducted in early 2015, prior to the deployment of interventions. It also provides a summary of the demographic profile of Magude after updating the census in 2016.

Methods

Study area

The district of Magude is located in the North-Western part of Maputo Province and borders with the districts of Massingir, Chókwe and Bilene, from Gaza Province on the North and North-East; with the districts of Manhiça and Moamba, of Maputo Province in the East and South; and with the South African National Kruger Park, in the West (Figure 1A).

Magude was selected as an appropriate demonstration area for the malaria elimination project as it was expected to pose the types of challenges that the NMCP would face when implementing a malaria elimination campaign in the south of the country. First, there were more than 13,000 malaria cases reported in the district in 2014, with the majority of cases observed between January and May, suggesting that the epidemiology of malaria in the district was representative of most endemic areas in the country with the typical seasonal pattern coinciding with the rainy season [6,7]. Second, the socio-economic and infrastructural limitations reported for Magude made it sufficiently representative of a rural district of Mozambique, while still at reach of CISM's facilities (located in Manhiça district), which facilitated logistics, supervision and quality control processes. Finally, the population of Magude had had very limited exposure to research projects or targeted innovative malaria interventions prior to 2015, having only received the programmatic IRS, LLINs and child immunization campaigns

For peer review only - http://bmjopen.bmj.com/site/about/guidelines.xhtml

conducted by the government. This allowed facing the challenges of working in an unexposed population that was not biased by previous activities.

Data collection procedures

A Demographic and Health Platform (DHP) was established in Magude in February 2015, by CISM, which was adapted from the HDSS previously established in Manhiça district by CISM [8]. A baseline census was conducted at that time (February to June) to identify and enumerate all neighborhoods, households and resident and non-resident individuals in the district. GPS coordinates were also captured for every household. Each individual was assigned a permanent and unique identification number, linked to the household number where they were first enumerated. A second census round was conducted between August and September of 2016, to review and update all information collected during the baseline census and record live births, deaths, and migrations. New households and new members were enumerated, and their information was collected to update the baseline databases.

This DHP defined a household as a structure or set of constructions where an individual or group of individuals live and share domestic activities and costs (such as eating and sleeping) and recognize one of them as their superior or chief, regardless of their kinship ties. Individuals were defined as residents if they had lived and slept in a household within the study area for a period of three months or more or intended to do so. Non-resident members of a household were defined as those who left the district or who had never lived in the study area for a period of three or more months, but who kept their roles in the households as head of household, husband, or other important breadwinners, and paid regular visits to their households in Magude. These individuals leave their households for specific reasons such work, studies, or imprisonment; and would otherwise reside in the referred household. This category excluded offspring or other members who had left the household to build their new residencies outside the study area, irrespective of whether they kept visiting the reference household or not.

Baseline and updated data from the census rounds were collected through standardized questionnaires using Open Data Kit (https://opendatakit.org/) installed in Android tablets. Data were sent to a secure server at CISM using Wi-Fi. Information collected on households' socio-economic

For peer review only - http://bmjopen.bmj.com/site/about/guidelines.xhtml

characteristics included building materials used for the main house, source of water and electricity, and household assets and livestock. At the individual level, the information collected included the name, sex, date of birth, relation to the head of household, education, and occupation of all residents and non-residents of Magude. Information was also collected on malaria prevention tools, including the possession and use of LLINs, and whether the house had received IRS in the preceding year. Individuals were also asked about history of fever during the preceding 30 days, and whether they had sought care at a health facility, community health worker, traditional healer, or none. The specific health facility where the individual sought care was also specified to delineate health facility catchment areas (HFCA) according to the community. Finally, information was collected on the migration and mobility patterns of all participants, to better characterize the mobility profile of the district, and offer potential information on the sources of new infections if transmission was eventually significantly reduced.

Administrative and geographical information was obtained directly from the district authorities and from the most recent district profile of Magude published in 2014 [7] and district statistics performed by the National Institute of Statistics [9,10]. Data on inpatient discharges and weekly outpatient malaria cases (Boletim Epidemiológico Semanal or "BES") reported through the national health information system between 2010-2014 were obtained from the District Health authorities.

Patient and Public Involvement

This study counted with the support and involvement of the community of Magude in all of its stages. During its design and planning, meetings were held with the district authorities and community leaders in order to inform them of the purpose of the DHP under the scope of the Magude project, as well as to landscape the number of villages in Magude that had to be covered by the DHP. The study team worked closely with village chiefs when performing the household visits, which ensured that all district households were covered by the DHP. Reports were submitted to the administrative authorities to support vector control activities, among others. Study findings were also communicated in meetings held with the community prior to the deployment of MDAs, where the findings

For peer review only - http://bmjopen.bmj.com/site/about/guidelines.xhtml

obtained from the study - particularly focusing on malaria - where discussed in detail [11].

Data analysis

Data management and descriptive analyses were performed using R Software [12]. No inferential statistics (p-values or 95% confidence intervals) were calculated as the DHP covers the entire district, and therefore offers direct population parameters. Spatial visualization of the data collected as well as the open source administrative data bases from DIVA-GIS [13] were performed using QGIS Software [14].

Results

Geography of Magude district

The district of Magude has an area of 6,961 km^2 and is divided in five administrative posts, namely: Magude-sede, Motaze, Panjane, Mahele and Mapulanguene (Figure 1A). The vegetation of Magude is dominated by open forests and savannahs hosting animals such as impalas, warthogs, lions, buffalos and elephants. There is one permanent river (Incomati), which flows through the south-western region of the district and constitutes the main source of water in the area, and three intermittent rivers dependent on rainfall, called Massitonto, Uanétse and Mazimuchopes [7].

Demographic and socio-economic characteristics

The baseline census of 2015 registered 10,965 households and 52,802 individuals, of whom 48,448 (91.8%) were residents and 4,133 (7.8%) were non-residents. The district had a population density of 6.9 inhabitants per km^2. The census update conducted between August and October of 2016 registered a population of 61,868 individuals. Of the population censed in 2015, 42,792 (81%) were found during the update round, 6,099 (11.5%) were reported to still be in Magude by a family member although field workers did not find them in other households and therefore were not able to confirm and complete the registration of migration. There were an additional 3,244 (6.1%) individuals censed in 2015 who were not found in 2016 and for whom there were no informants, but were still considered to be in Magude for this analysis. This update also recorded the death of 670 individuals censed in 2015, 1,687 live births and 721 immigrations since the baseline census.

For peer review only - http://bmjopen.bmj.com/site/about/guidelines.xhtml

An additional 7,325 individuals who were missed in 2015 were censed during this update (Table 1).

The population in Magude is irregularly distributed among the five administrative posts. The majority of the population (73%) lives in the Magude-Sede administrative post (the capital of the district); 14.1% in Motaze, 5.9% in Panjane, 3.7% in Mapulanguene, and 3.4% in Mahele (Table 1). The age and sex structure of the population is dominated by a young population, and a sharp reduction in the adult population, particularly among males (Figure 1B). There is a steady reduction in the male to female ratio from the age of 15 onwards, with a notable reduction in the proportion of males older than 30.

Sixty-two percent of males and 63.1% of females above the age of 14 reported being married or in de-facto union; and 3% of married men practice polygamy. The illiteracy rate among those aged 6 and older in Magude is 51.9% among females and 47.4% among males. Approximately 37.2% of individuals reached 5th to 9th grade, and 9.5% have completed between 10th and 12th grade (Sup.Table 1). In 2015 Magude had 31 primary schools offering 1st to 5th grades; 33 offering 1st-7th grades; one secondary school offering 8th-12th grades; and one private higher-education training center. Xichangana is the main local language of the district and is the mother tongue of 92% of the population of Magude. However, 51% of the population also reports being able to speak Portuguese, which is the mother tongue of only 3.2% of the population [7].

Occupations are unevenly distributed between males and females older than 18 years of age. The majority of the population (26.1% of males and 70.7% of females) relies on subsistence agriculture, fishing or work as cane cutters in the sugar plantations within Magude, or in Xinavane, in the nearby district of Manhiça. Other occupations include being a salesperson (8.9% of men and 11% of women), doing construction work (22.1% of men, 0.7% of women), or making coal (11.5% of men and 3.1% of women). A small proportion of the population are security guards, or work in the public sector, particularly as health professionals, teachers or in the military (Sup.Table 1).

For peer review only - http://bmjopen.bmj.com/site/about/guidelines.xhtml

Men represent 54.6% of heads of households in Magude. Only 0.4% of household heads are younger than 18 and 5.6% are between the ages of 18-24, while 74.7% are between 25-64 years old, and 19.2% are older than 65 years of age (Table 2). Forty-three percent of heads of household are single, 35.4% are married or in de-facto union, 16.8% are widows and 4.3% separated. The illiteracy rate among heads of households is 59%, while 28.2% have completed up to 5-7th grade, 8.7% have completed between 10th to 12th grade, and 0.4% have a university degree (Table 2).

Living conditions in Magude's households

Settlements in Magude are made of individual household compounds that are built in proximity to each other in the central areas of each administrative post, and more spread out in the rest of the district. The median household size is 5, although 1,171 households (10.7%) have only one resident, 40% of whom are older than 65 years old.

The majority of households are traditional round-shaped or rectangular-shaped huts constructed using cane (32.5%), cement (26%), mud bricks (21.6%), or reeds covered by adobe (15.6%) (Table 2). More than half of the households have traditional latrines (53.5%), 10.6% have improved latrines, while 33.8% of households do not have any form of sanitation facility. Flush toilets can only be found in 2.2% of the households. The primary lighting sources used in the households are paraffin (49.6%), electricity (30.8%), candles (9.9%) and solar panels (4.6%). The majority of households cook using wood logs (59.3%). Water is mainly obtained from pumps (34.3%) or piped water (20.5%), although a quarter of the population collects water directly from the river (24.9%) or from open wells near the river (10.5%) (Table 2).

With regards to household assets, 68.5% of households own at least one mobile phone, 36.6% own a radio, 32.7% have a television (TV), while 16.8% and 12.2% of households have a fridge and a freezer, respectively. Almost a quarter of households reported having bicycles (23.7%), while 10.8% reported owning cars, 5.3% motorbikes, and 0.7% trucks (Sup.Figure 1).

For peer review only - http://bmjopen.bmj.com/site/about/guidelines.xhtml

Mobility patterns

Five percent of residents reported having spent the night before the census outside of Magude (6.4% of males and 5% of females). Of these, 43% were younger than 14 years old, 25.1% were 15-29, 18.1% were 30-44, 10.3% were 45-64 and 3.4% were older than 65 years old (Sup.Table 2).

Almost half of the population (43% of males and 46.9% of females) did not report frequently travelling outside of Magude. Among the 54.4% who did, the places most frequently visited included Maputo City (55.3% of males, 58.3% of females who travel), South Africa (23.1% of males and 16.8% of females), Gaza Province (12.2% of males and 15.9% of females), Inhambane Province (4.3% males and 2.6% females), and other Northern provinces within Mozambique (5.2% of males and 6.3% of females). Younger individuals (less than 15 years old) reported travelling the most to all provinces within the country, followed by those between the ages of 15-30; while 15 to 45-year-olds are more likely to travel to South Africa. Individuals older than 45 reported travelling more to provinces northern of Maputo province, as well as abroad (Sup.Figure 2).

Health and malaria information

In 2015, the district health services had eight health facilities (HF), of which only one was of Type I, and the other 7 were of Type II. This Type I HF is located in the Magude-Sede administrative post and had a maternity and inpatient department, with 57 beds and three active medical doctors (0.06 doctors per 1,000 inhabitants). The other 7 health facilities without an inpatient unit but with maternity wards are of Type II, and offer 29 beds altogether but no medical doctors. The Type II HFs are distributed among all administrative posts of the district: two in Magude Sede, one in Motaze, one in Panjane, two in Mahele and one in Mapulanguene. An additional Type II health facility with 3 beds was introduced in 2016 in Magude Sede and another one with 5 beds in 2017 in Mapulanguene, thus altogether contributing to 9 Type II health facilities with 37 beds and one Type I health facility for the entire district, with three medical doctors, 15 general nurses and 19 maternal and child health nurses (Figure 1A). Of these, only 3 have access to piped water, 4 have access to public network electricity, and 5 rely only on solar panels. All health facilities are located on a main road to facilitate access, and the median Euclidian

For peer review only - http://bmjopen.bmj.com/site/about/guidelines.xhtml

distance from households to the nearest health facility is 2.7 km (interquartile range [IQR] 1.4–7.9 km), although households are as close as 15 m or as far as 38.8 km. There is only one ambulance in Magude, which is used for transferring patients within the district, but also to the hospitals in Xinavane, Manhiça, and Maputo.

There are 27 Community Health Workers (or APEs, from its acronym in Portuguese) in Magude distributed throughout the district (Figure 1A). The average distance between households and the assigned APE is approximately 6.3 km (median of 4.4 km, IQR 2.8–5.4 km). APEs are trained by the Ministry of Health to offer primary health services in areas with poor access to HFs. They provide diagnosis and treatment for malaria, diarrhea, and pneumonia, and refer patients with signs of sickness requiring higher medical attention [15].

All HFs and APEs in Magude are equipped to diagnose malaria through Rapid Diagnostic Tests (RDTs) –light microscopy is only available in the Magude Sede Type I HF. They also offer treatment to all positive cases with Artemether-Lumefantrine, the first line treatment in Mozambique. Intermittent Preventive Treatment in pregnancy (IPTp) using Sulfadoxine-pyrimethamine is also offered in all HFs of the district. In May of 2014 the NMCP conducted an LLIN universal distribution campaign, with 35,432 bed nets distributed in Magude district. This was followed by a focal IRS campaign between October and December of 2014 using the insecticides deltamethrine and DDT, which was only deployed in the Motaze administrative post, where the malaria case-load was highest.

Malaria has traditionally been the first cause of disease in the district, responsible for approximately 53% of all consultations reported in 2003 [7]. According to the inpatient discharge data available from January of 2011 to December of 2014, between 20-40% of all-age discharges in the inpatient department were due to malaria, followed by HIV (12-22%), anemia (12-15%), pneumonia (6-12%), and diarrhea (3-8%), varying by month and year (Fig 2A). The weekly number of malaria cases reported through the BES in Magude between 2010 and 2014 follows a seasonal pattern with a peak between December and May and a reduction of cases during the dry season (Fig 2B). According to BES data, there were 293 cases per 1000 population at risk

For peer review only - http://bmjopen.bmj.com/site/about/guidelines.xhtml

reported during the transmission year of July 2011 - June 2012, 247 per 1000 between July 2012 and June 2013, 252 cases per 1000 between July 2013 - June 2014, and 110 cases per 1,000 between July 2014 and June 2015. [7].

The prevalence of history of fever during the 30 days prior to the baseline census was 12.1% in children under the age of 5, 7.7% among 5-14 year-olds, and 11.6% in individuals older than 15. Almost all individuals reported going to a health facility as their primary source of health care (>99%), while a small proportion mentioned seeking care first from traditional healers or community health workers. The proportion of individuals who reported sleeping under a bed net the night before was 27.1% among children under the age of 5, 21.8% in 5-14 year-olds, and 27% among those > 15 years of age (Table 3). The universal bed-net coverage (i.e. one net for every two individuals of a household) in 2015 was 52.7% in the administrative post of Magude-Sede, 59.9% in Motaze, 44.1% in Panjane, 52.7% in Mahele and 52.1% in Mapulanguene. Finally, the reported coverage of IRS during the previous 12 months was 49% in Magude-Sede, 79% in Motaze, 41.1% in Panjane, 61% in Mahele, and 30.7% in Mapulanguene (Table 2 and Sup.Table 3).

Discussion

Overall, the Demographic and Health Platform established in Magude offered detailed insight on the baseline socio-demographic profile of Magude district prior to the Magude project. It revealed that the number of residents in Magude in 2015 (52,804 individuals and 10,965 households) was similar to the number reported by the 2007 national census conducted by the National Institute of Statistics (INE) (53,229 individuals and 11,408 households) [9] but did not coincide with the projected population for Magude in 2015 estimated by the INE (n=62,000) [10]. The census update conducted in 2016 identified individuals who had been missed by the baseline census, and recorded the births, deaths, and in-migrations since baseline. As a result, the population of Magude in 2016 assuming that those censed in 2015 who were not found in 2016 were still living in the district, was estimated to be 61,868 individuals, which is similar to the projection by INE for this year (62,924).

For peer review only - http://bmjopen.bmj.com/site/about/guidelines.xhtml

The usefulness of a Demographic and Health Surveillance System in demographic and bio-medical research in African countries has been previously described [16]. Having a precise source of population data is crucial for the correct estimation of disease risk and coverage of health indicators in a population, as well as for planning purposes of public health interventions [17]. This platform has shown that detailed population data collected at individual level at one point in time still misses a number of individuals, who can be captured after a later update. However, all census rounds are also limited by the capacity to find individuals at home, or informants of unavailable individuals, to identify whether a member lives in the area despite not being found during a specific census round. To identify or collect information about absent individuals, field supervisors attempt to arrange interviews with them at their places of work, or interview their family members at their households during weekends. The same phenomenon is expected to take place when implementing malaria interventions in the community (such as rounds of IRS or mass drug administrations), which generally challenges the estimation of intervention coverage.

Household-level data indicates that the households in Magude are typical of a rural area with suboptimal conditions regarding access to water, electricity, and sanitation. The majority of houses were relatively homogeneous, built with non-durable materials, such as cane and adobe, and did not have access to clean water, conditions indicative of a low socio-economic status [18]. The age and sex composition of Magude's population is typical of rural Mozambique and sub-Saharan Africa, composed primarily by young individuals and a decreasing proportion of adults as age increases [19]. Another important aspect of the structure of the population of Magude is the lower sex ratio (number of males in relation to that of females) among young and middle-aged adults, which has been reported, in the neighbouring district of Manhiça, to be related to male labour migration to Maputo city or South Africa and higher male mortality [19].

A large proportion of the heads of households and of the overall population of Magude reported being illiterate, a major risk factor for health outcomes. Additionally, most individuals reported having occupations related to agriculture or fishing, which are usually carried out early in

For peer review only - http://bmjopen.bmj.com/site/about/guidelines.xhtml

the morning until around noon. These aspects should be taken into consideration when planning mobilization campaigns and community-based interventions, as visits might have to be conducted at certain times of day and/or venues outside the households to be able to find these individuals.

Magude is also subject to significant mobility and migration, as more than half of the population reported travelling frequently outside of the district. The destinations most reported by travelers living in Magude were Maputo and South Africa, followed by Gaza and Inhambane provinces. This information is useful to evaluate the risk of malaria importation from other areas due to travel or migration [20]. Maputo city and South Africa are areas with lower malaria burden than Magude [21]; however, the districts surrounding Magude (Manhiça, Moamba), the provinces of Gaza, Inhambane and the rest of Mozambique have higher malaria prevalence estimates than Magude district [2], and could be a source of imported infections [22]. These places are commonly visited by all age groups, whereas the lower-endemic areas are mainly visited by <30 year-olds. This age pattern of migration is typical of the rural areas of Southern Mozambique, where migration rates are higher among individuals aged 20-45 and their children, and decrease with increasing age when individuals establish as permanent residents in an area.

The population of Magude has access to ten health facilities, one of them with an inpatient ward with 57 beds. Thus there are 11.8 beds per 10,000 population, a ratio that is higher than the national level (7 beds per 10,000 in Mozambique) [23]. While most respondents indicated seeking health care primarily from formal health facilities, the majority of the population is scattered throughout the district as far as 38 kms away from a HF and it is difficult to access health facilities or APEs due to limited roads and lack of transportation. This has implications for the delivery of public health interventions, especially for those that rely on the passive detection of cases to control transmission in the community.

Data between 2011 and 2014 from the outpatient and inpatient department of the Magude Sede health center indicated that malaria was the main cause of hospitalization and later discharge with a clear seasonal pattern. The population of Magude is also burdened by HIV, anemia, pneumonia, and

For peer review only - http://bmjopen.bmj.com/site/about/guidelines.xhtml

diarrhea, similar to its neighboring districts of Manhiça and Chokwe [24-31]. Inpatient information was retrospectively collected, and thus, is limited by the quality and accuracy of the data at the time at which it was collected. It may also be biased by disease-specific interventions or changes in diagnostics, referral protocols of severe cases to other districts, or reporting practices. Additionally, cases reported by BES are subject to RDTs stock-outs or reporting inaccuracies. Cases reported by the APEs are usually excluded, and neither the total number of fevers nor the number of individuals tested are reported. Thus, a stronger surveillance system than BES is required to fully capture the clinical malaria profile of a district aiming to eliminate malaria [32].

The age-specific self-reported history of fever in the past 30 days (7.7%-12.1%) were slightly lower than the average percentage of fevers reported in the past 2 weeks in Maputo Province, which was 15% according to the most recent malaria indicator survey [2]. Approximately half of the households in Magude reported owning one bed net for every two individuals (universal coverage). However, only a fourth of the population reported sleeping under a bed net the previous night. This information is indicative of an area where bed net owners do not necessarily use the net as a preventive tool against malaria during the rainy season, despite this being a prevalent disease in the district. Other demographic and socio-economic risk factors that have been associated with LLIN use, such as households with more children, an undereducated household head, or households far away from the health facilities, might explain the low bed net usage in Magude [33-35].

The reported IRS coverage in the area of Motaze was higher, which corresponds with the focal IRS campaign conducted by the NMCP that took place only there in September of 2014. The coverage reported in other areas was probably the consequence of recall bias, as a district-wide IRS campaign took place in 2013. Overall, the coverage reported for LLIN and IRS throughout the district was below the World Health Organization recommended coverage of >80% [36], leaving a significant proportion of the population unprotected by any of the standard preventative measures. These findings mirror the estimates reported for Maputo Province in 2011 by the Demographic Health Survey, which found a reported bed net usage rate of 27% in Maputo Province [37]. The identification of such vector control coverage gaps

For peer review only - http://bmjopen.bmj.com/site/about/guidelines.xhtml

called for a strong community engagement campaign focused on the use of the bed nets and participation in the yearly rounds of IRS.

Conclusion

Through the establishment of a Demographic and Health Platform in Magude in 2015, which was updated in 2016, and a baseline assessment of the relevant health indicators, it was possible to fully characterize a district where malaria elimination interventions were to be deployed and evaluated. Magude represents a typical rural district of Mozambique characterized by limited social and economic infrastructures, which had to be considered for the design and operationalization of the community-based interventions. This study also revealed low literacy rates among a large proportion of the population and identified agriculture as the main economic activity in the district. These socio-demographic characteristics suggest that a strong community engagement would be necessary for the implementation of a malaria elimination project. Half of the population of Magude reported travelling outside of the district to areas of high and low malaria transmission intensity, showing that malaria importation will likely be a source of continuous transmission even if interruption of local transmission is achieved. Also, the poor access to health care services, as well as to core malaria vector control interventions such as IRS and bed nets distributed by the NMCP, indicated that these aspects needed to be integrated within the malaria elimination program planned for the district, in order to maximize the impact of the interventions aiming to interrupt transmission. Overall, this district represents the reality of the majority of malaria endemic areas in Sub-Saharan Africa, where elimination is most needed, and where it is most pressing to demonstrate the feasibility of elimination strategies.

For peer review only - http://bmjopen.bmj.com/site/about/guidelines.xhtml

Abbreviations

APEs – Agentes Polivalentes Elementais (Community Health Workers)

BES – Boletim Epidemiológico Semanal (Weekly Epidemiological Bulletin)

CISM – Manhiça Health Research Center

DHP – Demographic and Health Platform

GPS – Global Positioning System

HDSS – Health and Demographic Surveillance System

HF – Health Facility

HIV – Human Immunodeficiency virus

INE – Instituto Nacional de Estatística (National Institute of Statistics)

IPTp – Intermittent Preventive Treatment in pregnancy

IRS – indoor residual spraying

ISGlobal – Barcelona Institute of Global Health

LLINs – Long-Lasting Insecticide treated Nets

MDAs – Mass Drug Administration

NMCP - National Malaria Control Program of Mozambique

RDTs – Rapid Diagnostic Tests

TV – Television

Declarations

Acknowledgements

We would like to thank the community of Magude for participating in this study, and the team of field workers who have collected the data presented here. We would also like to acknowledge the Administrative and District Health Authorities of Magude for their collaboration and for providing some of the information also included in this article. We thank everyone who supported this study directly or indirectly through fieldwork, or analysis support.

Ethics approval and consent to participate

The Demographic and Health Platform protocol, consent forms and questionnaires were approved by the CISM internal ethics committee and written consent from the health authorities was also sought prior to its implementation. Meetings were held with community leaders and with general members of the community of Magude to inform them about the DHP operations and the malaria elimination project as a whole. A written informed consent

For peer review only - http://bmjopen.bmj.com/site/about/guidelines.xhtml

was obtained from all household heads to record household-level information, as well as from all individuals providing individual information. Informed consents for children under the age of 18 years were sought from their parents or primary caretakers. The collection of district health surveillance data was conducted under the protocol that aimed to evaluate the impact of the *Magude project* on malaria transmission, which was approved by CISM's internal ethical committee, the Ethics Committee of the Hospital Clínic of Barcelona, and the Ministry of Health National Bioethics Committee of Mozambique (IRB00002657).

Competing Interests

The authors declare that they have no competing interests

Funding

Funding was provided by the Bill and Melinda Gates Foundation and the Fundación "la Caixa" Partnership for the Elimination of Malaria in Southern Mozambique (OPP1115265). Q. B. is an ICREA (Institut Catal. de la Recerca i Estudis Avan.ats; Catalan Government) Research Professor. ISGlobal is a member of the CERCA Programme, Generalitat de Catalunya.

Author's Contributions

BG: participated in the study design and fieldwork, supported in the data cleaning and data analysis process, and wrote the draft of this article

AN: participated in the study design, in study analyses, in the interpretation of results and writing of this article

HMS: cleaned and analyzed the data, and contributed to the writing of this article

HM: Led the implementation of field activities and data collection process, and participated in the interpretation of results

EJ: Participated in the study design and implementation of field activities and data collection.

CG: Participated in the interpretation of results and writing of this article

FA: Designed the data collection tools, supported the implementation of field activities and data cleaning.

EM: participated in the study design, and interpretation of results

QB: participated in the study design, interpretation of results and writing of this article

For peer review only

For peer review only - http://bmjopen.bmj.com/site/about/guidelines.xhtml

NRR: participated in the interpretation of results and writing of this article

PLA: participated in the study design, interpretation of results and writing of this article

PA: participated in the study design, field implementation interpretation of results and writing of this article

FS: participated in the study design, supervised field activities, interpretation of results and writing of this article

CS: Conceived the study, participated in interpretation of results and writing of this article

Availability of data

The datasets collected as part of the Demographic Health Platform and analysed during the current study are available from the corresponding author upon written request.

The data presented in figures 2a and 2b belongs to the Ministry of Health of Mozambique and is considered as third-party data which can be accessed by contacting the following individuals appointed by the Ministry of health:

- To gain access to Inpatient Department data collected by the Ministry of health please contact Dr Baltazar Candrinho, Head of NMCP Mozambique, phone number:+258828665730 or e-mail: candrinhobaltazar@gmail.com
- To gain access to the data collected through the Boletim Epidemiologico Semanal (BES) please contact Dr Lorna Gujral, Department of Epidemiology/MOH/Mozambique, phone number: +25882 3250800 or e-mail: lornita15.lgg@gmail.com

For peer review only - http://bmjopen.bmj.com/site/about/guidelines.xhtml

References

1. World Health Organization. World malaria report 2018. :210.

2. Ministério da Saúde (MISAU), Instituto Nacional de Estatística (INE), ICF Internacional. Inquérito de Indicadores de Imunização, Malária e HIV/SIDA em Moçambique 2015. Maputo. Moçambique. Rockville, Maryland, EUA: INS, INE e ICF International; 2015.

3. World Health Organization, others. A framework for malaria elimination. 2017 [cited 2017 Mar 28]; Available from: http://apps.who.int/iris/handle/10665/254761

4. Aide P, Candrinho B, Galatas B, Munguambe K, Guinovart C, Luis F, et al. Setting the scene and generating evidence for malaria elimination in Southern Mozambique. Malar J. 2019;18:190.

5. Instituto Nacional de Estatistica. Relatório Final do Inquérito ao Orçamento Familiar- IOF 2014/15 [Internet]. 2015. Available from: http://www.ine.gov.mz/operacoes-estatisticas/inqueritos/inquerito-sobre-orcamento-familiar

6. Maharaj R, Moonasar D, Baltazar C, Kunene S, Morris N. Sustaining control: lessons from the Lubombo spatial development initiative in southern Africa. Malar J [Internet]. 2016 [cited 2016 Oct 17];15. Available from: http://malariajournal.biomedcentral.com/articles/10.1186/s12936-016-1453-9

7. Ministerio de Administração Estatal. Perfil do distrito de Magude, Provincia de Maputo (2014). Ministerio de Administração Estatal; 2014.

8. Alonso P, Saúte F, Aponte J, Gómez-Olivé F, Nhacolo A, Thompson R, et al. Manhiça demographic surveillance system, Mozambique. Popul Health Surviv INDEPTH Sites. 2002;1:295–308.

9. Instituto Nacional de Estatística. Estatísticas do Distrito de Magude. 2010.

10. Instituto Nacional de Estatistica. População Projectada por distritos, Maputo Província 2007_2040.xls — Instituto Nacional de Estatistica [Internet]. [cited 2018 May 29]. Available from: http://www.ine.gov.mz/estatisticas/estatisticas-demograficas-e-indicadores-sociais/projeccoes-da-populacao/populacao-projectada-por-distritos-maputo-provincia-2007_2040.xls/view

11. Portugaliza HP, Galatas B, Nhantumbo H, Djive H, Murato I, Saúte F, et al. Examining community perceptions of malaria to inform elimination efforts

For peer review only - http://bmjopen.bmj.com/site/about/guidelines.xhtml

in Southern Mozambique: a qualitative study. Malar J [Internet]. 2019 [cited 2019 Aug 21];18. Available from: https://malariajournal.biomedcentral.com/articles/10.1186/s12936-019-2867-y

12. R, Foundation for Statistical Computing. R: A language and environment for statistical computing [Internet]. Vienna, Austria; Available from: http://www.R-project.org/

13. Hijmans R, Cruz M, Rojas E, Guarino L. DIVA-GIS, version 1.4. A geographic information system for the management and analysis of genetic resources data. 2001;

14. QGIS Development Team (2017) [Internet]. Open Source Geospatial Foundation Project; Available from: http://qgis.osgeo.org

15. Ndima SD, Sidat M, Give C, Ormel H, Kok MC, Taegtmeyer M. Supervision of community health workers in Mozambique: a qualitative study of factors influencing motivation and programme implementation. Hum Resour Health. 2015;13:63.

16. Bocquier P, Sankoh O, Byass P. Are health and demographic surveillance system estimates sufficiently generalisable? Glob Health Action. 2017;10:1356621.

17. Tatem AJ, Campiz N, Gething PW, Snow RW, Linard C. The effects of spatial population dataset choice on estimates of population at risk of disease. Popul Health Metr. 2011;9:4.

18. Pons-Duran C, González R, Quintó L, Munguambe K, Tallada J, Naniche D, et al. Association between HIV infection and socio-economic status: evidence from a semirural area of southern Mozambique. Trop Med Int Health. 2016;21:1513–21.

19. Nhacolo AQ, Nhalungo DA, Sacoor CN, Aponte JJ, Thompson R, Alonso P. Levels and trends of demographic indices in southern rural Mozambique: evidence from demographic surveillance in Manhiça district. BMC Public Health [Internet]. 2006 [cited 2017 Jun 6];6. Available from: http://bmcpublichealth.biomedcentral.com/articles/10.1186/1471-2458-6-291

20. Ruktanonchai NW, Bhavnani D, Sorichetta A, Bengtsson L, Carter KH, Córdoba RC, et al. Census-derived migration data as a tool for informing malaria elimination policy. Malar J. 2016;15:273.

21. Moonasar D, Maharaj R, Kunene S, Candrinho B, Saute F, Ntshalintshali N, et al. Towards malaria elimination in the MOSASWA (Mozambique, South Africa and Swaziland) region. Malar J [Internet]. 2016 [cited 2016 Aug

For peer review only

For peer review only - http://bmjopen.bmj.com/site/about/guidelines.xhtml

25];15. Available from: http://malariajournal.biomedcentral.com/articles/10.1186/s12936-016-1470-8

22. Ruktanonchai NW, Smith DL, De Leenheer P. Parasite sources and sinks in a patched Ross-Macdonald malaria model with human and mosquito movement: Implications for control. Math Biosci. 2016;279:90–101.

23. World Health Organization. GHO | By country | Mozambique - statistics summary (2002 - present) [Internet]. WHO. [cited 2018 May 29]. Available from: http://apps.who.int/gho/data/node.country.country-MOZ?lang=en

24. González R, Augusto OJ, Munguambe K, Pierrat C, Pedro EN, Sacoor C, et al. HIV Incidence and Spatial Clustering in a Rural Area of Southern Mozambique. PloS One. 2015;10:e0132053.

25. Feldblum PJ, Enosse S, Dubé K, Arnaldo P, Muluana C, Banze R, et al. HIV prevalence and incidence in a cohort of women at higher risk for HIV acquisition in Chókwè, southern Mozambique. PloS One. 2014;9:e97547.

26. Deus N de, João E, Cuamba A, Cassocera M, Luís L, Acácio S, et al. Epidemiology of Rotavirus Infection in Children from a Rural and Urban Area, in Maputo, Southern Mozambique, before Vaccine Introduction. J Trop Pediatr. 2018;64:141–5.

27. Nhampossa T, Mandomando I, Acacio S, Quintó L, Vubil D, Ruiz J, et al. Diarrheal Disease in Rural Mozambique: Burden, Risk Factors and Etiology of Diarrheal Disease among Children Aged 0-59 Months Seeking Care at Health Facilities. PloS One. 2015;10:e0119824.

28. Aguilar R, Moraleda C, Quintó L, Renom M, Mussacate L, Macete E, et al. Challenges in the diagnosis of iron deficiency in children exposed to high prevalence of infections. PloS One. 2012;7:e50584.

29. Garcia-Basteiro AL, Miranda Ribeiro R, Brew J, Sacoor C, Valencia S, Bulo H, et al. Tuberculosis on the rise in southern Mozambique (1997-2012). Eur Respir J. 2017;49.

30. Lanaspa M, O'Callaghan-Gordo C, Machevo S, Madrid L, Nhampossa T, Acácio S, et al. High prevalence of Pneumocystis jirovecii pneumonia among Mozambican children <5 years of age admitted to hospital with clinical severe pneumonia. Clin Microbiol Infect Off Publ Eur Soc Clin Microbiol Infect Dis. 2015;21:1018.e9-1018.e15.

31. Roca A, Sigauque B, Quinto L, Morais L, Berenguera A, Corachan M, et al. Estimating the vaccine-preventable burden of hospitalized pneumonia among young Mozambican children. Vaccine. 2010;28:4851–7.

For peer review only

For peer review only - http://bmjopen.bmj.com/site/about/guidelines.xhtml

32. Barclay VC, Smith RA, Findeis JL. Surveillance considerations for malaria elimination. Malar J. 2012;11:304.

33. Pinchoff J, Hamapumbu H, Kobayashi T, Simubali L, Stevenson JC, Norris DE, et al. Factors Associated with Sustained Use of Long-Lasting Insecticide-Treated Nets Following a Reduction in Malaria Transmission in Southern Zambia. Am J Trop Med Hyg. 2015;93:954–60.

34. García-Basteiro AL, Schwabe C, Aragon C, Baltazar G, Rehman AM, Matias A, et al. Determinants of bed net use in children under five and household bed net ownership on Bioko Island, Equatorial Guinea. Malar J. 2011;10:179.

35. Atieli HE, Zhou G, Afrane Y, Lee M-C, Mwanzo I, Githeko AK, et al. Insecticide-treated net (ITN) ownership, usage, and malaria transmission in the highlands of western Kenya. Parasit Vectors. 2011;4:113.

36. World Health Organization, World Health Organization, Global Malaria Programme. Global technical strategy for malaria, 2016-2030 [Internet]. 2015 [cited 2018 Mar 5]. Available from: http://apps.who.int/iris/bitstream/10665/176712/1/9789241564991_eng.pdf?ua=1

37. Ministerio da Saude (MISAU), Instituto Nacional de Estatística (INE) e ICF International (ICFI). Moçambique Inquérito Demográfico e de Saúde 2011. Calverton Md USA MISAU INE E ICFI. 2011;

For peer review only - http://bmjopen.bmj.com/site/about/guidelines.xhtml

Tables

Table 1: Summary of Magude Population in 2015 and 2016.

	District Level		Magude Sede		Motaze		Panjane		Mahele		Mapulan-guene	
	N	%	N	%	N	%	N	%	N	%	N	%
**Households 2015**	10965		8011	73.1	1471	13.4	627	5.7	377	3.4	479	4.4
**Households 2016**	11960		8520	71.3	1441	12.1	645	5.4	803	6.7	547	4.6
**Population 2015**	52802		38534	73.0	7421	14.1	3122	5.9	1784	3.4	1934	3.7
Residents	48448	91.8*	35346	73.0	6605	13.6	2930	6.0	1695	3.5	1868	3.9
Non-residents	4133	7.8*	2970	71.9	816	19.7	191	4.6	89	2.2	66	1.6
Unclassified	221	0.4*	218	99.5	0	-	1	0.5	0	-	0	-
**Population 2016**	61868		44203	71.4	8149	13.2	3576	5.8	3499	5.7	2404	3.8
Residents	56943 ±	92.1*	40623	71.4	7275	12.8	3361	5.9	3329	5.8	2322	4.1
Non-residents	4535 ø	7.3*	3266	72.0	842	18.6	206	4.5	143	3.2	76	1.7
Unclassified	390 §	0.6*	314	80.9	32	8.2	9	2.3	27	7.0	6	1.5
**Demographic Events 2015-16**	3078		2204	71.6	390	12.7	186	6.0	168	5.5	129	4.2
Births	1687	54.8	1203	71.4	220	13.0	92	5.5	91	5.4	80	4.7
Immigrations	721	23.4	521	72.3	60	8.3	51	7.1	60	8.3	29	4.0
Deaths	670	21.8	480	71.6	110	16.4	43	6.4	17	2.5	20	3.0

* Column %
± 56,943 = 48,448 (censed in 2015) – 616 (deaths) + 1,670 (births) + 1,235 (immigrations) + 6,206 (censed in 2016)
ø 4,535 = 4,133- 42 (deaths) + 12 (births) + 64 (immigrations) + 368 (censed in 2016)
§ 390 = 221 – 8 (deaths) + 5 (births) + 21 (immigrations) + 151 (censed in 2016)

For peer review only

For peer review only - http://bmjopen.bmj.com/site/about/guidelines.xhtml

871 **Table 2: Socio-demographic characteristics of heads of households, and**
872 **household characteristics (2015).**

**Characteristics of Heads of households***		**N**	**%**
Sex	Males	5051	54.6
	Females	4206	45.4
Age group (years)	< 18	40	0.4
	18 – 24	520	5.6
	25 – 64	6919	74.7
	> = 65	1777	19.2
Residents of Magude		9257	84.4
Education level (>=6 years old)	Illiterate	5460	59.0
	5-7th grade	2611	28.2
	8 -12th grade	807	8.7
	University	37	0.4
	Missing Information	342	3.7
Marital status (> 14 years old)	Single	4007	43.3
	Married or de-facto union	3275	35.4
	Divorced	16	0.2
	Separated	393	4.3
	Widow	1554	16.8
**Households characteristics**			
Average household size**		5 [3-7]	
Lone-resident households		1171	10.7
Wall material of the main building	Cane	3506	32.5
	Cement	2807	26.0
	Mud bricks	2338	21.6
	Adobe	1703	15.8
	Zinc plates	319	3.0
	Wood	128	1.2
Type of toilet	Traditional latrine	5774	53.5
	Improved latrine	1144	10.6
	WC connected to septic tank	233	2.2
	No latrine	3650	33.8
Main source of lighting energy	Paraffin	5362	49.6
	Electricity	3321	30.8
	Candles	1074	9.9
	Solar panels	502	4.6
	Other	539	5.0
Kitchen Fuel	Wood logs	6401	59.3
	Coal	740	6.9
	Gas	46	0.4
	Electricity	65	0.6
	Paraffin	8	0.1
	Missing Information	3541	32,8
Primary source of drinking water	Water pumps	3700	34.3
	Directly from the river	2691	24.9
	Piped water	2218	20.5
	Open well near the river	1138	10.5
	Other	1054	9.8
**Vector control tools**			

For peer review only - http://bmjopen.bmj.com/site/about/guidelines.xhtml

Universal ITN coverage (one net per two household members)		5690	53.2
IRS in past 12 months	Yes	5722	52.2
	No	4719	43.0
	Unknown	524	4.8

* or subhead (if head does not live in the household)

**Median [Interquartile Range]

For peer review only

For peer review only - http://bmjopen.bmj.com/site/about/guidelines.xhtml

Table 3: Individual-level health and malaria prevention indicators in Magude district 2015).

**Age group in Years**	**< 5**		**5-14**		**>15**	
	N	%	N	%	N	%
Had fever in preceding 30 days						
Yes	1043	12.1	1128	7.7	2931	11.6
No	7492	87.2	13325	91.2	21977	87.1
Unknown	55	0.6	154	1.1	319	1.3
Primary source of health care						
Health Facility	8552	99.6	14548	99.6	25075	99.4
Traditional Healer	13	0.2	27	0.2	61	0.2
Community Health Worker	22	0.3	32	0.2	66	0.3
Slept under a bed net the preceding night						
Yes	2330	27.1	3179	21.8	6799	27.0
No	6260	72.9	11428	78.2	18428	73.0

For peer review only

For peer review only - http://bmjopen.bmj.com/site/about/guidelines.xhtml

Figure Titles and Legends

Fig 1: Map of Magude district (2015).

A) Administrative and permanent river shape files were obtained from DIVA-GIS ((http://www.diva-gis.org/Data), and confirmed with Magude's key informants. GPS positions of households, health facilities and community health workers obtained directly from the field and mapped using QGIS. B) Population pyramid of Magude district (2015). Proportions out of the total population were calculated for every five-year age and sex group

Fig 2: Health profile of Magude district prior to the baseline census (2010-2014).

A) Most common diseases leading to all-age hospitalization and later discharge in Magude reported by the District Health Authorities between 2010-2014. B) Weekly number of outpatient malaria cases observed in Magude between 2010 and 2014 reported through the Weekly Epidemiological Bulletin (BES).

For peer review only - http://bmjopen.bmj.com/site/about/guidelines.xhtml

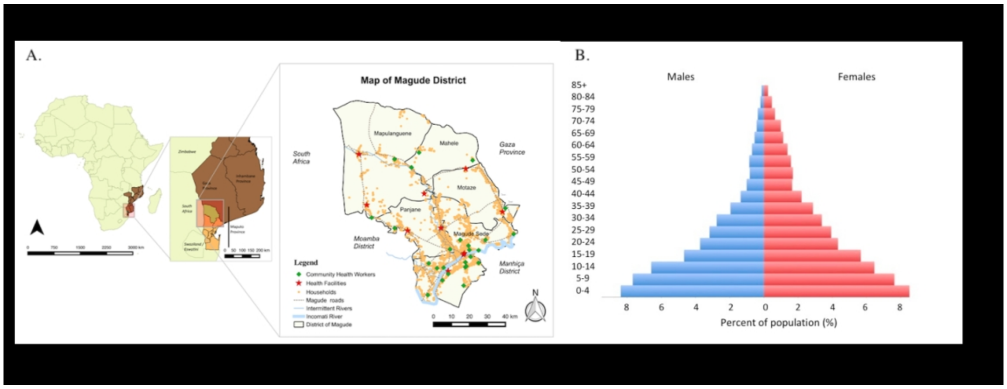
A.
Map of Magude District
Mapulanguene
Mahele
Motaze
Panjane
Magude Sede
South Africa
Gaza Province
Moamba District
Manhiça District
Legend
Community Health Workers
Health Facilities
Households
Magude roads
Intermittent Rivers
Incomati River
District of Magude
0 10 20 30 40 km
N
B.
Males
Females
85+
80-84
75-79
70-74
65-69
60-64
55-59
50-54
45-49
40-44
35-39
30-34
25-29
20-24
15-19
10-14
5-9
0-4
8 6 4 2 0 2 4 6 8
Percent of population (%)

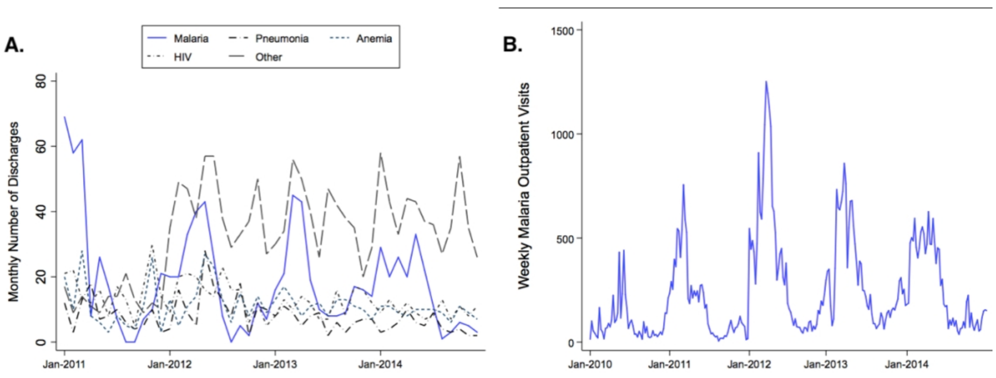
A.
Malaria
Pneumonia
Anemia
HIV
Other
Monthly Number of Discharges
0
20
40
60
80
Jan-2011
Jan-2012
Jan-2013
Jan-2014
B.
Weekly Malaria Outpatient Visits
0
500
1000
1500
Jan-2010
Jan-2011
Jan-2012
Jan-2013
Jan-2014

Supplementary Tables

Sup.Table 1: **Selected demographic and socioeconomic characteristics of Magude's population (2015).**

Variable / by sex	Males		Females	
	N	Col %	N	Col %
**Age groups (years)**	21775		26673	
< 1	826	3.8	898	3.4
1-4	3391	15.6	3475	13.0
5-14	7345	33.8	7262	27.2
15-64	9334	42.9	13205	49.5
>=65	864	4.0	1824	6.8
**Education Level (>= 6 years old)**	16712		21461	
Illiterate	7925	47.4	11136	51.9
5-9 grade	6221	37.2	7616	35.5
10-12 grade	1595	9.5	1825	8.5
University	43	0.3	20	0.1
Missing Information	928	5.6	864	4.0
**Occupation (> 18 years old)**	5243		5532	
Farmer or Fisherman	1371	26.1	3909	70.7
Salesperson	466	8.9	609	11.0
Construction work occupations	1159	22.1	39	0.7
Coal maker / Lumberjack	603	11.5	173	3.1
Guards/Police/Military	539	10.3	45	0.8
Health professional	85	1.6	133	2.4
Teacher	263	5.0	180	3.3
Miner	106	2.0	9	0.2
Other	651	12.4	435	7.9
**Marital Status (> 14 years old)**	10157		14986	
Single (never married)	6168	60.7	7772	51.9
Married or de-facto union	3690	36.3	4702	31.4
In a polygamic relationship	202	2.0	-	-
Divorced	7	0.1	13	0.1
Separated	138	1.4	541	3.6
Widow	154	1.5	1958	13.1

For peer review only

Sup.Table 2: Number of individuals by age group who reported spending one night outside of Magude the day before the census visit was performed (2015).

		Men		Women		All	
Age group	Previous night spent outside of Magude	N	(%)	N	(%)	N	(%)
0 - 14	Yes	592	5.1	580	5.0	1172	43,0 *
	No	10874	94.0	10970	94.3		
	NA±	96	0.8	85	0.7		
15 – 29	Yes	324	6.3	362	5.6	686	25.1 *
	No	4774	92.7	6022	93.3		
	NA±	52	1.0	70	1.1		
30 – 44	Yes	298	11.3	197	5.3	495	18.1 *
	No	2323	87.8	3497	93.9		
	NA±	24	0.9	30	0.8		
45 – 64	Yes	133	8.6	149	4.9	282	10.3 *
	No	1393	90.5	2839	93.8		
	NA±	13	0.8	39	1.3		
> 65	Yes	36	4.2	57	3.1	93	3.4*
	No	818	94.7	1744	95.6		
	NA±	10	1.2	23	1.3		
All ages	Yes	1383	6.4	1345	5.0	2728	5.6
	No	20182	92.7	25072	94,0	45254	93.5
	NA±	195	0.9	247	1.0	442	0.9

* Percentage of those who reported travelling (2,728)
± Missing Information

Sup.Table 3: **Household-level health and malaria prevention indicators in Magude district (2015).**

Administrative Post	Magude-Sede		Motaze		Panjane		Mahele		Mapulanguene	
Household size	4 [3-6]		4 [3-7]		4 [2-6]		4 [2-6]		3 [2-5.5]	
Members per net Median [IQR]	2.0 [1.4-4.0]		2.0 [1.3-3.0]		2.0 [1.6-5.5]		2.0 [1.5-4.0]		2.0 [1.3-4.0]	
Universal ITN coverage *	52.7%		59.9%		44.1%		52.7%		52.1%	
IRS in past 12 months	N	%	N	%	N	%	N	%	N	%
Yes	3925	49.0	1162	79.0	258	41.1	230	61.0	147	30.7
No	3627	45.3	293	19.9	352	56.1	133	35.3	314	65.6
Unknown	459	5.7	16	1.1	17	2.7	14	3.7	18	3.8

** One net for every 2 members of the households

Supplementary Figures

Sup.Figure 1: Household Assets in Magude district in 2015.

Percentage of households in Magude district with at least one item per asset, and median and interquartile range of the assets for which the number of items available was higher than 1.

Sup.Figure 2: Mobility patterns of Magude's population in 2015.

Main destinations per age group among those who reported travelling outside of Magude in 2015.

For peer review only

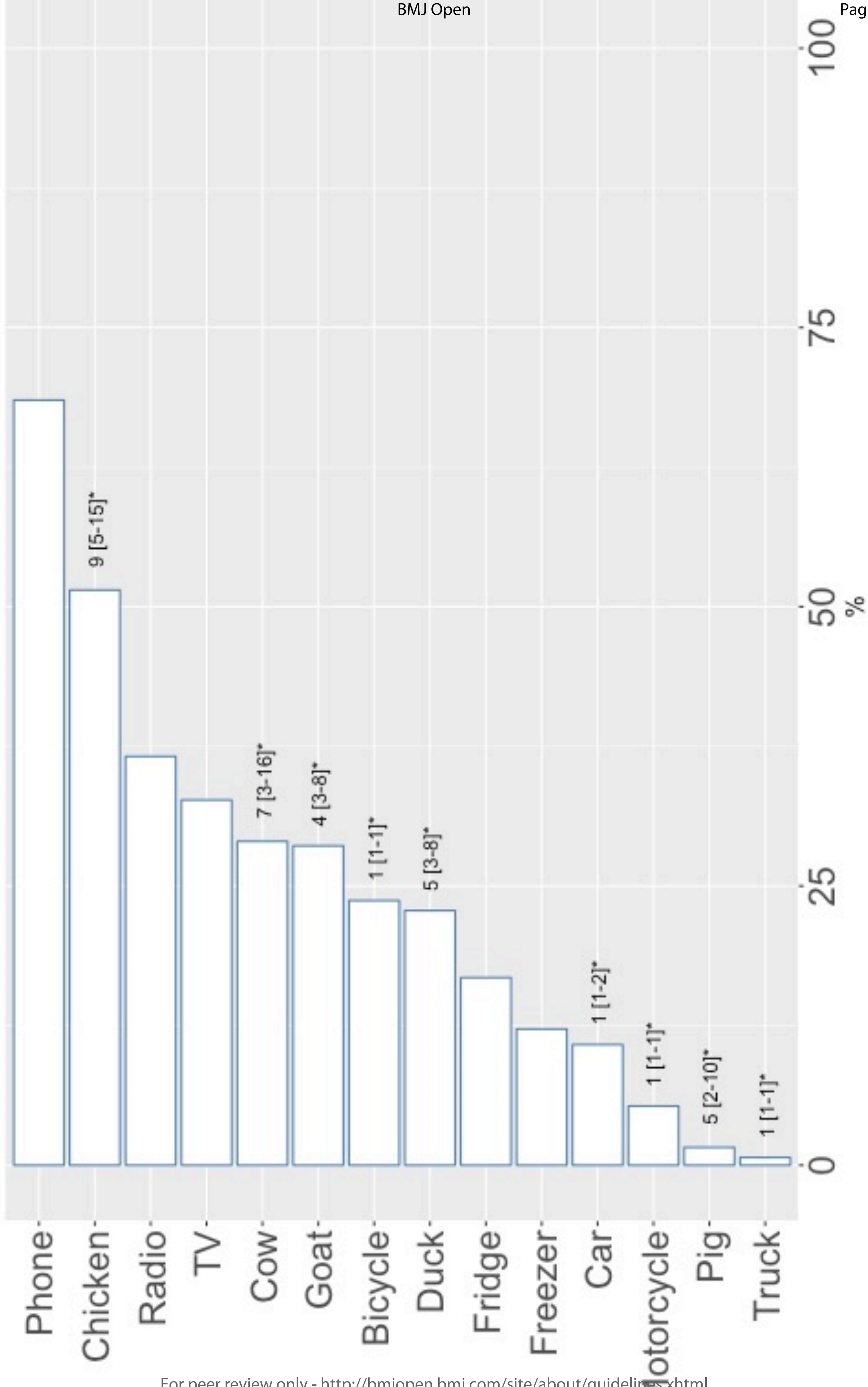
Phone
Chicken
Radio
TV
Cow
Goat
Bicycle
Duck
Fridge
Freezer
Car
Motorcycle
Pig
Truck
9 [5-15]*
7 [3-16]*
4 [3-8]*
1 [1-1]*
5 [3-8]*
1 [1-2]*
1 [1-1]*
5 [2-10]*
1 [1-1]*
0
25
50
75
100
%

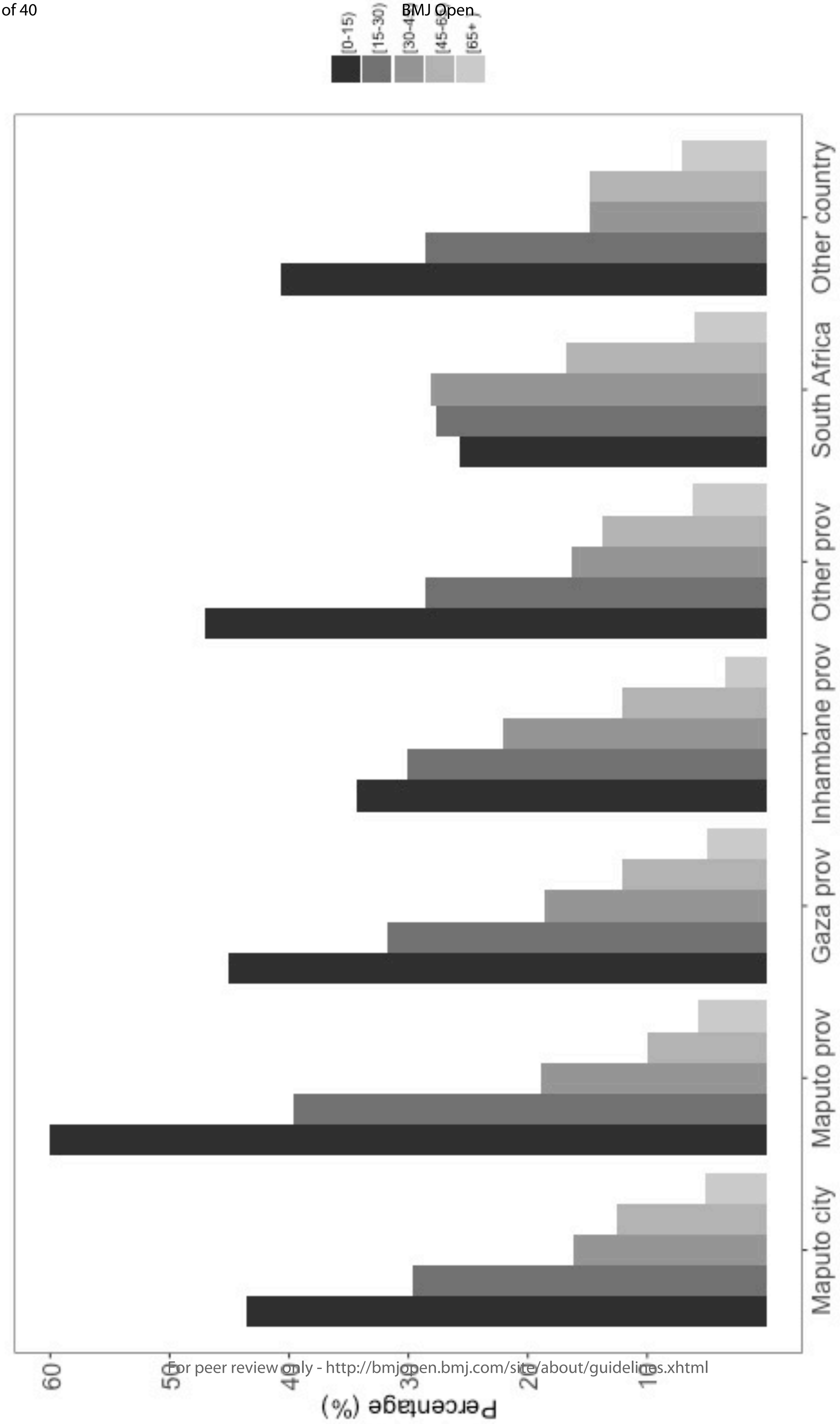
[0-15)
[15-30)
Other country
South Africa
Other prov
Inhambane prov
Gaza prov
Maputo prov
Maputo city
60
50
40
30
20
10
Percentage (%)

STROBE Statement—Checklist of items that should be included in reports of ***cross-sectional studies***

	Item No	Recommendation	Check
**Title and abstract**	1	(*a*) Indicate the study's design with a commonly used term in the title or the abstract	Yes
		(*b*) Provide in the abstract an informative and balanced summary of what was done and what was found	Yes
**Introduction**			
Background/rationale	2	Explain the scientific background and rationale for the investigation being reported	Yes
Objectives	3	State specific objectives, including any prespecified hypotheses	Yes
**Methods**			
Study design	4	Present key elements of study design early in the paper	Yes
Setting	5	Describe the setting, locations, and relevant dates, including periods of recruitment, exposure, follow-up, and data collection	Yes
Participants	6	(*a*) Give the eligibility criteria, and the sources and methods of selection of participants	Yes
Variables	7	Clearly define all outcomes, exposures, predictors, potential confounders, and effect modifiers. Give diagnostic criteria, if applicable	NA
Data sources/ measurement	8*	For each variable of interest, give sources of data and details of methods of assessment (measurement). Describe comparability of assessment methods if there is more than one group	Yes
Bias	9	Describe any efforts to address potential sources of bias	Yes
Study size	10	Explain how the study size was arrived at	NA
Quantitative variables	11	Explain how quantitative variables were handled in the analyses. If applicable, describe which groupings were chosen and why	Yes
Statistical methods	12	(*a*) Describe all statistical methods, including those used to control for confounding	Yes
		(*b*) Describe any methods used to examine subgroups and interactions	NA
		(*c*) Explain how missing data were addressed	Yes
		(*d*) If applicable, describe analytical methods taking account of sampling strategy	Yes
		(*e*) Describe any sensitivity analyses	NA
**Results**			
Participants	13*	(a) Report numbers of individuals at each stage of study—eg numbers potentially eligible, examined for eligibility, confirmed eligible, included in the study, completing follow-up, and analysed	Yes
		(b) Give reasons for non-participation at each stage	Yes
		(c) Consider use of a flow diagram	Yes
Descriptive data	14*	(a) Give characteristics of study participants (eg demographic, clinical, social) and information on exposures and potential confounders	Yes
		(b) Indicate number of participants with missing data for each variable of interest	Yes
Outcome data	15*	Report numbers of outcome events or summary measures	Yes
Main results	16	(*a*) Give unadjusted estimates and, if applicable, confounder-adjusted estimates and their precision (eg, 95% confidence interval). Make clear which confounders were adjusted for and why they were included	NA

For peer review only - http://bmjopen.bmj.com/site/about/guidelines.xhtml

		(*b*) Report category boundaries when continuous variables were categorized	Yes
		(*c*) If relevant, consider translating estimates of relative risk into absolute risk for a meaningful time period	NA
Other analyses	17	Report other analyses done—eg analyses of subgroups and interactions, and sensitivity analyses	Yes
**Discussion**			
Key results	18	Summarise key results with reference to study objectives	Yes
Limitations	19	Discuss limitations of the study, taking into account sources of potential bias or imprecision. Discuss both direction and magnitude of any potential bias	Yes
Interpretation	20	Give a cautious overall interpretation of results considering objectives, limitations, multiplicity of analyses, results from similar studies, and other relevant evidence	Yes
Generalisability	21	Discuss the generalisability (external validity) of the study results	Yes
**Other information**			
Funding	22	Give the source of funding and the role of the funders for the present study and, if applicable, for the original study on which the present article is based	Yes

*Give information separately for exposed and unexposed groups.

Note: An Explanation and Elaboration article discusses each checklist item and gives methodological background and published examples of transparent reporting. The STROBE checklist is best used in conjunction with this article (freely available on the Web sites of PLoS Medicine at http://www.plosmedicine.org/, Annals of Internal Medicine at http://www.annals.org/, and Epidemiology at http://www.epidem.com/). Information on the STROBE Initiative is available at www.strobe-statement.org.

For peer review only

For peer review only - http://bmjopen.bmj.com/site/about/guidelines.xhtml

BMJ Open

A demographic and health platform to inform a malaria elimination project in Magude district, Southern Mozambique

Journal:	*BMJ Open*
Manuscript ID	bmjopen-2019-033985.R1
Article Type:	Original research
Date Submitted by the Author:	18-Feb-2020
Complete List of Authors:	Galatas, Beatriz; Instituto de Salud Global Barcelona, Malaria Elimination Initiative; Centro de Investigacao em Saude de Manhica, ISGLobal<br>Nhacolo, Ariel; Centro de Investigacao em Saude de Manhica<br>Marti, Helena; Instituto de Salud Global Barcelona, Malaria Elimination Initiative<br>Munguambe, Humberto; Centro de Investigacao em Saude de Manhica<br>Jamise, Edgar; Centro de Investigaçao em Saúde de Manhiça<br>Guinovart, Caterina; Barcelona Institute for Global Health<br>Cirera, Laia; Instituto de Salud Global Barcelona, Malaria Elimination Initiative<br>Amone, Felimone; Centro de Investigacao em Saude de Manhica<br>Macete, Eusebio; Centro de Investigação em Saúde de Manhiça<br>Bassat, Quique; BARCELONA INSTITUTE FOR GLOBAL HEALTH<br>Rabinovich, Regina; Instituto de Salud Global Barcelona, Malaria Elimination Initiative<br>Alonso, Pedro; WHO, GMP<br>Aide, Pedro; Manhiça Health Research Centre<br>Saute, Francisco; Centro de Investigação em Saúde de Manhiça<br>Sacoor, Charfudin; Centro de Investigacao em Saude de Manhica, Department of Demography
**Primary Subject Heading**:	Global health
Secondary Subject Heading:	Epidemiology, Global health, Public health
Keywords:	Public health < INFECTIOUS DISEASES, Demography < TROPICAL MEDICINE, Magude project, Malaria elimination, Population and health

SCHOLARONE™
Manuscripts

I, the Submitting Author has the right to grant and does grant on behalf of all authors of the Work (as defined in the below author licence), an exclusive licence and/or a non-exclusive licence for contributions from authors who are: i) UK Crown employees; ii) where BMJ has agreed a CC-BY licence shall apply, and/or iii) in accordance with the terms applicable for US Federal Government officers or employees acting as part of their official duties; on a worldwide, perpetual, irrevocable, royalty-free basis to BMJ Publishing Group Ltd ("BMJ") its licensees and where the relevant Journal is co-owned by BMJ to the co-owners of the Journal, to publish the Work in this journal and any other BMJ products and to exploit all rights, as set out in our licence.

The Submitting Author accepts and understands that any supply made under these terms is made by BMJ to the Submitting Author unless you are acting as an employee on behalf of your employer or a postgraduate student of an affiliated institution which is paying any applicable article publishing charge ("APC") for Open Access articles. Where the Submitting Author wishes to make the Work available on an Open Access basis (and intends to pay the relevant APC), the terms of reuse of such Open Access shall be governed by a Creative Commons licence – details of these licences and which Creative Commons licence will apply to this Work are set out in our licence referred to above.

Other than as permitted in any relevant BMJ Author's Self Archiving Policies, I confirm this Work has not been accepted for publication elsewhere, is not being considered for publication elsewhere and does not duplicate material already published. I confirm all authors consent to publication of this Work and authorise the granting of this licence.

For peer review only

A demographic and health platform to inform a malaria elimination project in Magude district, Southern Mozambique

Authors

Beatriz Galatas[1, 2*¶], Ariel Nhacolo¶[1], Helena Martí-Soler[1,2], Humberto Munguambe[1], Edgar Jamise[1], Caterina Guinovart[2], Laia Cirera[1,2], Felimone Amone[1], Eusébio Macete[1,3], Quique Bassat[1,2,5,6], N. Regina Rabinovich[2,7], Pedro L. Alonso[1,2.], Pedro Aide[1,4], Francisco Saute[1], Charfudin Sacoor[1]

Affiliations

1. Centro de Investigação em Saúde da Manhiça (CISM), Manhiça, Mozambique;
2. Barcelona Institute for Global Health (ISGlobal), Hospital Clínic-Universitat de Barcelona, Barcelona, Spain.
3. National Directorate of Health, Ministry of Health, Mozambique
4. National Institute of Health, Ministry of Health, Maputo, Mozambique
5. ICREA, Pg. Lluís Companys 23, 08010 Barcelona, Spain.
6. Pediatric Infectious Diseases Unit, Pediatrics Department, Hospital Sant Joan de Déu (University of Barcelona), Barcelona, Spain
7. Harvard T.H. Chan School of Public Health, Boston, MA, USA.

*Corresponding Author

E-mail: Beatriz.galatas@isglobal.org

¶ These authors contributed equally to this work

Emails of Authors

For peer review only

Beatriz.galatas@isglobal.org

Ariel.nhacolo@manhica.net

Helena.marti@isglobal.org

Humberto.munguambe@manhica.net

Laia.cirera@isglobal.org

Felimone.amone@manhica.net

Eusebio.macete@manhica.net

quique.bassat@isglobal.org

regina.rabinovich@isglobal.org

Alonsop@who.int

Pedro.aide@manhica.net

Francisco.saute@manhica.net

Charfudin.sacoor@manhica.net

Key words

Magude district, census, demographic surveillance site, population, health, malaria control tools, malaria elimination

Word count

5467

For peer review only - http://bmjopen.bmj.com/site/about/guidelines.xhtml

Abstract

(Word count: 302)

Objectives: A Demographic and Health Platform (DHP) was established in Magude in 2015, prior to the deployment of a project aiming to evaluate the feasibility of malaria elimination in southern Mozambique, named the Magude project. This platform aimed to inform the design, implementation and evaluation of the Magude project, through the identification of households, and population; and the collection of demographic, health and malaria information.

Setting: Magude is a rural district of Southern Mozambique which borders South Africa. It has nine peripheral health facilities and one referral health center with an inpatient ward.

Intervention: A baseline census enumerated and geolocated all the households, and their resident and non-resident members, collecting demographic and socio-economic information, and data on the coverage and usage of malaria control tools. Inpatient and outpatient data during the five years (2010-2014) before the survey were obtained from the district health authorities. The demographic platform was updated in 2016.

Results: The baseline census conducted in 2015 reported 48,448 (92.1%) residents and 4,133 (7.9%) non-residents, and 10,965 households. Magude's population is predominantly young, half of the population has no formal education and the main economic activities are agriculture and fishing. Houses are mainly built with traditional non-durable materials and have poor sanitation facilities. Between 2010 and 2014, malaria was the most common cause of all-age inpatient discharges (representing 20-40% of all discharges), followed by HIV (12-22%), and anemia (12-15%). In early 2015, all-age bed-net usage was between 21.8% and27.1% and the reported coverage of indoor residual spraying varied across the district between 30.7% and 79%.

For peer review only - http://bmjopen.bmj.com/site/about/guidelines.xhtml

Conclusion: This study revealed that Magude has limited socio-economic conditions, poor access to health care services and low coverage of malaria vector control interventions. Thus, Magude represented an area where it is most pressing to demonstrate the feasibility of malaria elimination.

For peer review only

Strengths and limitations of this study

- **Strengths:**
    - A Demographic and Health Platform (DHP) was established in Magude in 2015 to inform and accurately plan the interventions contemplated under the elimination project, given that the district's demographic data had not been updated since the 2007 national census.
    - A second census round was conducted one year later acknowledging that detailed population data collected at individual level at one point in time may miss individuals or households that can be captured after a later update.
    - The census rounds were planned in close collaboration with the community leaders and district authorities, and counted with intensified training of fieldworkers, and a strong component of field and data supervision by experienced demographers.
- **Limitations:**
    - Data collected through the DHP could have been affected by inaccuracies during data collection or entry, or by recall or desirability bias of census participants. Inpatient information was limited by the quality and accuracy of the data at the time it was collected, by disease-specific interventions or changes in diagnostics, referral or reporting practices. The data reported in the weekly epidemiological bulletin (BES) do not distinguish presumed from confirmed cases, neither by the diagnostic used (rapid diagnostic test or microscopy) or the place of detection (health facility or community), and are subject to RDTs stock-outs or reporting inaccuracies.

For peer review only - http://bmjopen.bmj.com/site/about/guidelines.xhtml

Background

Mozambique is one of the countries with the highest malaria burden in the world [1]. Malaria prevalence among children of 6 to 59 months of age is heterogeneous within the country ranging from high transmission intensity in the North (>50%) to less than 3% in the South, as reported in the latest malaria survey conducted in 2015 [2]. Lower prevalence in the Southern region may be related to the great progress against malaria in the past decades, partly as a result of the regional initiatives aiming for malaria elimination in the area. In line with the vision of a malaria free world established by the World Health Organization in its Global Technical Strategy for 2016-2030 [3], the National Malaria Control Program of Mozambique (NMCP) decided to redefine its strategic objectives in order to include the implementation of malaria elimination activities in the South. In this context, a malaria elimination project named the Magude Project was designed and evaluated in Magude district, Maputo province, by the Manhiça Health Research Center (CISM) and the Barcelona Institute of Global Health (ISGlobal), to assist the NMCP in adopting a malaria elimination strategy based on local evidence [4].

Prior to the initiation of the Magude project in 2015, there was limited and outdated information with regards to the number of individuals living in Magude, as well as to their demographic and socio-economic characteristics [5]. Thus, detailed information from the whole district was deemed crucial to inform the elimination strategies that had been planned for the following years in the district. The process of filling in this knowledge gap also aimed to identify and contact key leaders at provincial, district, and at community level, to inform and engage them in the activities prior to their deployment. In this context, a Demographic and Health Platform adapted from the Health and Demographic Surveillance System (HDSS) method was established in the district of Magude, in February of 2015

For peer review only - http://bmjopen.bmj.com/site/about/guidelines.xhtml

with the objective of providing reliable and updated demographic data to inform the project. This platform allowed to plan the activities and to provide a sampling frame to measure indicators in the community such as malaria prevalence, and to estimate the coverage of indoor residual spraying (IRS), coverage and usage of long-lasting insecticide treated nets (LLIN) and mass drug administration (MDA) campaigns. The DHP's permanent identification numbers were used to track individual's participation in each intervention of the Magude project longitudinally, thus allowing to identify and quantify potential challenges to the project, such as reasons for non-participation, or probable sources of imported infections. These data was also used to accurately measure prevalence at the community stratified by age groups and place of residence. Overall, these findings were crucial to the design of the Magude project, and offered robust evidence to guide malaria elimination strategies in Southern Mozambique.

This article presents the demographic, socio-economic and health characteristics of the population of Magude, as well as the coverage of malaria control interventions estimated through the baseline census conducted between February and June of 2015 It also presents the burden of disease in Magude during the five years (2010-2014) before the DHP, using the inpatient and outpatient data obtained from the district health authorities. A summary of the demographic profile of Magude after updating the census between August and September of 2016 is also provided Methods

Study area

The district of Magude is located in the North-Western part of Maputo Province and borders with the districts of Massingir, Chókwe and Bilene, from Gaza Province on the North and North-East; with the districts of Manhiça and Moamba, of Maputo Province in the East and South; and with the South African National Kruger Park, in the West (Figure 1A).

For peer review only

For peer review only - http://bmjopen.bmj.com/site/about/guidelines.xhtml

Magude was selected as an appropriate demonstration area for the malaria elimination project as it was expected to pose the types of challenges that the NMCP would face when implementing a malaria elimination campaign in the south of the country. This is, there were more than 13,000 malaria cases reported in the district in 2014, with the majority of cases observed between January and May, suggesting that the epidemiology of malaria in the district was representative of most endemic areas in the country with the typical seasonal pattern coinciding with the rainy season [6,7]. Additionally, the socio-economic and infrastructural limitations reported for Magude made it sufficiently representative of a rural district of Mozambique, while still at reach of CISM's facilities (located in Manhiça district), which facilitated the logistics, supervision and quality control processes. Finally, the population of Magude had had very limited exposure to research projects or targeted innovative malaria interventions prior to 2015, having only received the programmatic IRS, LLINs and child immunization campaigns conducted by the government. This allowed facing the challenges of working in an unexposed population that was not biased by previous activities.

Study design

A Demographic and Health Platform (DHP) was established in Magude in February 2015, by CISM, which was adapted from the health and demographic surveillance system (HDSS) previously established in Manhiça district by CISM [8]. A baseline census was conducted at that time (February to June) to identify and enumerate all neighborhoods, households and resident and non-resident individuals in these households. Geographic positioning system (GPS) coordinates were also captured for every household. was All individuals were assigned a permanent and unique identification number, linked to the household number where they were first enumerated. A second

For peer review only - http://bmjopen.bmj.com/site/about/guidelines.xhtml

census round was conducted between August and September of 2016, to review and update all information collected during the baseline census and record live births, deaths, and migrations that occurred between the two censuses. New households and new members were enumerated, and their information was collected to update the baseline databases.

This DHP defined a household as a structure or set of constructions where an individual or group of individuals live and share domestic activities and costs (such as eating and sleeping) and recognize one of them as their superior or chief, regardless of their kinship ties. Individuals were defined as residents if they had lived and slept in a household within the study area for a period of three months or more or intended to do so. Non-resident members of a household were defined as those who left the district or who had never lived in the study area for a period of three or more months, but who kept their roles in the households as head of household, husband, or other important breadwinners, and paid regular visits to their households in Magude. These individuals leave their households for specific reasons such work, studies, or imprisonment; and would otherwise reside in the referred household. This category excluded offspring or other members who had left the household to live in their own households outside the study area, irrespective of whether they kept visiting the reference household or not.

Data collection procedures

Baseline and updated data from the census rounds were collected through standardized questionnaires using Open Data Kit (https://opendatakit.org/) installed in Android tablets. Data were sent to a secure server at CISM using Wi-Fi. Information collected on households' socio-economic characteristics included building materials used for the main house, source of water and electricity, and household assets and livestock. At the individual level, the information collected included the

For peer review only

name, sex, date of birth, relation to the head of household, education, and occupation of all residents and non-residents of Magude. Information was also collected on malaria prevention tools, including the possession and use of LLINs, and whether the house had received IRS in the 12 months preceding the census. Individuals were also asked about history of fever during the preceding 30 days, and whether they had sought care at a health facility, community health worker, traditional healer, or none. The specific health facility where the individual sought care was also specified to delineate health facility catchment areas (HFCA) according to the community. Finally, information was collected on the migration and mobility patterns of all participants, to better characterize the mobility profile of the district, and offer potential information on the sources of new infections if transmission was eventually significantly reduced.

Administrative and geographical information was obtained directly from the district authorities and from the most recent district profile of Magude published in 2014 by the central government authority [7] and district statistics performed by the National Institute of Statistics [9,10]. We retrospectively collected monthly data on inpatient discharges from the referral health center of Magude Sede, as well as weekly outpatient malaria cases reported through the weekly epidemiological bulletin (Boletim Epidemiológico Semanal or "BES" in Portuguese) for the period of 2010-2014. The BES did not distinguish between presumed and confirmed cases, the diagnostic used (rapid diagnostic test or microscopy) or the location of detection (health facility or in the community).

Patient and Public Involvement

This study counted with the support and involvement of the community of Magude in all of its stages. During its design and planning, meetings were held with the district authorities and community leaders in order to inform them of the purpose of the DHP under the scope of the

For peer review only - http://bmjopen.bmj.com/site/about/guidelines.xhtml

Magude project, as well as to landscape the number of villages in Magude that had to be covered by the DHP. The study team worked closely with village chiefs when performing the household visits, which ensured that all district households were covered by the DHP. Reports were submitted to the administrative authorities to support vector control activities, among others. Preliminary findings were also communicated in meetings held with the community prior to the deployment of MDAs, where the findings obtained from the study – particularly focusing on malaria – where discussed in detail [11].

Data analysis

Data management and descriptive analyses were performed using R Software [12]. The percentage distribution of categories within any given variable were calculated considering the observations with missing values in the denominator. No inferential statistics (p-values or 95% confidence intervals) were calculated as the DHP covers the entire district, and therefore offers direct population parameters. Spatial visualization of the data collected on the field and obtained from web-based open source administrative data bases from DIVA-GIS [13] were performed using QGIS Software [14].

Household's socio-economic status was measured using a modified version of Oxford's Poverty and Human Development Initiative Multidimensional Poverty Index (MPI) [15], following the same methodology used by the INDEPTH group to estimate the poverty index of various HDSS sites throughout Africa [16]. In summary, this method categorizes households as "deprived" or "not deprived" based on six deprivation indicators: 1) lack of electricity, 2) lack or sharing of an improved sanitation facility; 3) lack of access to improved drinking water source, or source only available more than 30-minute walk, round trip; 4) sand floors in main houses; 5) dung, wood or charcoal used for cooking fuel; and 6) households that do not own a car nor truck and do not own more than one of

For peer review only

For peer review only - http://bmjopen.bmj.com/site/about/guidelines.xhtml

the following: radio, TV, telephone, bike, motorbike, or refrigerator. A deprivation indicator is generated as lowest depravation (0-2), moderate depravation (3-4), or highest depravation (5-6) [16].

Results

The geography of Magude district

The district of Magude has an area of 6,961 km^2 and is divided in five administrative posts, namely: Magude-sede, Motaze, Panjane, Mahele and Mapulanguene (Figure 1A). The vegetation of Magude is dominated by open forests and savannahs hosting animals such as impalas, warthogs, lions, buffalos and elephants. There is one permanent river (Incomati), which flows through the south-western region of the district and constitutes the main source of water in the area, and three intermittent rivers dependent on rainfall, called Massitonto, Uanétse and Mazimuchopes [7].

Demographic and socio-economic characteristics

The baseline census of 2015 registered 10,965 households and 52,802 individuals, of whom 48,448 (91.8%) were residents and 4,133 (7.8%) were non-residents. The district had a population density of 6.9 inhabitants per km^2, which varies by administrative post. The majority of the population (73%) lives in the Magude-Sede administrative post (the capital of the district); 14.1% in Motaze, 5.9% in Panjane, 3.7% in Mapulanguene, and 3.4% in Mahele (Table 1). The age and sex structure of the population is dominated by children and young people, and a sharp reduction of adults, particularly among males (Figure 1B).

For peer review only

For peer review only - http://bmjopen.bmj.com/site/about/guidelines.xhtml

The census update conducted between August and October of 2016 registered a population of 61,868 individuals. Of the population censed in 2015, 42,792 (81%) were found during the update round, 6,099 (11.5%) were reported to still be in Magude by a family member although field workers did not find them in other households and therefore were not able to confirm and complete the registration of migration. There were an additional 3,244 (6.1%) individuals censed in 2015 who were not found in 2016 and for whom there were no informants, but were still considered to be in Magude for this analysis. This update also recorded the death of 670 individuals censed in 2015, 1,687 live births and 721 immigrations since the baseline census. An additional 7,325 individuals who were missed in 2015 were censed during this update (Table 1).

The baseline census indicated that 62% of males and 63.1% of females above the age of 14 reported being married or in de-facto union; and 3% of married men practice polygamy. The illiteracy rate among those aged 6 and older in Magude is 51.9% among females and 47.4% among males. Approximately 37.2% of individuals reached 5th to 9th grade, and 9.5% have completed between 10th and 12th grade (Sup.Table 1). In 2015 Magude had 31 primary schools offering 1st to 5th grades; 33 offering 1st-7th grades; one secondary school offering 8th-12th grades; and one private higher-education training center. According to the national census performed in 2007, Xichangana is the main local language of the district and is the mother tongue of 92% of the population of Magude. However, 51% of the population also reports being able to speak Portuguese (the official language in Mozambique), which is the mother tongue of only 3.2%[7].

Occupations are unevenly distributed between males and females older than 18 years of age. The majority of the population (26.1% of males and 70.7% of females) relies on subsistence agriculture, fishing or work as cane cutters in the sugar plantations within Magude, or in Xinavane, in the nearby

For peer review only - http://bmjopen.bmj.com/site/about/guidelines.xhtml

district of Manhiça. Other occupations include being a salesperson (8.9% of men and 11% of women), doing construction work (22.1% of men, 0.7% of women), or making coal (11.5% of men and 3.1% of women). A small proportion of the population works as security guards, or work in the public sector, particularly as health professionals, teachers or in the army (Sup.Table 1).

Men represent 54.6% of heads of households in Magude. Only 0.4% of household heads are younger than 18 years of age and 5.6% are between the ages of 18-24, while 74.7% are between 25-64 years old, and 19.2% are older than 65 years of age (Table 2). Forty-three percent of heads of household are single, 35.4% are married or in de-facto union, 16.8% are widows and 4.3% separated. Fifty-nine percent of heads of households reported having no formal education, while 28.2% reported completing up to 5-9th grade, 8.7% up to 10th to 12th grade, and 0.4% reported having a university degree (Table 2).

Living conditions in Magude's households

Settlements in Magude are made of individual household compounds that are built in proximity to each other in the central areas of each administrative post, and more spread out in the rest of the district. The median household size is 5, although 1,171 households (10.7%) have only one resident, 40% of whom are older than 65 years old.

The majority of households are traditional round-shaped or rectangular-shaped huts constructed using cane (32.5%), cement (26%), mud bricks (21.6%), or reeds covered by adobe (15.6%) (Table 2). More than half of the households have traditional latrines (53.5%), 10.6% have improved latrines, while 33.8% of households do not have any form of sanitation facility. Flush toilets can only be found in 2.2% of the households. The primary lighting sources used in the households are paraffin (49.6%),

For peer review only - http://bmjopen.bmj.com/site/about/guidelines.xhtml

electricity (30.8%), candles (9.9%) and solar panels (4.6%). The majority of households cook using wood logs (59.3%). Water is mainly obtained from pumps (34.3%) or piped water (20.5%), although a quarter of the population collects water directly from the river (24.9%) or from open wells near the river (10.5%) (Table 2).

With regards to household assets, 68.5% of households own at least one mobile phone, 36.6% own a radio, 32.7% have a television (TV), while 16.8% and 12.2% of households have a fridge and a freezer, respectively. Almost a quarter of households reported having bicycles (23.7%), while 10.8% reported owning cars, 5.3% motorbikes, and 0.7% trucks (Sup.Figure 1).

Households in Magude had a median number of 4 depravations. Twenty-five percent of the households were in the lowest depravation category, with 0-2 deprivation indicators; while 55..3% fell in the 3-4 depravation group and 22.4% in the 5-6 depravation group. This distribution was unevenly observed among the different administrative posts. Magude Sede had the highest proportion of households in the lowest depravation group (32%), while Mapulanguene had the highest proportion of households in the moderate depravation category (70.8%). Panjane and Mahele were the administrative posts with the highest proportion of highly deprived households (Sup. Table 2).

For peer review only

Mobility patterns

The baseline census shouwed that 5% of residents reported having spent the night before the census outside of Magude (6.4% of males and 5% of females). Of these, 43% were younger than 14 years old, 25.1% were 15-29, 18.1% were 30-44, 10.3% were 45-64 and 3.4% were older than 65 years old (Sup.Table 3).

For peer review only - http://bmjopen.bmj.com/site/about/guidelines.xhtml

Almost half of the population (43% of males and 46.9% of females) did not report frequently travelling outside of Magude during the baseline census. Among the 54.4% who did, the places most frequently visited included Maputo City (55.3% of males, 58.3% of females), South Africa (23.1% of males and 16.8% of females), Gaza Province (12.2% of males and 15.9% of females), Inhambane Province (4.3% males and 2.6% females), and other Northern provinces within Mozambique (5.2% of males and 6.3% of females). Younger individuals (less than 15 years old) reported travelling the most to all provinces within the country, followed by those between the ages of 15-30; while 15 to 45-year-olds are more likely to travel to South Africa. Individuals older than 45 reported travelling more to provinces northern of Maputo province, as well as abroad (Sup.Figure 2).

Health and malaria information

The district has 10 functional health facilities– one with an inpatient and maternity ward (Type I) and nine HFs only with maternity wards (Type II). Eight of the ten HFs were active in 2015, and 2 more Type II HFs were added in 2016 and 2017. The referral HF, located in the Magude-Sede administrative post, has 57 beds and three active medical doctors (0.06 doctors per 1,000 inhabitants). The other 9 Type II health facilities have 37 beds in total. The whole district has only three medical doctors, 15 general nurses and 19 maternal and child health nurses (Figure 1A). Only 3 HFs have access to piped water, 4 have access to public network electricity, and 5 rely only on solar panels. All health facilities are located on a local main road to facilitate access, and the median Euclidian distance from households to the nearest health facility is 2.7 km (interquartile range [IQR] 1.4–7.9 km), although households are as close as 15 m or as far as 38.8 km. There is only one ambulance in Magude, which is used for transferring patients within the district, but also to the hospitals in Xinavane, Manhiça, and Maputo.

For peer review only

For peer review only - http://bmjopen.bmj.com/site/about/guidelines.xhtml

The health system in Mozambique includes Community Health Workers (or APEs, from its acronym in Portuguese) who are trained by the Ministry of Health to offer primary health services in areas with poor access to HFs. They provide diagnosis and treatment for malaria, diarrhea, and pneumonia, and to refer patients with signs of sickness requiring higher medical attention [17]. In Magude there are 27 APEs distributed throughout the district (Figure 1A). The average distance between households and the nearest APE is approximately 6.3 km (median of 4.4 km, IQR 2.8–5.4 km).

All HFs and APEs in Magude are equipped to diagnose malaria through Rapid Diagnostic Tests (RDTs) –light microscopy is only available in the Magude Sede Type I HF. They also offer treatment to all positive cases with Artemether-Lumefantrine, the first line treatment in Mozambique. Intermittent Preventive Treatment in pregnancy (IPTp) using Sulfadoxine-pyrimethamine is also offered in all HFs of the district. In May of 2014 the NMCP conducted an LLIN universal distribution campaign, with 35,432 bed nets distributed in Magude district. This was followed by a focal IRS campaign between October and December of 2014 using the insecticides deltamethrine and DDT, which was only deployed in the Motaze administrative post, where the malaria burden was highest.

Malaria has traditionally been the first cause of disease in the district, responsible for approximately 53% of all consultations reported in 2003 [7]. According to the inpatient discharge data available from January of 2010 to December of 2014, between 20-40% of all-age discharges in the inpatient department were due to malaria, followed by HIV (12-22%), anemia (12-15%), pneumonia (6-12%), and diarrhea (3-8%), varying by month and year (Fig 2A). The weekly number of malaria cases reported through the BES in Magude between 2010 and 2014 follows a seasonal pattern with a peak between December and May and a reduction of cases during the dry season (Fig 2B). According to

For peer review only

For peer review only - http://bmjopen.bmj.com/site/about/guidelines.xhtml

363 BES data, there were 293 cases per 1000 populationreported during the transmission year of July 2011 to June 2012, 247 per 1000 between July 2012 and June 2013, 252 cases per 1000 between July 2013 and June 2014, and 110 cases per 1,000 between July 2014 and June 2015. [7].

367 The prevalence of history of fever during the 30 days prior to the baseline census was 12.1% in children under the age of 5, 7.7% among 5-14 year-olds, and 11.6% in individuals older than 15 years. Almost all individuals reported going to a health facility as their primary source of health care (>99%), while a small proportion mentioned seeking care first from traditional healers or community health workers. The proportion of individuals who reported sleeping under a bed net the night before was 27.1% among children under the age of 5, 21.8% in 5-14 year-olds, and 27% among those > 15 years of age (Table 3). The universal bed-net coverage (i.e. one net for every two individuals of a household) in 2015 was 52.7% in the administrative post of Magude-Sede, 59.9% in Motaze, 44.1% in Panjane, 52.7% in Mahele and 52.1% in Mapulanguene. Finally, the reported coverage of IRS during the previous 12 months was 49% in Magude-Sede, 79% in Motaze, 41.1% in Panjane, 61% in Mahele, and 30.7% in Mapulanguene (Table 2 and Sup.Table 4).

378 Discussion

379 Overall, the Demographic and Health Platform established in Magude offered detailed insight on the baseline socio-demographic profile of Magude district prior to the Magude project. It revealed that the number of residents in Magude in 2015 (52,804 individuals and 10,965 households) was similar to the number reported by the 2007 national census conducted by the National Institute of Statistics (INE) (53,229 individuals and 11,408 households) [9] but did not coincide with the projected population for Magude in 2015 estimated by the INE to be 62,000) [10], which is not surprising

For peer review only

acknowledging the limitations of population projections. The census update conducted in 2016 identified individuals who had been missed by the baseline census, and recorded the births, deaths, and in-migrations since baseline. As a result, the population of Magude in 2016 assuming that those censed in 2015 who were not found in 2016 were still living in the district, was 61,868 individuals, which is similar to the projection by INE for this year (62,924).

The usefulness of a Demographic and Health Surveillance System in demographic and bio-medical research in African countries has been previously described [18]. Having a precise source of population data is crucial for the correct estimation of the risk of disease and coverage of health indicators in a population, as well as for planning purposes of public health interventions [19]. This platform has shown that detailed population data collected at individual level at one point in time still misses a number of individuals, who can be captured after a later update. However, all the census rounds faced the challenge of finding individuals at home, or informants of unavailable individuals, to identify whether a member lives in the area despite not being found during a specific census round. To identify or collect information about absent individuals, field supervisors attempt to arrange interviews with them at their places of work, or interview their family members at their households during weekends. The same phenomenon is expected to take place when implementing malaria interventions in the community (such as rounds of IRS or of mass drug administrations), which generally challenges the estimation of intervention coverage.

Household-level data indicates that the households in Magude are typical of a rural area with suboptimal conditions regarding access to water, electricity, and sanitation. The majority of houses were relatively homogeneous, built with non-durable materials, such as cane and adobe, and did not

For peer review only - http://bmjopen.bmj.com/site/about/guidelines.xhtml

have access to clean water, conditions indicative of a low socio-economic status [20]. The age and sex composition of Magude's population is typical of rural Mozambique and sub-Saharan Africa, composed primarily by children and young individuals and a decreasing proportion of adults as age increases [21]. Another important aspect of the structure of the population of Magude is the lower sex ratio (number of males in relation to that of females) among young and middle-aged adults, which has been reported, in the neighbouring district of Manhiça, to be related to male labour migration to Maputo city or South Africa and higher male mortality [21].

A large proportion of the heads of households and of the overall population of Magude reported not having received a formal education, a major risk factor for health outcomes. Additionally, most individuals reported having occupations related to agriculture or fishing, which are usually carried out early in the morning until around noon. These aspects should be taken into consideration when planning mobilization campaigns and community-based interventions, as visits might have to be conducted at certain times of day and/or venues outside the households to be able to find these individuals.

Magude is also subject to significant mobility and migration, as more than half of the population reported travelling frequently outside of the district. The destinations most reported by travelers living in Magude were Maputo and South Africa, followed by Gaza and Inhambane provinces. This information is useful to evaluate the risk of malaria importation from other areas due to travel or migration [22]. Maputo city and South Africa are areas with lower malaria burden than Magude [23]; however, the districts surrounding Magude (Manhiça, Moamba), the provinces of Gaza, Inhambane and the rest of Mozambique have higher malaria prevalence estimates than Magude district [2], and

For peer review only

For peer review only - http://bmjopen.bmj.com/site/about/guidelines.xhtml

could be a source of imported infections [24]. These places are commonly visited by all age groups, whereas the lower-endemic areas are mainly visited by <30 year-olds. This age pattern of migration is typical of the rural areas of Southern Mozambique, where migration rates are higher among individuals aged 20 to 45 years and their children, and decrease with increasing age when individuals establish as permanent residents in an area.

The population of Magude has access to ten health facilities, one of them with an inpatient ward with 57 beds. Thus there are 11.8 beds per 10,000 population, a ratio that is higher than the national level (7 beds per 10,000 in Mozambique) [25]. While most respondents indicated seeking health care primarily from formal health facilities, the majority of the population is scattered throughout the district as far as 38 kms away from a HF and it is difficult to access health facilities or APEs due to limited roads and lack of transportation. This has implications for the delivery of public health interventions, especially for those that rely on the passive detection of cases to control transmission in the community.

Data between 2010 and 2014 from the outpatient and inpatient department of the Magude Sede health center indicated that malaria was the main cause of hospitalization and later discharge with a clear seasonal pattern. The population of Magude is also burdened by HIV, anemia, pneumonia, and diarrhea, similar to its neighboring districts of Manhiça and Chokwé [26–33]. Inpatient information was retrospectively collected, and thus, its quality and accuracy depends on the data at the time at which it was collected. It may also be biased by disease-specific interventions or changes in diagnostics, referral protocols of severe cases to other districts, or reporting practices. Additionally, cases reported by BES are difficult to interpret given their lack of disaggregation in presumed or confirmed, diagnostic

For peer review only - http://bmjopen.bmj.com/site/about/guidelines.xhtml

454 tool used or place of detection of the case. Thus, they are subject to changes in RDT use and stock-outs, in care seeking behavior to HFs or APEs, or reporting inaccuracies. In fact, cases reported by the APEs are usually excluded by those who report in BES, and neither the total number of fevers nor the number of individuals tested are reported. Thus, a stronger surveillance system than BES was thought to be required, and consequently established in Magude in 2015,to fully capture the clinical malaria profile of a district aiming to eliminate malaria [4,34].

The age-specific self-reported history of fever in the past 30 days (7.7%-12.1%) were slightly lower than the average percentage of fevers reported in the past 2 weeks in Maputo Province, which was 15% according to the most recent malaria indicator survey [2]. Approximately half of the households in Magude reported owning one bed net for every two individuals (universal coverage). However, only a fourth of the population reported sleeping under a bed net the previous night. These findings mirror the estimates reported for Maputo Province in 2011 by the Demographic Health Survey, which found a reported bed net usage rate of 27% in Maputo Province [35]. This information is indicative of an area where bed net owners do not necessarily use the net as a preventive tool against malaria during the rainy season, despite this being a prevalent disease in the district. Other demographic and socio-economic risk factors that have been associated with LLIN use, such as households with more children, an undereducated household head, or households far away from the health facilities, might explain the low bed net usage in Magude [36–38].

The reported IRS coverage in the area of Motaze was higher, which corresponds with the focal IRS campaign conducted by the NMCP that took place only there in September of 2014. The coverage reported in other areas was probably the consequence of recall bias, as a district-wide IRS campaign

For peer review only

For peer review only - http://bmjopen.bmj.com/site/about/guidelines.xhtml

took place in 2013. Overall, the coverage reported for LLIN and IRS throughout the district was below the World Health Organization recommended coverage of >80% [39], leaving a significant proportion of the population unprotected by any of the standard preventative measures. The identification of gaps in such vector control coverages called for a strong community engagement campaign focused on the use of the bed nets and participation in the yearly rounds of IRS.

Conclusion

Through the establishment of a Demographic and Health Platform in Magude in 2015, which was updated in 2016, and a baseline assessment of the relevant health indicators, it was possible to fully characterize a district where malaria elimination interventions were to be deployed and evaluated. Magude represents a typical rural district of Mozambique characterized by limited social and economic infrastructures, which had to be considered for the design and operationalization of the community-based interventions. This study also revealed a low education level among a large proportion of the population and identified agriculture as the main economic activity in the district. These socio-demographic characteristics suggest that a strong community engagement would be necessary for the implementation of a malaria elimination project. Half of the population of Magude reported travelling outside of the district to areas of high and low malaria transmission intensity, showing that malaria importation will likely be a source of continuous transmission even if interruption of local transmission is achieved. Also, the poor access to health care services, as well as to core malaria vector control interventions such as IRS and bed nets distributed by the NMCP, indicated that these aspects needed to be integrated within the malaria elimination program planned for the district, in order to maximize the impact of the interventions aiming to interrupt transmission. Overall, this district represents the reality of the majority of malaria endemic areas in Sub-Saharan Africa, where

For peer review only - http://bmjopen.bmj.com/site/about/guidelines.xhtml

elimination is most needed, and where it is most pressing to demonstrate the feasibility of elimination strategies.

For peer review only - http://bmjopen.bmj.com/site/about/guidelines.xhtml

Abbreviations

APEs – Agentes Polivalentes Elementais (Community Health Workers)

BES – Boletim Epidemiológico Semanal (Weekly Epidemiological Bulletin)

CISM – Manhiça Health Research Center

DHP – Demographic and Health Platform

GPS – Global Positioning System

HDSS – Health and Demographic Surveillance System

HF – Health Facility

HIV – Human Immunodeficiency virus

INE – Instituto Nacional de Estatística (National Institute of Statistics)

IPTp – Intermittent Preventive Treatment in pregnancy

IRS – indoor residual spraying

ISGlobal – Barcelona Institute of Global Health

LLINs – Long-Lasting Insecticide treated Nets

MDAs – Mass Drug Administration

NMCP - National Malaria Control Program of Mozambique

RDTs – Rapid Diagnostic Tests

TV – Television

For peer review only

For peer review only - http://bmjopen.bmj.com/site/about/guidelines.xhtml

Declarations

Acknowledgements

We would like to thank the community of Magude for participating in this study, and the team of field workers who have collected the data presented here. We would also like to acknowledge the Administrative and District Health Authorities of Magude for their collaboration and for providing some of the information also included in this article. We thank everyone who supported this study directly or indirectly through fieldwork, or analysis support.

Ethics approval and consent to participate

The Demographic and Health Platform protocol, consent forms and questionnaires were approved by the CISM internal ethics committee and written consent from the health authorities was also sought prior to its implementation. Meetings were held with community leaders and with general members of the community of Magude to inform them about the DHP operations and the malaria elimination project as a whole. A written informed consent was obtained from all household heads to record household-level information, as well as from all individuals providing individual information. Informed consents for children under the age of 18 years were sought from their parents or primary caretakers. The collection of district health surveillance data was conducted under the protocol that aimed to evaluate the impact of the *Magude project* on malaria transmission, which was approved by CISM's internal ethical committee, the Ethics Committee of the Hospital Clínic of Barcelona, and the Ministry of Health National Bioethics Committee of Mozambique (IRB00002657).

Competing Interests

The authors declare that they have no competing interests

For peer review only

Funding

Funding was provided by the Bill and Melinda Gates Foundation and the Fundación "la Caixa" Partnership for the Elimination of Malaria in Southern Mozambique (OPP1115265). Q. B. is an ICREA (Institut Catal. de la Recerca i Estudis Avan.ats; Catalan Government) Research Professor. ISGlobal is a member of the CERCA Programme, Generalitat de Catalunya.

Author's Contributions

BG: participated in the study design and fieldwork, supported in the data cleaning and data analysis process, and wrote the draft of this article

AN: participated in the study design, in study analyses, in the interpretation of results and writing of this article

HMS: cleaned and analyzed the data, and contributed to the writing of this article

HM: led the implementation of field activities and data collection process, and participated in the interpretation of results

EJ: participated in the study design and implementation of field activities and data collection.

CG: participated in the interpretation of results and writing of this article

LC: participated in the study design, interpretation of results and writing of this article

FA: designed the data collection tools, supported the implementation of field activities and data cleaning.

EM: participated in the study design, and interpretation of results

QB: participated in the study design, interpretation of results and writing of this article

NRR: participated in the interpretation of results and writing of this article

PLA: participated in the study design, interpretation of results and writing of this article

For peer review only

For peer review only - http://bmjopen.bmj.com/site/about/guidelines.xhtml

PA: participated in the study design, field implementation interpretation of results and writing of this article

FS: participated in the study design, supervised field activities, interpretation of results and writing of this article

CS: conceived the study, participated in interpretation of results and writing of this article

Availability of data

The datasets collected as part of the Demographic Health Platform and analysed during the current study are available from the corresponding author upon written request.

The data presented in figures 2a and 2b belongs to the Ministry of Health of Mozambique and is considered as third-party data which can be accessed by contacting the following individuals appointed by the Ministry of health:

- To gain access to Inpatient Department data collected by the Ministry of health please contact Dr Baltazar Candrinho, Head of NMCP Mozambique, phone number:+258828665730 or e-mail: candrinhobaltazar@gmail.com
- To gain access to the data collected through the Boletim Epidemiologico Semanal (BES) please contact Dr Lorna Gujral, Department of Epidemiology/MOH/Mozambique, phone number: +25882 3250800 or e-mail: lornita15.lgg@gmail.com

For peer review only

584 # References

585

586 1. World Health Organization. World malaria report 2018. :210.

587 2. Ministério da Saúde (MISAU), Instituto Nacional de Estatística (INE), ICF Internacional. Inquérito
588 de Indicadores de Imunização, Malária e HIV/SIDA em Moçambique 2015. Maputo. Moçambique.
589 Rockville, Maryland, EUA: INS, INE e ICF International; 2015.

590 3. World Health Organization, others. A framework for malaria elimination. 2017 [cited 2017 Mar 28];
591 Available from: http://apps.who.int/iris/handle/10665/254761

592 4. Aide P, Candrinho B, Galatas B, Munguambe K, Guinovart C, Luis F, et al. Setting the scene and
593 generating evidence for malaria elimination in Southern Mozambique. Malar J. 2019;18:190.

594 5. Instituto Nacional de Estatistica. Relatório Final do Inquérito ao Orçamento Familiar- IOF 2014/15
595 [Internet]. 2015. Available from: http://www.ine.gov.mz/operacoes-estatisticas/inqueritos/inquerito-
596 sobre-orcamento-familiar

597 6. Maharaj R, Moonasar D, Baltazar C, Kunene S, Morris N. Sustaining control: lessons from the
598 Lubombo spatial development initiative in southern Africa. Malar J [Internet]. 2016 [cited 2016 Oct
599 17];15. Available from: http://malariajournal.biomedcentral.com/articles/10.1186/s12936-016-1453-9

600 7. Ministerio de Administração Estatal. Perfil do distrito de Magude, Provincia de Maputo (2014).
601 Ministerio de Administração Estatal; 2014.

602 8. Alonso P, Saúte F, Aponte J, Gómez-Olivé F, Nhacolo A, Thompson R, et al. Manhiça demographic
603 surveillance system, Mozambique. Popul Health Surviv INDEPTH Sites. 2002;1:295–308.

604 9. Instituto Nacional de Estatística. Estatísticas do Distrito de Magude. 2010.

For peer review only - http://bmjopen.bmj.com/site/about/guidelines.xhtml

605 10. Instituto Nacional de Estatistica. População Projectada por distritos, Maputo Província 2007_2040.xls — Instituto Nacional de Estatistica [Internet]. [cited 2018 May 29]. Available from: http://www.ine.gov.mz/estatisticas/estatisticas-demograficas-e-indicadores-sociais/projeccoes-da-populacao/populacao-projectada-por-distritos-maputo-provincia-2007_2040.xls/view

609 11. Portugaliza HP, Galatas B, Nhantumbo H, Djive H, Murato I, Saúte F, et al. Examining community perceptions of malaria to inform elimination efforts in Southern Mozambique: a qualitative study. Malar J [Internet]. 2019 [cited 2019 Aug 21];18. Available from: https://malariajournal.biomedcentral.com/articles/10.1186/s12936-019-2867-y

613 12. R, Foundation for Statistical Computing. R: A language and environment for statistical computing [Internet]. Vienna, Austria; Available from: http://www.R-project.org/

615 13. Hijmans R, Cruz M, Rojas E, Guarino L. DIVA-GIS, version 1.4. A geographic information system for the management and analysis of genetic resources data. 2001;

617 14. QGIS Development Team (2017) [Internet]. Open Source Geospatial Foundation Project; Available from: http://qgis.osgeo.org

619 15. Alkire S, Santos ME. Acute Multidimensional Poverty: A New Index for Developing Countries. SSRN Electron J [Internet]. 2010 [cited 2020 Feb 8]; Available from: http://www.ssrn.com/abstract=1815243

622 16. Coates MM, Kamanda M, Kintu A, Arikpo I, Chauque A, Mengesha MM, et al. A comparison of all-cause and cause-specific mortality by household socioeconomic status across seven INDEPTH network health and demographic surveillance systems in sub-Saharan Africa. Glob Health Action. 2019;12:1608013.

For peer review only

For peer review only - http://bmjopen.bmj.com/site/about/guidelines.xhtml

17. Ndima SD, Sidat M, Give C, Ormel H, Kok MC, Taegtmeyer M. Supervision of community health workers in Mozambique: a qualitative study of factors influencing motivation and programme implementation. Hum Resour Health. 2015;13:63.

18. Bocquier P, Sankoh O, Byass P. Are health and demographic surveillance system estimates sufficiently generalisable? Glob Health Action. 2017;10:1356621.

19. Tatem AJ, Campiz N, Gething PW, Snow RW, Linard C. The effects of spatial population dataset choice on estimates of population at risk of disease. Popul Health Metr. 2011;9:4.

20. Pons-Duran C, González R, Quintó L, Munguambe K, Tallada J, Naniche D, et al. Association between HIV infection and socio-economic status: evidence from a semirural area of southern Mozambique. Trop Med Int Health. 2016;21:1513–21.

21. Nhacolo AQ, Nhalungo DA, Sacoor CN, Aponte JJ, Thompson R, Alonso P. Levels and trends of demographic indices in southern rural Mozambique: evidence from demographic surveillance in Manhiça district. BMC Public Health [Internet]. 2006 [cited 2017 Jun 6];6. Available from: http://bmcpublichealth.biomedcentral.com/articles/10.1186/1471-2458-6-291

22. Ruktanonchai NW, Bhavnani D, Sorichetta A, Bengtsson L, Carter KH, Córdoba RC, et al. Census-derived migration data as a tool for informing malaria elimination policy. Malar J. 2016;15:273.

23. Moonasar D, Maharaj R, Kunene S, Candrinho B, Saute F, Ntshalintshali N, et al. Towards malaria elimination in the MOSASWA (Mozambique, South Africa and Swaziland) region. Malar J [Internet]. 2016 [cited 2016 Aug 25];15. Available from: http://malariajournal.biomedcentral.com/articles/10.1186/s12936-016-1470-8

For peer review only

For peer review only - http://bmjopen.bmj.com/site/about/guidelines.xhtml

647 24. Ruktanonchai NW, Smith DL, De Leenheer P. Parasite sources and sinks in a patched Ross–Macdonald malaria model with human and mosquito movement: Implications for control. Math Biosci. 2016;279:90–101.

650 25. World Health Organization. GHO | By country | Mozambique - statistics summary (2002 - present) [Internet]. WHO. [cited 2018 May 29]. Available from: http://apps.who.int/gho/data/node.country.country-MOZ?lang=en

653 26. González R, Augusto OJ, Munguambe K, Pierrat C, Pedro EN, Sacoor C, et al. HIV Incidence and Spatial Clustering in a Rural Area of Southern Mozambique. PloS One. 2015;10:e0132053.

655 27. Feldblum PJ, Enosse S, Dubé K, Arnaldo P, Muluana C, Banze R, et al. HIV prevalence and incidence in a cohort of women at higher risk for HIV acquisition in Chókwè, southern Mozambique. PloS One. 2014;9:e97547.

658 28. Deus N de, João E, Cuamba A, Cassocera M, Luís L, Acácio S, et al. Epidemiology of Rotavirus Infection in Children from a Rural and Urban Area, in Maputo, Southern Mozambique, before Vaccine Introduction. J Trop Pediatr. 2018;64:141–5.

661 29. Nhampossa T, Mandomando I, Acacio S, Quintó L, Vubil D, Ruiz J, et al. Diarrheal Disease in Rural Mozambique: Burden, Risk Factors and Etiology of Diarrheal Disease among Children Aged 0-59 Months Seeking Care at Health Facilities. PloS One. 2015;10:e0119824.

664 30. Aguilar R, Moraleda C, Quintó L, Renom M, Mussacate L, Macete E, et al. Challenges in the diagnosis of iron deficiency in children exposed to high prevalence of infections. PloS One. 2012;7:e50584.

667 31. Garcia-Basteiro AL, Miranda Ribeiro R, Brew J, Sacoor C, Valencia S, Bulo H, et al. Tuberculosis on the rise in southern Mozambique (1997-2012). Eur Respir J. 2017;49.

For peer review only

For peer review only - http://bmjopen.bmj.com/site/about/guidelines.xhtml

32. Lanaspa M, O'Callaghan-Gordo C, Machevo S, Madrid L, Nhampossa T, Acácio S, et al. High prevalence of Pneumocystis jirovecii pneumonia among Mozambican children <5 years of age admitted to hospital with clinical severe pneumonia. Clin Microbiol Infect Off Publ Eur Soc Clin Microbiol Infect Dis. 2015;21:1018.e9-1018.e15.

33. Roca A, Sigauque B, Quinto L, Morais L, Berenguera A, Corachan M, et al. Estimating the vaccine-preventable burden of hospitalized pneumonia among young Mozambican children. Vaccine. 2010;28:4851–7.

34. Barclay VC, Smith RA, Findeis JL. Surveillance considerations for malaria elimination. Malar J. 2012;11:304.

35. Ministerio da Saude (MISAU), Instituto Nacional de Estatística (INE) e ICF International (ICFI). Moçambique Inquérito Demográfico e de Saúde 2011. Calverton Md USA MISAU INE E ICFI. 2011;

36. Pinchoff J, Hamapumbu H, Kobayashi T, Simubali L, Stevenson JC, Norris DE, et al. Factors Associated with Sustained Use of Long-Lasting Insecticide-Treated Nets Following a Reduction in Malaria Transmission in Southern Zambia. Am J Trop Med Hyg. 2015;93:954–60.

37. García-Basteiro AL, Schwabe C, Aragon C, Baltazar G, Rehman AM, Matias A, et al. Determinants of bed net use in children under five and household bed net ownership on Bioko Island, Equatorial Guinea. Malar J. 2011;10:179.

38. Atieli HE, Zhou G, Afrane Y, Lee M-C, Mwanzo I, Githeko AK, et al. Insecticide-treated net (ITN) ownership, usage, and malaria transmission in the highlands of western Kenya. Parasit Vectors. 2011;4:113.

For peer review only - http://bmjopen.bmj.com/site/about/guidelines.xhtml

39. World Health Organization, World Health Organization, Global Malaria Programme. Global technical strategy for malaria, 2016-2030 [Internet]. 2015 [cited 2018 Mar 5]. Available from: http://apps.who.int/iris/bitstream/10665/176712/1/9789241564991_eng.pdf?ua=1

For peer review only

For peer review only - http://bmjopen.bmj.com/site/about/guidelines.xhtml

Tables

Table 1: Summary of Magude Population in 2015 and 2016.

	District Level		Magude Sede		Motaze		Panjane		Mahele		Mapulan-guene	
	N	%	N	%	N	%	N	%	N	%	N	%
**Households 2015**	10965		8011	73.1	1471	13.4	627	5.7	377	3.4	479	4.4
**Households 2016**	11960		8520	71.3	1441	12.1	645	5.4	803	6.7	547	4.6
**Population 2015**	52802		38534	73.0	7421	14.1	3122	5.9	1784	3.4	1934	3.7
Residents	48448	91.8*	35346	73.0	6605	13.6	2930	6.0	1695	3.5	1868	3.9
Non-residents	4133	7.8*	2970	71.9	816	19.7	191	4.6	89	2.2	66	1.6
Unclassified	221	0.4*	218	99.5	0	-	1	0.5	0	-	0	-
**Population 2016**	61868		44203	71.4	8149	13.2	3576	5.8	3499	5.7	2404	3.8
Residents	56943 ±	92.1*	40623	71.4	7275	12.8	3361	5.9	3329	5.8	2322	4.1
Non-residents	4535 ø	7.3*	3266	72.0	842	18.6	206	4.5	143	3.2	76	1.7
Unclassified	390 §	0.6*	314	80.9	32	8.2	9	2.3	27	7.0	6	1.5
**Demographic Events 2015-16**	3078		2204	71.6	390	12.7	186	6.0	168	5.5	129	4.2
Births	1687	54.8	1203	71.4	220	13.0	92	5.5	91	5.4	80	4.7
Immigrations	721	23.4	521	72.3	60	8.3	51	7.1	60	8.3	29	4.0
Deaths	670	21.8	480	71.6	110	16.4	43	6.4	17	2.5	20	3.0

For peer review only

For peer review only - http://bmjopen.bmj.com/site/about/guidelines.xhtml

* Column %

± 56,943 = 48,448 (censed in 2015) – 616 (deaths) + 1,670 (births) + 1,235 (immigrations) + 6,206 (censed in 2016)

ø 4,535 = 4,133– 42 (deaths) + 12 (births) + 64 (immigrations) + 368 (censed in 2016)

§ 390 = 221 – 8 (deaths) + 5 (births) + 21 (immigrations) + 151 (censed in 2016)

For peer review only

For peer review only - http://bmjopen.bmj.com/site/about/guidelines.xhtml

Table 2: Socio-demographic characteristics of heads of households, and household characteristics (2015).

Characteristics of heads of households*		N	%
Sex	Males	5051	54.6
	Females	4206	45.4
Age group (years)	< 18	40	0.4
	18 – 24	520	5.6
	25 – 64	6919	74.7
	> = 65	1777	19.2
Education level	No formal education	5460	59.0
	5-7th grade	2611	28.2
	8 -12th grade	807	8.7
	University	37	0.4
	Missing Information	342	3.7
Marital status	Single	4007	43.3
	Married or de-facto union	3275	35.4
	Divorced	16	0.2
	Separated	393	4.3
	Widow	1554	16.8
Residents of Magude		9257	84.4
**Households characteristics**			
Average household size**		5 [3-7]	
Lone-resident households		1171	10.7
Wall material of the main building	Cane	3506	32.5
	Cement	2807	26.0
	Mud bricks	2338	21.6
	Adobe	1703	15.8
	Zinc plates	319	3.0
	Wood	128	1.2
Type of toilet	Traditional latrine	5774	53.5
	Improved latrine	1144	10.6
	WC connected to septic tank	233	2.2
	No latrine	3650	33.8
Main source of lighting energy	Paraffin	5362	49.6
	Electricity	3321	30.8
	Candles	1074	9.9
	Solar panels	502	4.6

peer review only

For peer review only - http://bmjopen.bmj.com/site/about/guidelines.xhtml

	Other	539	5.0
Kitchen Fuel	Wood logs	6401	59.3
	Coal	740	6.9
	Gas	46	0.4
	Electricity	65	0.6
	Paraffin	8	0.1
	Missing Information	3541	32,8
Primary source of drinking water	Water pumps	3700	34.3
	Directly from the river	2691	24.9
	Piped water	2218	20.5
	Open well near the river	1138	10.5
	Other	1054	9.8
**Vector control tools**			
% of households with >1 ITN		8906	81.2
Universal ITN coverage (one net per two household members)		5690	53.2
IRS in past 12 months	Yes	5722	52.2
	No	4719	43.0
	Unknown	524	4.8

* or subhead (if head does not live in the household)

**Median [Interquartile Range]

Table 3: Individual-level health and malaria prevention indicators in Magude district 2015).

Age group in Years	< 5		5-14		>15	
	N	%	N	%	N	%
Had fever in preceding 30 days						
Yes	1043	12.1	1128	7.7	2931	11.6
No	7492	87.2	13325	91.2	21977	87.1
Unknown	55	0.6	154	1.1	319	1.3
Primary source of health care						
Health Facility	8552	99.6	14548	99.6	25075	99.4
Traditional Healer	13	0.2	27	0.2	61	0.2
Community Health Worker	22	0.3	32	0.2	66	0.3

For peer review only - http://bmjopen.bmj.com/site/about/guidelines.xhtml

Slept under a bed net the preceding night						
Yes	2330	27.1	3179	21.8	6799	27.0
No	6260	72.9	11428	78.2	18428	73.0

For peer review only

Figure Titles and Legends

Fig 1: Map of Magude district (2015).

A) Administrative and permanent river shape files were obtained from DIVA-GIS ((http://www.diva-gis.org/Data), and confirmed with Magude's key informants. GPS positions of households, health facilities and community health workers obtained directly from the field and mapped using QGIS. B) Population pyramid of Magude district (2015) showing the proportion of five—year age and sex groups out of the total population.

Fig 2: Health profile of Magude district prior to the baseline census (2010-2014).

A) Most common diseases leading to all-age hospitalization and later discharge in Magude reported by the District Health Authorities between 2010-2014. B) Weekly number of outpatient malaria cases observed in Magude between 2010 and 2014 reported through the Weekly Epidemiological Bulletin (BES) and monthly rainfall data obtained from the Climate Hazards Group InfraRed Precipitation with Station data (CHIRPS).

For peer review only - http://bmjopen.bmj.com/site/about/guidelines.xhtml

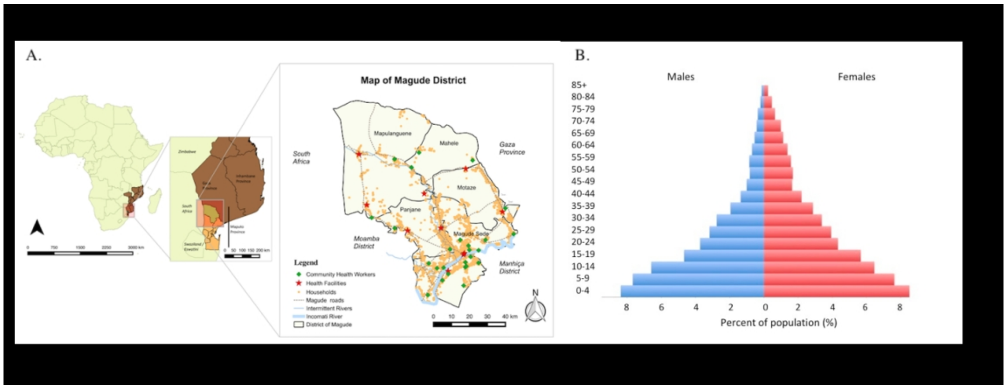

Fig1

119x45mm (300 x 300 DPI)

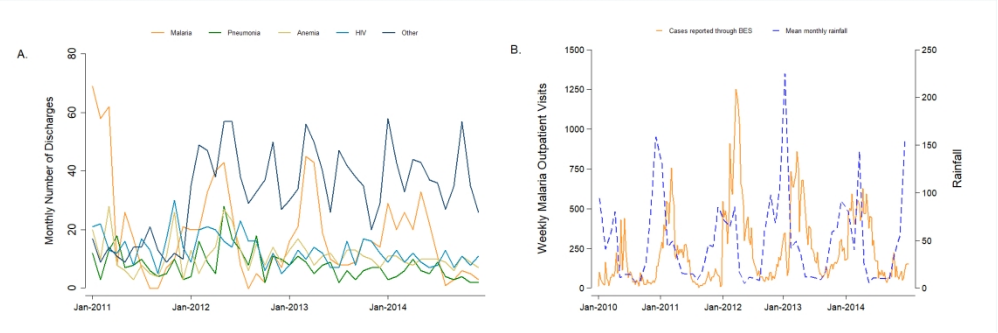

Fig2

169x56mm (300 x 300 DPI)

Supplementary Tables

S.Table 1: Selected demographic and socioeconomic characteristics of Magude's population (2015).

Variable / by sex	Males		Females	
	N	Col %	N	Col %
**Age groups (years)**	21775		26673	
< 1	826	3.8	898	3.4
1-4	3391	15.6	3475	13.0
5-14	7345	33.8	7262	27.2
15-64	9334	42.9	13205	49.5
>=65	864	4.0	1824	6.8
**Education Level (>= 6 years old)**	16712		21461	
Illiterate	7925	47.4	11136	51.9
5-9 grade	6221	37.2	7616	35.5
10-12 grade	1595	9.5	1825	8.5
University	43	0.3	20	0.1
Missing Information	928	5.6	864	4.0
**Occupation (> 18 years old)**	5243		5532	
Farmer or Fisherman	1371	26.1	3909	70.7
Salesperson	466	8.9	609	11.0
Construction work occupations	1159	22.1	39	0.7
Coal maker / Lumberjack	603	11.5	173	3.1
Guards/Police/Military	539	10.3	45	0.8
Health professional	85	1.6	133	2.4
Teacher	263	5.0	180	3.3
Miner	106	2.0	9	0.2
Other	651	12.4	435	7.9
**Marital Status (> 14 years old)**	10157		14986	
Single (never married)	6168	60.7	7772	51.9
Married or de-facto union	3690	36.3	4702	31.4
In a polygamic relationship	202	2.0	-	-
Divorced	7	0.1	13	0.1
Separated	138	1.4	541	3.6
Widow	154	1.5	1958	13.1

For peer review only

S. Table 2: Modified household poverty index (PI) based on the following deprivation indicators: 1) lack of electricity, 2) lack or sharing of an improved sanitation facility; 3) lack of access to improved drinking water source, or source only available more than 30-minute walk, round trip; 4) dirt, sand or dung household floors; 5) dung, wood or charcoal used for cooking fuel; and 6) households that do not own a car or truck AND do not own more than one of the following: radio, TV, telephone, bike, motorbike, or refrigerator.

Deprivations	Magude	Motaze	Panjane	Mahele	Mapulanguene	District
0-2	32.1	9.8	1	0.9	13	25.2
3-4	49.4	58.3	55.1	61	70.8	52.3
5-6	18.5	31.9	43.9	38.1	16.2	22.4
Median	3	4	4	4	3	4
Mean	3.1	3.9	4.2	4.2	3.5	3.4

For peer review only

S.Table 3: Number of individuals by age group who reported spending one night outside of Magude the day before the census visit was performed (2015).

		Men		Women		All	
Age group	Previous night spent outside of Magude	N	(%)	N	(%)	N	(%)*
0 - 14	Yes	592	5.1	580	5.0	1172	43,0*
	No	10874	94.0	10970	94.3		
	NA±	96	0.8	85	0.7		
15 – 29	Yes	324	6.3	362	5.6	686	25.1*
	No	4774	92.7	6022	93.3		
	NA±	52	1.0	70	1.1		
30 – 44	Yes	298	11.3	197	5.3	495	18.1*
	No	2323	87.8	3497	93.9		
	NA±	24	0.9	30	0.8		
45 – 64	Yes	133	8.6	149	4.9	282	10.3*
	No	1393	90.5	2839	93.8		
	NA±	13	0.8	39	1.3		
> 65	Yes	36	4.2	57	3.1	93	3.4*
	No	818	94.7	1744	95.6		
	NA±	10	1.2	23	1.3		
All ages	Yes	1383	6.4	1345	5.0	2728	5.6
	No	20182	92.7	25072	94,0	45254	93.5
	NA±	195	0.9	247	1.0	442	0.9

* Percentage of those who reported travelling (2,728)

± Missing Information

S.Table 4: Household-level health and malaria prevention indicators in Magude district (2015).

Administrative Post	Magude-Sede	Motaze	Panjane	Mahele	Mapulanguene

Household size	4 [3-6]		4 [3-7]		4 [2-6]		4 [2-6]		3 [2-5.5]	
Members per net Median [IQR]	2.0 [1.4-4.0]		2.0 [1.3-3.0]		2.0 [1.6-5.5]		2.0 [1.5-4.0]		2.0 [1.3-4.0]	
Universal ITN coverage *	52.7%		59.9%		44.1%		52.7%		52.1%	
IRS in past 12 months	N	%	N	%	N	%	N	%	N	%
Yes	3925	49.0	1162	79.0	258	41.1	230	61.0	147	30.7
No	3627	45.3	293	19.9	352	56.1	133	35.3	314	65.6
Unknown	459	5.7	16	1.1	17	2.7	14	3.7	18	3.8

** One net for every 2 members of the households

Supplementary Figures

S.Figure 1: Household Assets in Magude district in 2015.

Percentage of households in Magude district with at least one item per asset, and median and interquartile range of the assets for which the number of items available was higher than 1.

S.Figure 2: Mobility patterns of Magude's population in 2015.

Main destinations per age group among those who reported travelling outside of Magude in 2015.

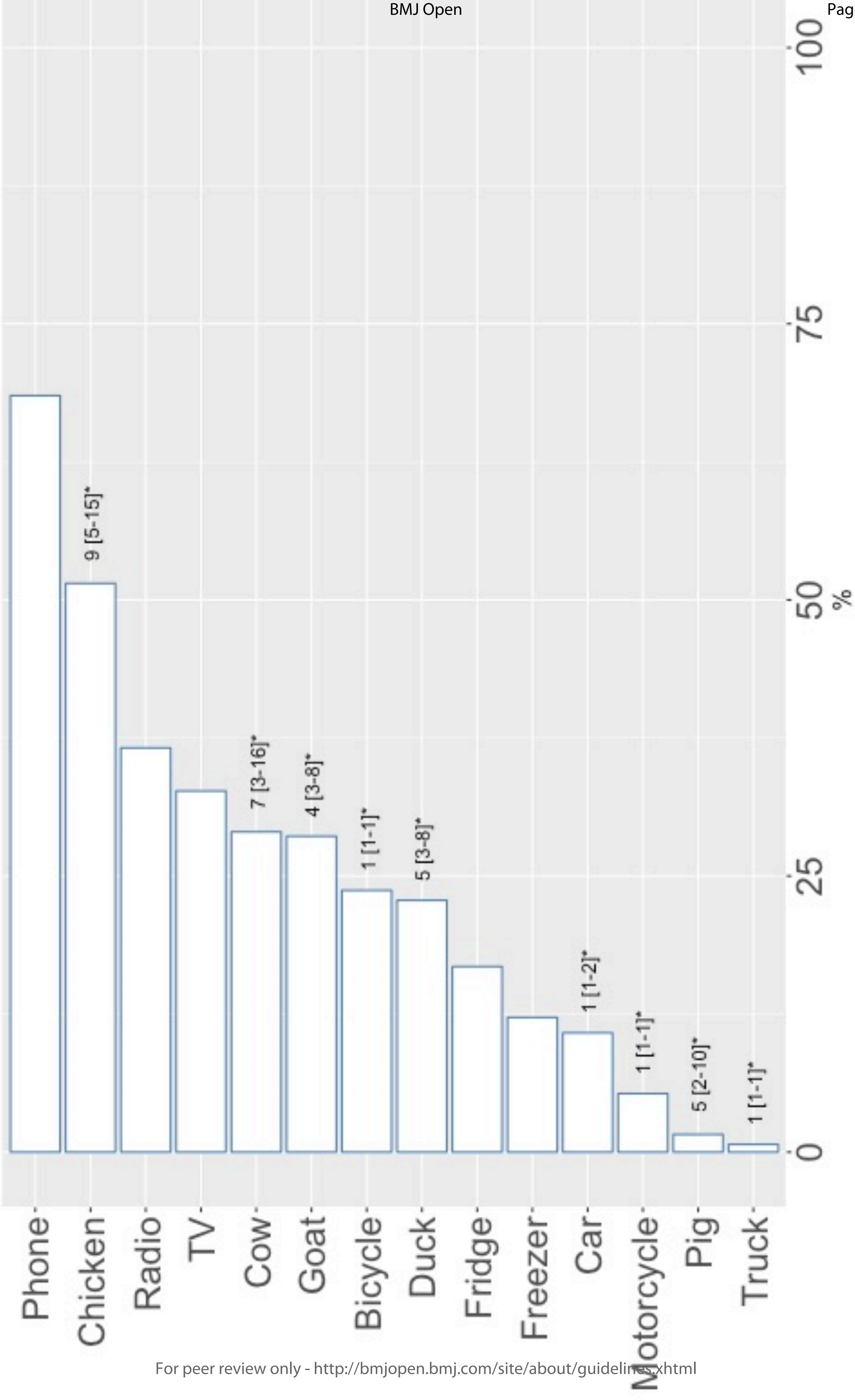
Phone
Chicken
Radio
TV
Cow
Goat
Bicycle
Duck
Fridge
Freezer
Car
Motorcycle
Pig
Truck
9 [5-15]*
7 [3-16]*
4 [3-8]*
1 [1-1]*
5 [3-8]*
1 [1-2]*
1 [1-1]*
5 [2-10]*
1 [1-1]*
0
25
50
75
100
%

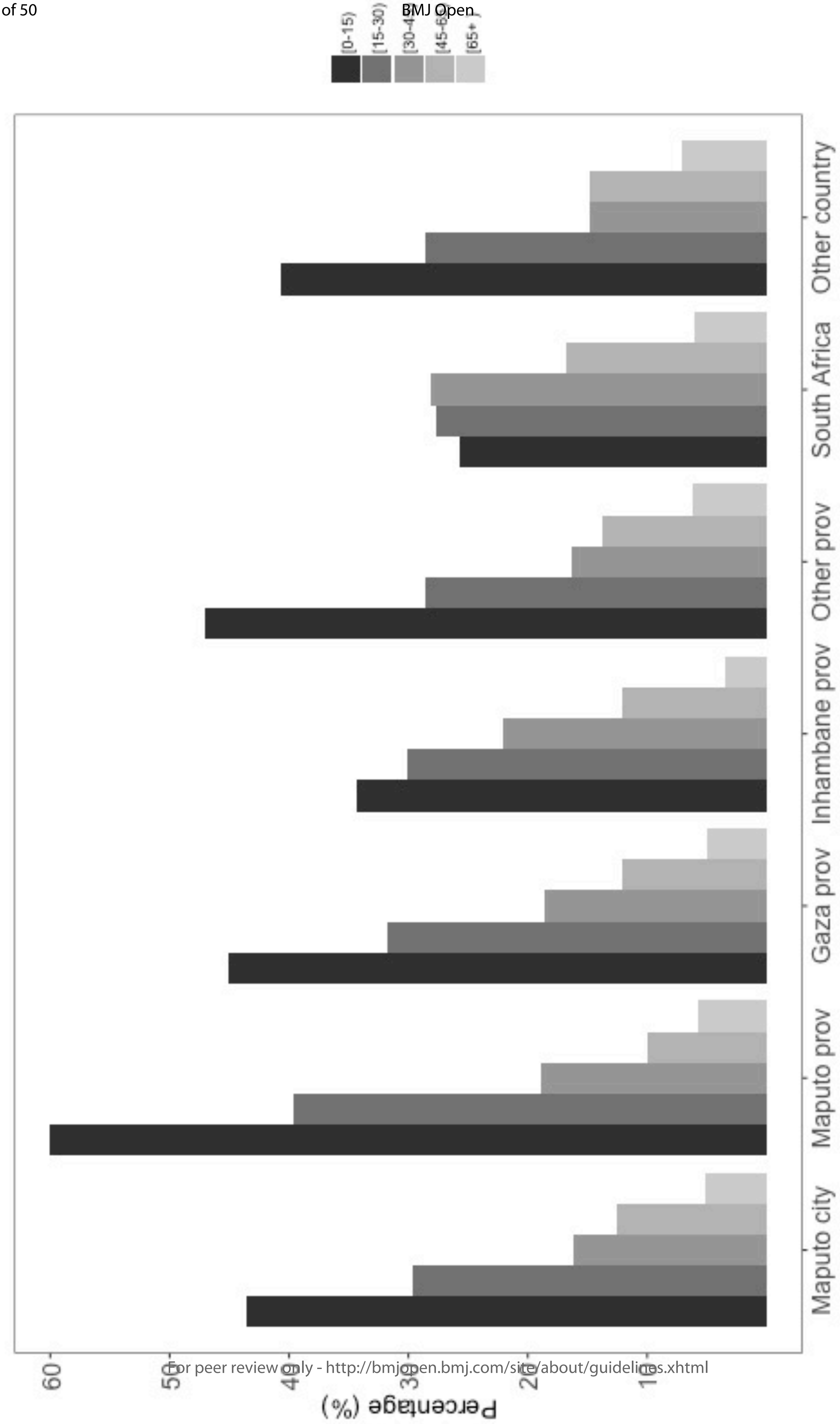
[0-15)
[15-30)
Other country
South Africa
Other prov
Inhambane prov
Gaza prov
Maputo prov
Maputo city
Percentage (%)
10
20
30
40
50
60

STROBE Statement—Checklist of items that should be included in reports of ***cross-sectional studies***

	Item No	Recommendation	Check
**Title and abstract**	1	(*a*) Indicate the study's design with a commonly used term in the title or the abstract	Yes Page 1
		(*b*) Provide in the abstract an informative and balanced summary of what was done and what was found	Yes Page 3
**Introduction**			
Background/rationale	2	Explain the scientific background and rationale for the investigation being reported	Yes Page 6
Objectives	3	State specific objectives, including any prespecified hypotheses	Yes Page 6
**Methods**			
Study design	4	Present key elements of study design early in the paper	Yes Page 7-8
Setting	5	Describe the setting, locations, and relevant dates, including periods of recruitment, exposure, follow-up, and data collection	Yes Page 7
Participants	6	(*a*) Give the eligibility criteria, and the sources and methods of selection of participants	Yes Page 8
Variables	7	Clearly define all outcomes, exposures, predictors, potential confounders, and effect modifiers. Give diagnostic criteria, if applicable	NA Pages 8-9
Data sources/ measurement	8*	For each variable of interest, give sources of data and details of methods of assessment (measurement). Describe comparability of assessment methods if there is more than one group	Yes Pages 8-9
Bias	9	Describe any efforts to address potential sources of bias	Yes Page 4
Study size	10	Explain how the study size was arrived at	NA
Quantitative variables	11	Explain how quantitative variables were handled in the analyses. If applicable, describe which groupings were chosen and why	Yes Page 8
Statistical methods	12	(*a*) Describe all statistical methods, including those used to control for confounding	Yes Page 8
		(*b*) Describe any methods used to examine subgroups and interactions	NA
		(*c*) Explain how missing data were addressed	Page 8
		(*d*) If applicable, describe analytical methods taking account of sampling strategy	Yes Page 8
		(*e*) Describe any sensitivity analyses	NA
**Results**			
Participants	13*	(a) Report numbers of individuals at each stage of study—eg numbers potentially eligible, examined for eligibility, confirmed eligible, included in the study, completing follow-up, and analysed	Yes Page 9
		(b) Give reasons for non-participation at each stage	Yes Page 9
		(c) Consider use of a flow diagram	No
Descriptive data	14*	(a) Give characteristics of study participants (eg demographic, clinical, social) and information on exposures and potential confounders	Yes Page 9-10
		(b) Indicate number of participants with missing data for each variable	Yes

For peer review only - http://bmjopen.bmj.com/site/about/guidelines.xhtml

		of interest	Table 2, S.Table1, S.Table3
Outcome data	15*	Report numbers of outcome events or summary measures	Yes Pages 9-12
Main results	16	(*a*) Give unadjusted estimates and, if applicable, confounder-adjusted estimates and their precision (eg, 95% confidence interval). Make clear which confounders were adjusted for and why they were included	NA
		(*b*) Report category boundaries when continuous variables were categorized	Yes Table2, S.Table1-3
		(*c*) If relevant, consider translating estimates of relative risk into absolute risk for a meaningful time period	NA
Other analyses	17	Report other analyses done—eg analyses of subgroups and interactions, and sensitivity analyses	NA
**Discussion**			
Key results	18	Summarise key results with reference to study objectives	Yes Pages 12-15
Limitations	19	Discuss limitations of the study, taking into account sources of potential bias or imprecision. Discuss both direction and magnitude of any potential bias	Yes Pages 12-15
Interpretation	20	Give a cautious overall interpretation of results considering objectives, limitations, multiplicity of analyses, results from similar studies, and other relevant evidence	Yes Pages 12-15
Generalisability	21	Discuss the generalisability (external validity) of the study results	Yes Pages 12-15
**Other information**			
Funding	22	Give the source of funding and the role of the funders for the present study and, if applicable, for the original study on which the present article is based	Yes Page 17

*Give information separately for exposed and unexposed groups.

Note: An Explanation and Elaboration article discusses each checklist item and gives methodological background and published examples of transparent reporting. The STROBE checklist is best used in conjunction with this article (freely available on the Web sites of PLoS Medicine at http://www.plosmedicine.org/, Annals of Internal Medicine at http://www.annals.org/, and Epidemiology at http://www.epidem.com/). Information on the STROBE Initiative is available at www.strobe-statement.org.

For peer review only

BMJ Open

Demographic and health community-based surveys to inform a malaria elimination project in Magude district, Southern Mozambique

Journal:	*BMJ Open*
Manuscript ID	bmjopen-2019-033985.R2
Article Type:	Original research
Date Submitted by the Author:	12-Mar-2020
Complete List of Authors:	Galatas, Beatriz; Instituto de Salud Global Barcelona, Malaria Elimination Initiative; Centro de Investigacao em Saude de Manhica, ISGLobal<br>Nhacolo, Ariel; Centro de Investigacao em Saude de Manhica<br>Marti, Helena; Instituto de Salud Global Barcelona, Malaria Elimination Initiative<br>Munguambe, Humberto; Centro de Investigacao em Saude de Manhica<br>Jamise, Edgar; Centro de Investigaçao em Saúde de Manhiça<br>Guinovart, Caterina; Barcelona Institute for Global Health<br>Cirera, Laia; Instituto de Salud Global Barcelona, Malaria Elimination Initiative<br>Amone, Felimone; Centro de Investigacao em Saude de Manhica<br>Macete, Eusebio; Centro de Investigação em Saúde de Manhiça<br>Bassat, Quique; BARCELONA INSTITUTE FOR GLOBAL HEALTH<br>Rabinovich, Regina; Instituto de Salud Global Barcelona, Malaria Elimination Initiative<br>Alonso, Pedro; WHO, GMP<br>Aide, Pedro; Manhiça Health Research Centre<br>Saute, Francisco; Centro de Investigação em Saúde de Manhiça<br>Sacoor, Charfudin; Centro de Investigacao em Saude de Manhica, Department of Demography
**Primary Subject Heading**:	Global health
Secondary Subject Heading:	Epidemiology, Global health, Public health
Keywords:	Public health < INFECTIOUS DISEASES, Demography < TROPICAL MEDICINE, Magude project, Malaria elimination, Population and health

SCHOLARONE™
Manuscripts

I, the Submitting Author has the right to grant and does grant on behalf of all authors of the Work (as defined in the below author licence), an exclusive licence and/or a non-exclusive licence for contributions from authors who are: i) UK Crown employees; ii) where BMJ has agreed a CC-BY licence shall apply, and/or iii) in accordance with the terms applicable for US Federal Government officers or employees acting as part of their official duties; on a worldwide, perpetual, irrevocable, royalty-free basis to BMJ Publishing Group Ltd ("BMJ") its licensees and where the relevant Journal is co-owned by BMJ to the co-owners of the Journal, to publish the Work in this journal and any other BMJ products and to exploit all rights, as set out in our licence.

The Submitting Author accepts and understands that any supply made under these terms is made by BMJ to the Submitting Author unless you are acting as an employee on behalf of your employer or a postgraduate student of an affiliated institution which is paying any applicable article publishing charge ("APC") for Open Access articles. Where the Submitting Author wishes to make the Work available on an Open Access basis (and intends to pay the relevant APC), the terms of reuse of such Open Access shall be governed by a Creative Commons licence – details of these licences and which Creative Commons licence will apply to this Work are set out in our licence referred to above.

Other than as permitted in any relevant BMJ Author's Self Archiving Policies, I confirm this Work has not been accepted for publication elsewhere, is not being considered for publication elsewhere and does not duplicate material already published. I confirm all authors consent to publication of this Work and authorise the granting of this licence.

For peer review only

Demographic and health community-based surveys to inform a malaria elimination project in Magude district, Southern Mozambique

Authors

Beatriz Galatas[1, 2*¶], Ariel Nhacolo¶[1], Helena Martí-Soler[1,2], Humberto Munguambe[1], Edgar Jamise[1], Caterina Guinovart[2], Laia Cirera[1,2], Felimone Amone[1], Eusébio Macete[1,3], Quique Bassat[1,2,5,6], N. Regina Rabinovich[2,7], Pedro L. Alonso[1,2.], Pedro Aide[1,4], Francisco Saute[1], Charfudin Sacoor[1]

Affiliations

1. Centro de Investigação em Saúde da Manhiça (CISM), Manhiça, Mozambique;
2. Barcelona Institute for Global Health (ISGlobal), Hospital Clínic-Universitat de Barcelona, Barcelona, Spain.
3. National Directorate of Health, Ministry of Health, Mozambique
4. National Institute of Health, Ministry of Health, Maputo, Mozambique
5. ICREA, Pg. Lluís Companys 23, 08010 Barcelona, Spain.
6. Pediatric Infectious Diseases Unit, Pediatrics Department, Hospital Sant Joan de Déu (University of Barcelona), Barcelona, Spain
7. Harvard T.H. Chan School of Public Health, Boston, MA, USA.

*Corresponding Author

E-mail: Beatriz.galatas@isglobal.org

¶ These authors contributed equally to this work

Emails of Authors

Beatriz.galatas@isglobal.org

Ariel.nhacolo@manhica.net

Helena.marti@isglobal.org

Humberto.munguambe@manhica.net

Laia.cirera@isglobal.org

Felimone.amone@manhica.net

Eusebio.macete@manhica.net

quique.bassat@isglobal.org

For peer review only

For peer review only - http://bmjopen.bmj.com/site/about/guidelines.xhtml

regina.rabinovich@isglobal.org

Alonsop@who.int

Pedro.aide@manhica.net

Francisco.saute@manhica.net

Charfudin.sacoor@manhica.net

Key words

Magude district, census, demographic surveillance site, population, health, malaria control tools, malaria elimination

Word count

5467

For peer review only

For peer review only - http://bmjopen.bmj.com/site/about/guidelines.xhtml

Abstract

(Word count: 302)

Objectives: A Demographic and Health Platform (DHP) was established in Magude in 2015, prior to the deployment of a project aiming to evaluate the feasibility of malaria elimination in southern Mozambique, named the Magude project. This platform aimed to inform the design, implementation and evaluation of the Magude project, through the identification of households, and population; and the collection of demographic, health and malaria information.

Setting: Magude is a rural district of Southern Mozambique which borders South Africa. It has nine peripheral health facilities and one referral health center with an inpatient ward.

Intervention: A baseline census enumerated and geolocated all the households, and their resident and non-resident members, collecting demographic and socio-economic information, and data on the coverage and usage of malaria control tools. Inpatient and outpatient data during the five years (2010-2014) before the survey were obtained from the district health authorities. The demographic platform was updated in 2016.

Results: The baseline census conducted in 2015 reported 48,448 (92.1%) residents and 4,133 (7.9%) non-residents, and 10,965 households. Magude's population is predominantly young, half of the population has no formal education and the main economic activities are agriculture and fishing. Houses are mainly built with traditional non-durable materials and have poor sanitation facilities. Between 2010 and 2014, malaria was the most common cause of all-age inpatient discharges (representing 20-40% of all discharges), followed by HIV (12-22%), and anemia (12-15%). In early 2015, all-age bed-net usage was between 21.8% and27.1% and the reported coverage of indoor residual spraying varied across the district between 30.7% and 79%.

Conclusion: This study revealed that Magude has limited socio-economic conditions, poor access to health care services and low coverage of malaria vector control interventions. Thus, Magude represented an area where it is most pressing to demonstrate the feasibility of malaria elimination.

For peer review only - http://bmjopen.bmj.com/site/about/guidelines.xhtml

Strengths and limitations of this study

- **Strengths:**
  - A Demographic and Health Platform (DHP) was established in Magude in 2015 to enumerate and geolocate all the households, and their resident and non-resident members in order to update the demographic information of the district which was last collected in 2007 during a national census.
  - A second census round was conducted one year later acknowledging that detailed population data collected at individual level at one point in time may miss individuals or households that can be captured after a later update.
  - The census rounds were planned in close collaboration with the community leaders and district authorities, and counted with intensified training of fieldworkers, and a strong component of field and data supervision by experienced demographers.
- **Limitations:**
  - Data collected through the DHP could have been affected by inaccuracies during data collection or entry, or by recall or desirability bias of census participants.
  - Inpatient and malaria outpatient information was limited by the quality and accuracy of the data at the time it was collected, by disease-specific interventions or changes in diagnostics, referral or reporting practices.

For peer review only - http://bmjopen.bmj.com/site/about/guidelines.xhtml

Background

Mozambique is one of the countries with the highest malaria burden in the world [1]. Malaria prevalence among children of 6 to 59 months of age is heterogeneous within the country ranging from high transmission intensity in the North (>50%) to less than 3% in the South, as reported in the latest malaria survey conducted in 2015 [2]. Lower prevalence in the Southern region may be related to the great progress against malaria in the past decades, partly as a result of the regional initiatives aiming for malaria elimination in the area. In line with the vision of a malaria free world established by the World Health Organization in its Global Technical Strategy for 2016-2030 [3], the National Malaria Control Program of Mozambique (NMCP) decided to redefine its strategic objectives in order to include the implementation of malaria elimination activities in the South. In this context, a malaria elimination project named the Magude Project was designed and evaluated in Magude district, Maputo province, by the Manhiça Health Research Center (CISM) and the Barcelona Institute of Global Health (ISGlobal), to assist the NMCP in adopting a malaria elimination strategy based on local evidence [4].

Prior to the initiation of the Magude project in 2015, there was limited and outdated information with regards to the number of individuals living in Magude, as well as to their demographic and socio-economic characteristics [5]. Thus, detailed information from the whole district was deemed crucial to inform the elimination strategies that had been planned for the following years in the district. The process of filling in this knowledge gap also aimed to identify and contact key leaders at provincial, district, and at community level, to inform and engage them in the activities prior to their deployment. In this context, a Demographic and Health Platform adapted from the Health and Demographic Surveillance System (HDSS) method was established in the district of Magude in February of 2015 with the objective of providing reliable and updated demographic data to inform the project. This platform allowed to plan the activities and to provide a sampling frame to measure indicators in the community such as malaria prevalence, and to estimate the coverage of indoor residual spraying (IRS), coverage and usage of long-lasting insecticide treated nets (LLIN) and mass drug administration (MDA) campaigns. The DHP's permanent identification numbers were used to track individual's participation in each intervention of the Magude project longitudinally, thus allowing to identify and quantify potential challenges to the project, such as reasons for non-participation, or probable sources of imported infections. Data were also used to accurately measure prevalence at the community stratified by age groups and place of residence. Overall, these findings were

For peer review only - http://bmjopen.bmj.com/site/about/guidelines.xhtml

crucial to the design of the Magude project, and offered robust evidence to guide malaria elimination strategies in Southern Mozambique.

This article presents the demographic, socio-economic and health characteristics of the population of Magude, as well as the coverage of malaria control interventions estimated through the baseline census conducted between February and June of 2015. It also presents the burden of disease in Magude during the five years (2010-2014) before the DHP, using the inpatient and outpatient data obtained from the district health authorities. A summary of the demographic profile of Magude after updating the census between August and September of 2016 is also provided

Methods

Study area

The district of Magude is located in the North-Western part of Maputo Province and borders with the districts of Massingir, Chókwe and Bilene, from Gaza Province on the North and North-East; with the districts of Manhiça and Moamba, of Maputo Province in the East and South; and with the South African National Kruger Park, in the West (Figure 1A).

Magude was selected as an appropriate demonstration area for the malaria elimination project as it was expected to pose the types of challenges that the NMCP would face when implementing a malaria elimination campaign in the south of the country. This is, there were more than 13,000 malaria cases reported in the district in 2014, with the majority of cases observed between January and May, suggesting that the epidemiology of malaria in the district was representative of most endemic areas in the country with the typical seasonal pattern coinciding with the rainy season [6,7]. Additionally, the socio-economic and infrastructural limitations reported for Magude made it sufficiently representative of a rural district of Mozambique, while still at reach of CISM's facilities (located in Manhiça district), which facilitated the logistics, supervision and quality control processes. Finally, the population of Magude had had very limited exposure to research projects or targeted innovative malaria interventions prior to 2015, having only received the programmatic IRS, LLINs and child immunization campaigns conducted by the government. This allowed facing the challenges of working in an unexposed population that was not biased by previous activities.

For peer review only

Study design

A Demographic and Health Platform (DHP) was established in Magude in February 2015 by CISM, which was adapted from the health and demographic surveillance system (HDSS) previously established in Manhiça district also by CISM [8]. A baseline census was conducted at that time (February to June) to identify and enumerate all neighborhoods, households and resident and non-resident individuals living in these households. Geographic positioning system (GPS) coordinates were also captured for every household.All individuals were assigned a permanent and unique identification number, linked to the household number where they were first enumerated. A second census round was conducted between August and September of 2016, to review and update all information collected during the baseline census and record live births, deaths, and migrations that occurred between the two censuses. New households and new members were enumerated, and their information was collected to update the baseline databases.

This DHP defined a household as a structure or set of constructions where an individual or group of individuals live and share domestic activities and costs (such as eating and sleeping) and recognize one of them as their superior or chief, regardless of their kinship ties. Individuals were defined as residents if they had lived and slept in a household within the study area for a period of three months or more or intended to do so. Non-resident members of a household were defined as those who left the district or who had never lived in the study area for a period of three or more months, but who kept their roles in the households as head of household, husband, or other important breadwinners, and paid regular visits to their households in Magude. These individuals leave their households for specific reasons such work, studies, or imprisonment; and would otherwise reside in the referred household. This category excluded offspring or other members who had left the household to live in their own households outside the study area, irrespective of whether they kept visiting the reference household or not.

Data collection procedures

Baseline and updated data from the census rounds were collected through standardized questionnaires using Open Data Kit (https://opendatakit.org/) installed in Android tablets. Data were sent to a secure server at CISM using Wi-Fi. Information collected on households' socio-economic characteristics included building materials used for the main house, source of water and electricity, and household assets and livestock. At the individual level, the information collected included the name, sex, date of birth, relation to the head of household, education, and occupation of all residents and non-residents of Magude.

For peer review only - http://bmjopen.bmj.com/site/about/guidelines.xhtml

Information was also collected on malaria prevention tools, including the possession and use of LLINs, and whether the house had received IRS in the 12 months preceding the census. Individuals were also asked about history of fever during the preceding 30 days, and whether they had sought care at a health facility, community health worker, traditional healer, or none. The specific health facility where the individual sought care was also specified to delineate health facility catchment areas (HFCA) according to the community. Finally, information was collected on the migration and mobility patterns of all participants, to better characterize the mobility profile of the district, and offer potential information on the sources of new infections if transmission was eventually significantly reduced.

Administrative and geographical information was obtained directly from the district authorities and from the most recent district profile of Magude published in 2014 by the central government authority [7] and district statistics performed by the National Institute of Statistics [9,10]. We retrospectively collected monthly data on inpatient discharges from the referral health center of Magude Sede, as well as weekly outpatient malaria cases reported through the weekly epidemiological bulletin (Boletim Epidemiológico Semanal or "BES" in Portuguese) for the period of 2010-2014. The BES did not distinguish between presumed and confirmed cases, the diagnostic used (rapid diagnostic test or microscopy) or the location of detection (health facility or in the community).

Patient and Public Involvement

This study counted with the support and involvement of the community of Magude in all of its stages. During its design and planning, meetings were held with the district authorities and community leaders in order to inform them of the purpose of the DHP under the scope of the Magude project, as well as to landscape the number of villages in Magude that had to be covered by the DHP. The study team worked closely with village chiefs when performing the household visits, which ensured that all district households were covered by the DHP. Reports were submitted to the administrative authorities to support vector control activities, among others. Preliminary findings were also communicated in meetings held with the community prior to the deployment of MDAs, where the findings obtained from the study – particularly focusing on malaria – where discussed in detail [11].

Data analysis

Data management and descriptive analyses were performed using R Software [12]. The percentage distribution of categories within any given variable were calculated considering the observations with missing values in the denominator. No inferential statistics (p-values or 95% confidence intervals) were

For peer review only - http://bmjopen.bmj.com/site/about/guidelines.xhtml

calculated as the DHP covers the entire district, and therefore offers direct population parameters. The relationship between outpatient malaria cases and rainfall was evaluated using linear regressions for different rainfall lags. Spatial visualization of the data collected on the field and obtained from web-based open source administrative data bases from DIVA-GIS [13] were performed using QGIS Software [14].

Household's socio-economic status was measured using a modified version of Oxford's Poverty and Human Development Initiative Multidimensional Poverty Index (MPI) [15], following the same methodology used by the INDEPTH group to estimate the poverty index of various HDSS sites throughout Africa [16]. In summary, this method categorizes households as "deprived" or "not deprived" based on six deprivation indicators: 1) lack of electricity, 2) lack or sharing of an improved sanitation facility; 3) lack of access to improved drinking water source, or source only available more than 30-minute walk, round trip; 4) sand floors in main houses; 5) dung, wood or charcoal used for cooking fuel; and 6) households that do not own a car nor truck and do not own more than one of the following: radio, TV, telephone, bike, motorbike, or refrigerator. A deprivation indicator is generated as lowest depravation (0-2), moderate depravation (3-4), or highest depravation (5-6) [16].

Results

The geography of Magude district

The district of Magude has an area of 6,961 km^2 and is divided in five administrative posts, namely: Magude-sede, Motaze, Panjane, Mahele and Mapulanguene (Figure 1A). The vegetation of Magude is dominated by open forests and savannahs hosting animals such as impalas, warthogs, lions, buffalos and elephants. There is one permanent river (Incomati), which flows through the south-western region of the district and constitutes the main source of water in the area, and three intermittent rivers dependent on rainfall, called Massitonto, Uanétse and Mazimuchopes [7].

Demographic and socio-economic characteristics

The baseline census of 2015 registered 10,965 households and 52,802 individuals, of whom 48,448 (91.8%) were residents and 4,133 (7.8%) were non-residents. The district had a population density of 6.9 inhabitants per km^2, which varies by administrative post. The majority of the population (73%) lives in the Magude-Sede administrative post (the capital of the district); 14.1% in Motaze, 5.9% in Panjane, 3.7% in

For peer review only - http://bmjopen.bmj.com/site/about/guidelines.xhtml

Mapulanguene, and 3.4% in Mahele (Table 1). The age and sex structure of the population is dominated by children and young people, and a sharp reduction of adults, particularly among males (Figure 1B).

The census update conducted between August and October of 2016 registered a population of 61,868 individuals. Of the population censed in 2015, 42,792 (81%) were found during the update round, 6,099 (11.5%) were reported to still be in Magude by a family member although field workers did not find them in other households and therefore were not able to confirm and complete the registration of migration. There were an additional 3,244 (6.1%) individuals censed in 2015 who were not found in 2016 and for whom there were no informants, but were still considered to be in Magude for this analysis. This update also recorded the death of 670 individuals censed in 2015, 1,687 live births and 721 immigrations since the baseline census. An additional 7,325 individuals who were missed in 2015 were censed during this update (Table 1).

The baseline census indicated that 62% of males and 63.1% of females above the age of 14 reported being married or in de-facto union; and 3% of married men practice polygamy. The prevalence of lack of formal education among those aged 6 and older in Magude is 51.9% among females and 47.4% among males. Approximately 37.2% of individuals reached 5th to 9th grade, and 9.5% have completed between 10th and 12th grade (Sup.Table 1). In 2015 Magude had 31 primary schools offering 1st to 5th grades; 33 offering 1st-7th grades; one secondary school offering 8th-12th grades; and one private higher-education training center. According to the national census performed in 2007, Xichangana is the main local language of the district and is the mother tongue of 92% of the population of Magude. However, 51% of the population also reports being able to speak Portuguese (the official language in Mozambique), which is the mother tongue of only 3.2% in the district [7].

Occupations are unevenly distributed between males and females older than 18 years of age. The majority of the population (26.1% of males and 70.7% of females) relies on subsistence agriculture, fishing or work as cane cutters in the sugar plantations within Magude, or in Xinavane, in the nearby district of Manhiça. Other occupations include being a salesperson (8.9% of men and 11% of women), doing construction work (22.1% of men, 0.7% of women), or making coal (11.5% of men and 3.1% of women). A small proportion of the population works as security guards, or work in the public sector, particularly as health professionals, teachers or in the army (Sup.Table 1).

For peer review only - http://bmjopen.bmj.com/site/about/guidelines.xhtml

Men represent 54.6% of heads of households in Magude. Only 0.4% of household heads are younger than 18 years of age and 5.6% are between the ages of 18-24, while 74.7% are between 25-64 years old, and 19.2% are older than 65 years of age (Table 2). Forty-three percent of heads of household are single, 35.4% are married or in de-facto union, 16.8% are widows and 4.3% separated. Fifty-nine percent of heads of households reported having no formal education, while 28.2% reported completing up to 5-9th grade, 8.7% up to 10th to 12th grade, and 0.4% reported having a university degree (Table 2).

Living conditions in Magude's households

Settlements in Magude are made of individual household compounds that are built in proximity to each other in the central areas of each administrative post, and more spread out in the rest of the district. The median household size is 5, although 1,171 households (10.7%) have only one resident, 40% of whom are older than 65 years old.

The majority of households are traditional round-shaped or rectangular-shaped huts constructed using cane (32.5%), cement (26%), mud bricks (21.6%), or reeds covered by adobe (15.6%) (Table 2). More than half of the households have traditional latrines (53.5%), 10.6% have improved latrines, while 33.8% of households do not have any form of sanitation facility. Flush toilets can only be found in 2.2% of the households. The primary lighting sources used in the households are paraffin (49.6%), electricity (30.8%), candles (9.9%) and solar panels (4.6%). The majority of households cook using wood logs (59.3%). Water is mainly obtained from pumps (34.3%) or piped water (20.5%), although a quarter of the population collects water directly from the river (24.9%) or from open wells near the river (10.5%) (Table 2).

With regards to household assets, 68.5% of households own at least one mobile phone, 36.6% own a radio, 32.7% have a television (TV), while 16.8% and 12.2% of households have a fridge and a freezer, respectively. Almost a quarter of households reported having bicycles (23.7%), while 10.8% reported owning cars, 5.3% motorbikes, and 0.7% trucks (Sup.Figure 1).

Households in Magude had a median number of 4 depravations. Twenty-five percent of the households were in the lowest depravation category, with 0-2 deprivation indicators; while 55.3% fell in the 3-4 depravation group and 22.4% in the 5-6 depravation group. This distribution was unevenly observed among the different administrative posts. Magude Sede had the highest proportion of households in the lowest depravation group (32%), while Mapulanguene had the highest proportion of households in the moderate

For peer review only - http://bmjopen.bmj.com/site/about/guidelines.xhtml

depravation category (70.8%). Panjane and Mahele were the administrative posts with the highest proportion of highly deprived households (Sup. Table 2).

Mobility patterns

The baseline census showed that 5% of residents reported having spent the night before the census outside of Magude (6.4% of males and 5% of females). Of these, 43% were younger than 14 years old, 25.1% were 15-29, 18.1% were 30-44, 10.3% were 45-64 and 3.4% were older than 65 years old (Sup.Table 3).

Almost half of the population (43% of males and 46.9% of females) did not report frequently travelling outside of Magude during the baseline census. Among the 54.4% who did, the places most frequently visited included Maputo City (55.3% of males, 58.3% of females), South Africa (23.1% of males and 16.8% of females), Gaza Province (12.2% of males and 15.9% of females), Inhambane Province (4.3% males and 2.6% females), and other Northern provinces within Mozambique (5.2% of males and 6.3% of females). Younger individuals (less than 15 years old) reported travelling the most to all provinces within the country, followed by those between the ages of 15-30; while 15 to 45-year-olds are more likely to travel to South Africa. Individuals older than 45 reported travelling more to provinces northern of Maputo province, as well as abroad (Sup.Figure 2).

Health and malaria information

The district has 10 functional health facilities– one with an inpatient and maternity ward (Type I) and nine HFs only with maternity wards (Type II). Eight of the ten HFs were active in 2015, and 2 more Type II HFs were added in 2016 and 2017. The referral HF, located in the Magude-Sede administrative post, has 57 beds and three active medical doctors (0.06 doctors per 1,000 inhabitants). The other 9 Type II health facilities have 37 beds in total. The whole district has only three medical doctors, 15 general nurses and 19 maternal and child health nurses (Figure 1A). Only 3 HFs have access to piped water, 4 have access to public network electricity, and 5 rely only on solar panels. All health facilities are located on a local main road to facilitate access, and the median Euclidian distance from households to the nearest health facility is 2.7 km (interquartile range [IQR] 1.4–7.9 km), although households are as close as 15 m or as far as 38.8 km. There is only one ambulance in Magude, which is used for transferring patients within the district, but also to the hospitals in Xinavane, Manhiça, and Maputo.

For peer review only - http://bmjopen.bmj.com/site/about/guidelines.xhtml

The health system in Mozambique includes Community Health Workers (or APEs, from its acronym in Portuguese) who are trained by the Ministry of Health to offer primary health services in areas with poor access to HFs. They provide diagnosis and treatment for malaria, diarrhea, and pneumonia, and to refer patients with signs of sickness requiring higher medical attention [17]. In Magude there are 27 APEs distributed throughout the district (Figure 1A). The average distance between households and the nearest APE is approximately 6.3 km (median of 4.4 km, IQR 2.8–5.4 km).

All HFs and APEs in Magude are equipped to diagnose malaria through Rapid Diagnostic Tests (RDTs) and light microscopy is only available in the Magude Sede Type I HF. They also offer treatment to all positive cases with Artemether-Lumefantrine, the first line treatment in Mozambique. Intermittent Preventive Treatment in pregnancy (IPTp) using Sulfadoxine-pyrimethamine is also offered in all HFs of the district. In May of 2014 the NMCP conducted an LLIN universal distribution campaign, with 35,432 bed nets distributed in Magude district. This was followed by a focal IRS campaign between October and December of 2014 using the insecticides deltamethrine and DDT, which was only deployed in the Motaze administrative post, where the malaria burden was highest.

Malaria has traditionally been the first cause of disease in the district, responsible for approximately 53% of all consultations reported in 2003 [7]. According to the inpatient discharge data available from January of 2010 to December of 2014, between 20-40% of all-age discharges in the inpatient department were due to malaria, followed by HIV (12-22%), anemia (12-15%), pneumonia (6-12%), and diarrhea (3-8%), varying by month and year (Fig 2A). The weekly number of malaria cases reported through the BES in Magude between 2010 and 2014 follows a seasonal pattern with a peak between December and May and a reduction of cases during the dry season (Fig 2B). According to BES data, there were 293 cases per 1000 population reported during the transmission year of July 2011 to June 2012, 247 per 1000 between July 2012 and June 2013, 252 cases per 1000 between July 2013 and June 2014, and 110 cases per 1,000 between July 2014 and June 2015. [7]. There is a fairly linear association between the rainfall in the preceding 2 months, and the number of malaria cases in Magude (linear regression coefficient=13.6, Rho=0.64, p-value<0.001).

The prevalence of history of fever during the 30 days prior to the baseline census was 12.1% in children under the age of 5, 7.7% among 5-14 year-olds, and 11.6% in individuals older than 15 years. Almost all individuals reported going to a health facility as their primary source of health care (>99%), while a small proportion mentioned seeking care first from traditional healers or community health workers. The

For peer review only - http://bmjopen.bmj.com/site/about/guidelines.xhtml

proportion of individuals who reported sleeping under a bed net the night before was 27.1% among children under the age of 5, 21.8% in 5-14 year-olds, and 27% among those > 15 years of age (Table 3). The universal bed-net coverage (i.e. one net for every two individuals of a household) in 2015 was 52.7% in the administrative post of Magude-Sede, 59.9% in Motaze, 44.1% in Panjane, 52.7% in Mahele and 52.1% in Mapulanguene. Finally, the reported coverage of IRS during the previous 12 months was 49% in Magude-Sede, 79% in Motaze, 41.1% in Panjane, 61% in Mahele, and 30.7% in Mapulanguene (Table 2 and Sup.Table 4).

Discussion

Overall, the Demographic and Health Platform established in Magude offered detailed insight on the baseline socio-demographic profile of Magude district prior to the Magude project. It revealed that the number of residents in Magude in 2015 (52,804 individuals and 10,965 households) was similar to the number reported by the 2007 national census conducted by the National Institute of Statistics (INE) (53,229 individuals and 11,408 households) [9] but did not coincide with the projected population for Magude in 2015 estimated by the INE to be 62,000 [10], which is not surprising acknowledging the limitations of population projections. The census update conducted in 2016 identified individuals who had been missed by the baseline census, and recorded the births, deaths, and in-migrations since baseline. As a result, the population of Magude in 2016 assuming that those censed in 2015 who were not found in 2016 were still living in the district, was 61,868 individuals, which is similar to the projection by INE for this year (62,924).

The usefulness of a demographic and health surveillance system in demographic and bio-medical research in African countries has been previously described [18]. Having a precise source of population data is crucial for the correct estimation of the risk of disease and coverage of health indicators in a population, as well as for planning purposes of public health interventions [19]. This platform has shown that detailed population data collected at individual level at one point in time still misses a number of individuals, who can be captured after a later update. However, all the census rounds faced the challenge of finding individuals at home, or informants of unavailable individuals, to identify whether a member lives in the area despite not being found during a specific census round. To identify or collect information about absent individuals, field supervisors attempt to arrange interviews with them at their places of work, or interview their family members at their households during weekends. The same phenomenon is expected to take place when

For peer review only - http://bmjopen.bmj.com/site/about/guidelines.xhtml

implementing malaria interventions in the community (such as rounds of IRS or of mass drug administrations), which generally challenges the estimation of intervention coverage.

Household-level data indicates that the households in Magude are typical of a rural area with suboptimal conditions regarding access to water, electricity, and sanitation. The majority of houses were relatively homogeneous, built with non-durable materials, such as cane and adobe, and did not have access to clean water, conditions indicative of a low socio-economic status [20]. The age and sex composition of Magude's population is typical of rural Mozambique and sub-Saharan Africa, composed primarily by children and young individuals and a decreasing proportion of adults as age increases [21]. Another important aspect of the structure of the population of Magude is the lower sex ratio (number of males in relation to that of females) among young and middle-aged adults, which has been reported, in the neighbouring district of Manhiça, to be related to male labour migration to Maputo city or South Africa and higher male mortality [21].

A large proportion of the heads of households and of the overall population of Magude reported not having received a formal education, a major risk factor for health outcomes. Additionally, most individuals reported having occupations related to agriculture or fishing, which are usually carried out early in the morning until around noon. These aspects should be taken into consideration when planning mobilization campaigns and community-based interventions, as visits might have to be conducted at certain times of day and/or venues outside the households to be able to find these individuals.

Magude is also subject to significant mobility and migration, as more than half of the population reported travelling frequently outside of the district. The destinations most reported by travelers living in Magude were Maputo and South Africa, followed by Gaza and Inhambane provinces. This information is useful to evaluate the risk of malaria importation from other areas due to travel or migration [22]. Maputo city and South Africa are areas with lower malaria burden than Magude [23]; however, the district of Manhiça surrounding Magude, the provinces of Gaza, Inhambane and the rest of Mozambique have higher malaria prevalence estimates than Magude district [2], and could be a source of imported infections [24]. These places are commonly visited by all age groups, whereas the lower-endemic areas are mainly visited by <30 year-olds. This age pattern of migration is typical of the rural areas of Southern Mozambique, where migration rates are higher among individuals aged 20 to 45 years and their children, and decrease with increasing age when individuals establish as permanent residents in an area.

For peer review only - http://bmjopen.bmj.com/site/about/guidelines.xhtml

The population of Magude has access to ten health facilities, one of them with an inpatient ward with 57 beds. Thus there are 11.8 beds per 10,000 population, a ratio that is higher than the national level (7 beds per 10,000 in Mozambique) [25]. While most respondents indicated seeking health care primarily from formal health facilities, the majority of the population is scattered throughout the district as far as 38 kms away from a HF and it is difficult to access health facilities or APEs due to limited roads and lack of transportation. This has implications for the delivery of public health interventions, especially for those that rely on the passive detection of cases to control transmission in the community.

Data between 2010 and 2014 from the outpatient and inpatient department of the Magude Sede health center indicated that malaria was the main cause of hospitalization and later discharge with a clear seasonal pattern. The population of Magude is also burdened by HIV, anemia, pneumonia, and diarrhea, similar to its neighboring districts of Manhiça and Chokwé [26–33]. Inpatient information was retrospectively collected, and thus, its quality and accuracy depends on the data at the time at which it was collected. It may also be biased by disease-specific interventions or changes in diagnostics, referral protocols of severe cases to other districts, or reporting practices. Additionally, cases reported by BES are difficult to interpret given their lack of disaggregation in presumed or confirmed, diagnostic tool used or place of detection of the case. Thus, they are subject to changes in RDT use and stock-outs, in care seeking behavior to HFs or APEs, or reporting inaccuracies. In fact, cases reported by the APEs are usually excluded by those who report in BES, and neither the total number of fevers nor the number of individuals tested are reported. Thus, a stronger surveillance system than BES was thought to be required, and consequently established in Magude in 2015, to fully capture the clinical malaria profile of a district aiming to eliminate malaria [4,34].

The age-specific self-reported history of fever in the past 30 days (7.7%-12.1%) were slightly lower than the average percentage of fevers reported in the previous 2 weeks in Maputo Province, which was 15% according to the most recent malaria indicator survey [2]. Approximately half of the households in Magude reported owning one bed net for every two individuals (universal coverage). However, only a fourth of the population reported sleeping under a bed net the previous night. These findings mirror the estimates reported for Maputo Province in 2011 by the Demographic Health Survey, which found a reported bed net usage rate of 27% in Maputo Province [35]. This information is indicative of an area where bed net owners do not necessarily use the net as a preventive tool against malaria during the rainy season, despite this being a prevalent disease in the district. Other demographic and socio-economic risk factors that have been

For peer review only - http://bmjopen.bmj.com/site/about/guidelines.xhtml

associated with LLIN use, such as households with more children, household heads with no formal education, or households far away from the health facilities, might explain the low bed net usage in Magude [36–38].

The reported IRS coverage in the area of Motaze was higher, which corresponds with the focal IRS campaign conducted by the NMCP that took place only there in September of 2014. The coverage reported in other areas was probably the consequence of recall bias, as a district-wide IRS campaign took place in 2013. Overall, the coverage reported for LLINs and IRS throughout the district was below the World Health Organization recommended coverage of >80% [39], leaving a significant proportion of the population unprotected by any of the standard preventative measures. The identification of gaps in such vector control coverages called for a strong community engagement campaign focused on the use of the bed nets and participation in the yearly rounds of IRS.

Conclusion

Through the establishment of a Demographic and Health Platform in Magude in 2015, which was updated in 2016, and a baseline assessment of the relevant health indicators, it was possible to fully characterize a district where malaria elimination interventions were to be deployed and evaluated. Magude represents a typical rural district of Mozambique characterized by limited social and economic infrastructures, which had to be considered for the design and operationalization of the community-based interventions. This study also revealed a low education level among a large proportion of the population and identified agriculture as the main economic activity in the district. These socio-demographic characteristics suggest that a strong community engagement would be necessary for the implementation of a malaria elimination project. Half of the population of Magude reported travelling outside of the district to areas of high and low malaria transmission intensity, showing that malaria importation will likely be a source of continuous transmission even if interruption of local transmission is achieved. Also, the poor access to health care services, as well as to core malaria vector control interventions such as IRS and bed nets distributed by the NMCP, indicated that these aspects needed to be integrated within the malaria elimination program planned for the district, in order to maximize the impact of the interventions aiming to interrupt transmission. Overall, this district represents the reality of the majority of malaria endemic areas in Sub-Saharan Africa, where elimination is most needed, and where it is most pressing to demonstrate the feasibility of elimination strategies.

For peer review only - http://bmjopen.bmj.com/site/about/guidelines.xhtml

For peer review only

For peer review only - http://bmjopen.bmj.com/site/about/guidelines.xhtml

Abbreviations

APEs – Agentes Polivalentes Elementais (Community Health Workers)

BES – Boletim Epidemiológico Semanal (Weekly Epidemiological Bulletin)

CISM – Manhiça Health Research Center

DHP – Demographic and Health Platform

GPS – Global Positioning System

HDSS – Health and Demographic Surveillance System

HF – Health Facility

HIV – Human Immunodeficiency virus

INE – Instituto Nacional de Estatística (National Institute of Statistics)

IPTp – Intermittent Preventive Treatment in pregnancy

IRS – indoor residual spraying

ISGlobal – Barcelona Institute of Global Health

LLINs – Long-Lasting Insecticide treated Nets

MDAs – Mass Drug Administration

NMCP - National Malaria Control Program of Mozambique

RDTs – Rapid Diagnostic Tests

TV – Television

Declarations

Acknowledgements

We would like to thank the community of Magude for participating in this study, and the team of field workers who have collected the data presented here. We would also like to acknowledge the Administrative and District Health Authorities of Magude for their collaboration and for providing some of the information also included in this article. We thank everyone who supported this study directly or indirectly through fieldwork, or analysis support.

Ethics approval and consent to participate

The Demographic and Health Platform protocol, consent forms and questionnaires were approved by the CISM internal ethics committee and written consent from the health authorities was also sought prior to its implementation. Meetings were held with community leaders and with general members of the

For peer review only

community of Magude to inform them about the DHP operations and the malaria elimination project as a whole. A written informed consent was obtained from all household heads to record household-level information, as well as from all individuals providing individual information. Informed consents for children under the age of 18 years were sought from their parents or primary caretakers. The collection of district health surveillance data was conducted under the protocol that aimed to evaluate the impact of the *Magude project* on malaria transmission, which was approved by CISM's internal ethical committee, the Ethics Committee of the Hospital Clínic of Barcelona, and the Ministry of Health National Bioethics Committee of Mozambique (IRB00002657).

Competing Interests

The authors declare that they have no competing interests

Funding

Funding was provided by the Bill and Melinda Gates Foundation and the Fundación "la Caixa" Partnership for the Elimination of Malaria in Southern Mozambique (OPP1115265). Q. B. is an ICREA (Institut Catal. de la Recerca i Estudis Avan.ats; Catalan Government) Research Professor. ISGlobal is a member of the CERCA Programme, Generalitat de Catalunya.

Author's Contributions

BG: participated in the study design and fieldwork, supported in the data cleaning and data analysis process, and wrote the draft of this article

AN: participated in the study design, in study analyses, in the interpretation of results and writing of this article

HMS: cleaned and analyzed the data, and contributed to the writing of this article

HM: led the implementation of field activities and data collection process, and participated in the interpretation of results

EJ: participated in the study design and implementation of field activities and data collection.

CG: participated in the interpretation of results and writing of this article

LC: participated in the study design, interpretation of results and writing of this article

FA: designed the data collection tools, supported the implementation of field activities and data cleaning.

EM: participated in the study design, and interpretation of results

QB: participated in the study design, interpretation of results and writing of this article

NRR: participated in the interpretation of results and writing of this article

For peer review only

For peer review only - http://bmjopen.bmj.com/site/about/guidelines.xhtml

PLA: participated in the study design, interpretation of results and writing of this article

PA: participated in the study design, field implementation interpretation of results and writing of this article

FS: participated in the study design, supervised field activities, interpretation of results and writing of this article

CS: conceived the study, participated in interpretation of results and writing of this article

Availability of data

The datasets collected as part of the Demographic Health Platform and analysed during the current study are available from the corresponding author upon written request.

The data presented in figures 2a and 2b belongs to the Ministry of Health of Mozambique and is considered as third-party data which can be accessed by contacting the following individuals appointed by the Ministry of health:

- To gain access to Inpatient Department data collected by the Ministry of health please contact Dr Baltazar Candrinho, Head of NMCP Mozambique, phone number:+258828665730 or e-mail: candrinhobaltazar@gmail.com
- To gain access to the data collected through the Boletim Epidemiologico Semanal (BES) please contact Dr Lorna Gujral, Department of Epidemiology/MOH/Mozambique, phone number: +25882 3250800 or e-mail: lornita15.lgg@gmail.com

For peer review only

For peer review only - http://bmjopen.bmj.com/site/about/guidelines.xhtml

References

1. World Health Organization. World malaria report 2018. :210.

2. Ministério da Saúde (MISAU), Instituto Nacional de Estatística (INE), ICF Internacional. Inquérito de Indicadores de Imunização, Malária e HIV/SIDA em Moçambique 2015. Maputo. Moçambique. Rockville, Maryland, EUA: INS, INE e ICF International; 2015.

3. World Health Organization, others. A framework for malaria elimination. 2017 [cited 2017 Mar 28]; Available from: http://apps.who.int/iris/handle/10665/254761

4. Aide P, Candrinho B, Galatas B, Munguambe K, Guinovart C, Luis F, et al. Setting the scene and generating evidence for malaria elimination in Southern Mozambique. Malar J. 2019;18:190.

5. Instituto Nacional de Estatistica. Relatório Final do Inquérito ao Orçamento Familiar- IOF 2014/15 [Internet]. 2015. Available from: http://www.ine.gov.mz/operacoes-estatisticas/inqueritos/inquerito-sobre-orcamento-familiar

6. Maharaj R, Moonasar D, Baltazar C, Kunene S, Morris N. Sustaining control: lessons from the Lubombo spatial development initiative in southern Africa. Malar J [Internet]. 2016 [cited 2016 Oct 17];15. Available from: http://malariajournal.biomedcentral.com/articles/10.1186/s12936-016-1453-9

7. Ministerio de Administração Estatal. Perfil do distrito de Magude, Provincia de Maputo (2014). Ministerio de Administração Estatal; 2014.

8. Alonso P, Saúte F, Aponte J, Gómez-Olivé F, Nhacolo A, Thompson R, et al. Manhiça demographic surveillance system, Mozambique. Popul Health Surviv INDEPTH Sites. 2002;1:295–308.

9. Instituto Nacional de Estatística. Estatísticas do Distrito de Magude. 2010.

10. Instituto Nacional de Estatistica. População Projectada por distritos, Maputo Província 2007_2040.xls — Instituto Nacional de Estatistica [Internet]. [cited 2018 May 29]. Available from: http://www.ine.gov.mz/estatisticas/estatisticas-demograficas-e-indicadores-sociais/projeccoes-da-populacao/populacao-projectada-por-distritos-maputo-provincia-2007_2040.xls/view

11. Portugaliza HP, Galatas B, Nhantumbo H, Djive H, Murato I, Saúte F, et al. Examining community perceptions of malaria to inform elimination efforts in Southern Mozambique: a qualitative study. Malar J [Internet]. 2019 [cited 2019 Aug 21];18. Available from: https://malariajournal.biomedcentral.com/articles/10.1186/s12936-019-2867-y

For peer review only - http://bmjopen.bmj.com/site/about/guidelines.xhtml

12. R, Foundation for Statistical Computing. R: A language and environment for statistical computing [Internet]. Vienna, Austria; Available from: http://www.R-project.org/

13. Hijmans R, Cruz M, Rojas E, Guarino L. DIVA-GIS, version 1.4. A geographic information system for the management and analysis of genetic resources data. 2001;

14. QGIS Development Team (2017) [Internet]. Open Source Geospatial Foundation Project; Available from: http://qgis.osgeo.org

15. Alkire S, Santos ME. Acute Multidimensional Poverty: A New Index for Developing Countries. SSRN Electron J [Internet]. 2010 [cited 2020 Feb 8]; Available from: http://www.ssrn.com/abstract=1815243

16. Coates MM, Kamanda M, Kintu A, Arikpo I, Chauque A, Mengesha MM, et al. A comparison of all-cause and cause-specific mortality by household socioeconomic status across seven INDEPTH network health and demographic surveillance systems in sub-Saharan Africa. Glob Health Action. 2019;12:1608013.

17. Ndima SD, Sidat M, Give C, Ormel H, Kok MC, Taegtmeyer M. Supervision of community health workers in Mozambique: a qualitative study of factors influencing motivation and programme implementation. Hum Resour Health. 2015;13:63.

18. Bocquier P, Sankoh O, Byass P. Are health and demographic surveillance system estimates sufficiently generalisable? Glob Health Action. 2017;10:1356621.

19. Tatem AJ, Campiz N, Gething PW, Snow RW, Linard C. The effects of spatial population dataset choice on estimates of population at risk of disease. Popul Health Metr. 2011;9:4.

20. Pons-Duran C, González R, Quintó L, Munguambe K, Tallada J, Naniche D, et al. Association between HIV infection and socio-economic status: evidence from a semirural area of southern Mozambique. Trop Med Int Health. 2016;21:1513–21.

21. Nhacolo AQ, Nhalungo DA, Sacoor CN, Aponte JJ, Thompson R, Alonso P. Levels and trends of demographic indices in southern rural Mozambique: evidence from demographic surveillance in Manhiça district. BMC Public Health [Internet]. 2006 [cited 2017 Jun 6];6. Available from: http://bmcpublichealth.biomedcentral.com/articles/10.1186/1471-2458-6-291

22. Ruktanonchai NW, Bhavnani D, Sorichetta A, Bengtsson L, Carter KH, Córdoba RC, et al. Census-derived migration data as a tool for informing malaria elimination policy. Malar J. 2016;15:273.

23. Moonasar D, Maharaj R, Kunene S, Candrinho B, Saute F, Ntshalintshali N, et al. Towards malaria elimination in the MOSASWA (Mozambique, South Africa and Swaziland) region. Malar J [Internet]. 2016

For peer review only

For peer review only - http://bmjopen.bmj.com/site/about/guidelines.xhtml

[cited 2016 Aug 25];15. Available from: http://malariajournal.biomedcentral.com/articles/10.1186/s12936-016-1470-8

24. Ruktanonchai NW, Smith DL, De Leenheer P. Parasite sources and sinks in a patched Ross–Macdonald malaria model with human and mosquito movement: Implications for control. Math Biosci. 2016;279:90–101.

25. World Health Organization. GHO | By country | Mozambique - statistics summary (2002 - present) [Internet]. WHO. [cited 2018 May 29]. Available from: http://apps.who.int/gho/data/node.country.country-MOZ?lang=en

26. González R, Augusto OJ, Munguambe K, Pierrat C, Pedro EN, Sacoor C, et al. HIV Incidence and Spatial Clustering in a Rural Area of Southern Mozambique. PloS One. 2015;10:e0132053.

27. Feldblum PJ, Enosse S, Dubé K, Arnaldo P, Muluana C, Banze R, et al. HIV prevalence and incidence in a cohort of women at higher risk for HIV acquisition in Chókwè, southern Mozambique. PloS One. 2014;9:e97547.

28. Deus N de, João E, Cuamba A, Cassocera M, Luís L, Acácio S, et al. Epidemiology of Rotavirus Infection in Children from a Rural and Urban Area, in Maputo, Southern Mozambique, before Vaccine Introduction. J Trop Pediatr. 2018;64:141–5.

29. Nhampossa T, Mandomando I, Acacio S, Quintó L, Vubil D, Ruiz J, et al. Diarrheal Disease in Rural Mozambique: Burden, Risk Factors and Etiology of Diarrheal Disease among Children Aged 0-59 Months Seeking Care at Health Facilities. PloS One. 2015;10:e0119824.

30. Aguilar R, Moraleda C, Quintó L, Renom M, Mussacate L, Macete E, et al. Challenges in the diagnosis of iron deficiency in children exposed to high prevalence of infections. PloS One. 2012;7:e50584.

31. Garcia-Basteiro AL, Miranda Ribeiro R, Brew J, Sacoor C, Valencia S, Bulo H, et al. Tuberculosis on the rise in southern Mozambique (1997-2012). Eur Respir J. 2017;49.

32. Lanaspa M, O'Callaghan-Gordo C, Machevo S, Madrid L, Nhamposa T, Acácio S, et al. High prevalence of Pneumocystis jirovecii pneumonia among Mozambican children <5 years of age admitted to hospital with clinical severe pneumonia. Clin Microbiol Infect Off Publ Eur Soc Clin Microbiol Infect Dis. 2015;21:1018.e9-1018.e15.

For peer review only - http://bmjopen.bmj.com/site/about/guidelines.xhtml

33. Roca A, Sigauque B, Quinto L, Morais L, Berenguera A, Corachan M, et al. Estimating the vaccine-preventable burden of hospitalized pneumonia among young Mozambican children. Vaccine. 2010;28:4851–7.

34. Barclay VC, Smith RA, Findeis JL. Surveillance considerations for malaria elimination. Malar J. 2012;11:304.

35. Ministerio da Saude (MISAU), Instituto Nacional de Estatística (INE) e ICF International (ICFI). Moçambique Inquérito Demográfico e de Saúde 2011. Calverton Md USA MISAU INE E ICFI. 2011;

36. Pinchoff J, Hamapumbu H, Kobayashi T, Simubali L, Stevenson JC, Norris DE, et al. Factors Associated with Sustained Use of Long-Lasting Insecticide-Treated Nets Following a Reduction in Malaria Transmission in Southern Zambia. Am J Trop Med Hyg. 2015;93:954–60.

37. García-Basteiro AL, Schwabe C, Aragon C, Baltazar G, Rehman AM, Matias A, et al. Determinants of bed net use in children under five and household bed net ownership on Bioko Island, Equatorial Guinea. Malar J. 2011;10:179.

38. Atieli HE, Zhou G, Afrane Y, Lee M-C, Mwanzo I, Githeko AK, et al. Insecticide-treated net (ITN) ownership, usage, and malaria transmission in the highlands of western Kenya. Parasit Vectors. 2011;4:113.

39. World Health Organization, World Health Organization, Global Malaria Programme. Global technical strategy for malaria, 2016-2030 [Internet]. 2015 [cited 2018 Mar 5]. Available from: http://apps.who.int/iris/bitstream/10665/176712/1/9789241564991_eng.pdf?ua=1

For peer review only - http://bmjopen.bmj.com/site/about/guidelines.xhtml

Tables

Table 1: Summary of Magude Population in 2015 and 2016.

	District Level		Magude Sede		Motaze		Panjane		Mahele		Mapulan-guene	
	N	%	N	%	N	%	N	%	N	%	N	%
**Households 2015**	10965		8011	73.1	1471	13.4	627	5.7	377	3.4	479	4.4
**Households 2016**	11960		8520	71.3	1441	12.1	645	5.4	803	6.7	547	4.6
**Population 2015**	52802		38534	73.0	7421	14.1	3122	5.9	1784	3.4	1934	3.7
Residents	48448	91.8*	35346	73.0	6605	13.6	2930	6.0	1695	3.5	1868	3.9
Non-residents	4133	7.8*	2970	71.9	816	19.7	191	4.6	89	2.2	66	1.6
Unclassified	221	0.4*	218	99.5	0	-	1	0.5	0	-	0	-
**Population 2016**	61868		44203	71.4	8149	13.2	3576	5.8	3499	5.7	2404	3.8
Residents	56943±	92.1*	40623	71.4	7275	12.8	3361	5.9	3329	5.8	2322	4.1
Non-residents	4535 ø	7.3*	3266	72.0	842	18.6	206	4.5	143	3.2	76	1.7
Unclassified	390 §	0.6*	314	80.9	32	8.2	9	2.3	27	7.0	6	1.5
**Demographic Events 2015-16**	3078		2204	71.6	390	12.7	186	6.0	168	5.5	129	4.2
Births	1687	54.8	1203	71.4	220	13.0	92	5.5	91	5.4	80	4.7
Immigrations	721	23.4	521	72.3	60	8.3	51	7.1	60	8.3	29	4.0
Deaths	670	21.8	480	71.6	110	16.4	43	6.4	17	2.5	20	3.0

* Column %

± 56,943 = 48,448 (censed in 2015) – 616 (deaths) + 1,670 (births) + 1,235 (immigrations) + 6,206 (censed in 2016)

ø 4,535 = 4,133– 42 (deaths) + 12 (births) + 64 (immigrations) + 368 (censed in 2016)

§ 390 = 221 – 8 (deaths) + 5 (births) + 21 (immigrations) + 151 (censed in 2016)

For peer review only

For peer review only - http://bmjopen.bmj.com/site/about/guidelines.xhtml

Table 2: Socio-demographic characteristics of heads of households, and household characteristics (2015).

**Characteristics of heads of households***		**N**	**%**
Sex	Males	5051	54.6
	Females	4206	45.4
Age group (years)	< 18	40	0.4
	18 – 24	520	5.6
	25 – 64	6919	74.7
	> = 65	1777	19.2
Education level	No formal education	5460	59.0
	5-7th grade	2611	28.2
	8 -12th grade	807	8.7
	University	37	0.4
	Missing Information	342	3.7
Marital status	Single	4007	43.3
	Married or de-facto union	3275	35.4
	Divorced	16	0.2
	Separated	393	4.3
	Widow	1554	16.8
Residents of Magude		9257	84.4
**Households characteristics**			
Average household size**		5 [3-7]	
Lone-resident households		1171	10.7
Wall material of the main building	Cane	3506	32.5
	Cement	2807	26.0
	Mud bricks	2338	21.6
	Adobe	1703	15.8
	Zinc plates	319	3.0
	Wood	128	1.2
Type of toilet	Traditional latrine	5774	53.5
	Improved latrine	1144	10.6
	WC connected to septic tank	233	2.2
	No latrine	3650	33.8
Main source of lighting energy	Paraffin	5362	49.6
	Electricity	3321	30.8
	Candles	1074	9.9
	Solar panels	502	4.6
	Other	539	5.0
Kitchen Fuel	Wood logs	6401	59.3
	Coal	740	6.9
	Gas	46	0.4
	Electricity	65	0.6
	Paraffin	8	0.1
	Missing Information	3541	32,8
Primary source of drinking water	Water pumps	3700	34.3
	Directly from the river	2691	24.9
	Piped water	2218	20.5
	Open well near the river	1138	10.5
	Other	1054	9.8
**Vector control tools**			
% of households with >1 ITN		8906	81.2

For peer review only - http://bmjopen.bmj.com/site/about/guidelines.xhtml

Universal ITN coverage (one net per two household members)		5690	53.2
IRS in past 12 months	Yes	5722	52.2
	No	4719	43.0
	Unknown	524	4.8

* or subhead (if head does not live in the household)

**Median [Interquartile Range]

Table 3: Individual-level health and malaria prevention indicators in Magude district 2015).

Age group in Years	< 5		5-14		>15	
	N	%	N	%	N	%
Had fever in preceding 30 days						
Yes	1043	12.1	1128	7.7	2931	11.6
No	7492	87.2	13325	91.2	21977	87.1
Unknown	55	0.6	154	1.1	319	1.3
Primary source of health care						
Health Facility	8552	99.6	14548	99.6	25075	99.4
Traditional Healer	13	0.2	27	0.2	61	0.2
Community Health Worker	22	0.3	32	0.2	66	0.3
Slept under a bed net the preceding night						
Yes	2330	27.1	3179	21.8	6799	27.0
No	6260	72.9	11428	78.2	18428	73.0

For peer review only

For peer review only - http://bmjopen.bmj.com/site/about/guidelines.xhtml

Figure Titles and Legends

Fig 1: Map of Magude district (2015).

A) Administrative and permanent river shape files were obtained from DIVA-GIS ((http://www.diva-gis.org/Data), and confirmed with Magude's key informants. GPS positions of households, health facilities and community health workers obtained directly from the field and mapped using QGIS. B) Population pyramid of Magude district (2015) showing the proportion of five—year age and sex groups out of the total population.

Fig 2: Health profile of Magude district prior to the baseline census (2010-2014).

A) Most common diseases leading to all-age hospitalization and later discharge in Magude reported by the District Health Authorities between 2010-2014. B) Weekly number of outpatient malaria cases observed in Magude between 2010 and 2014 reported through the Weekly Epidemiological Bulletin (BES) and monthly rainfall data obtained from the Climate Hazards Group InfraRed Precipitation with Station data (CHIRPS).

For peer review only

For peer review only - http://bmjopen.bmj.com/site/about/guidelines.xhtml

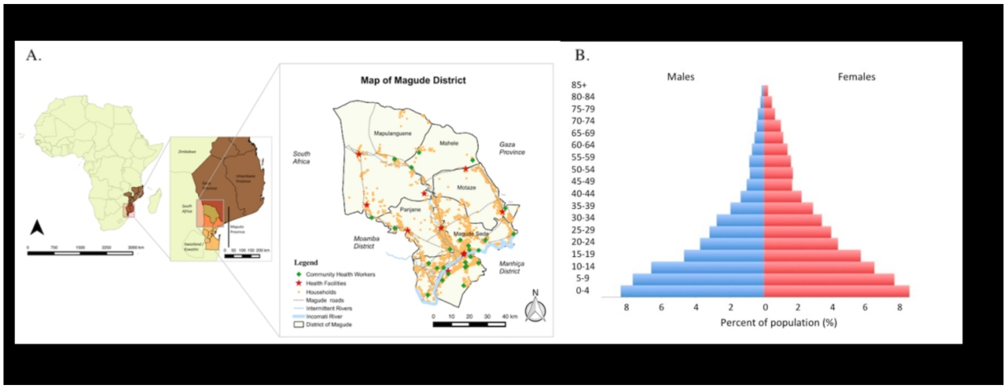

Fig1

119x45mm (300 x 300 DPI)

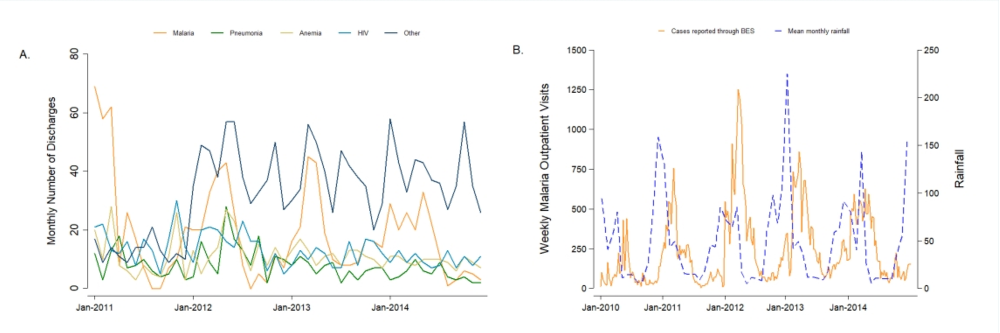

Fig2

169x56mm (300 x 300 DPI)

Supplementary Tables

S.Table 1: Selected demographic and socioeconomic characteristics of Magude's population (2015).

Variable / by sex	Males		Females	
	N	Col %	N	Col %
**Age groups (years)**	21775		26673	
< 1	826	3.8	898	3.4
1-4	3391	15.6	3475	13.0
5-14	7345	33.8	7262	27.2
15-64	9334	42.9	13205	49.5
>=65	864	4.0	1824	6.8
**Education Level (>= 6 years old)**	16712		21461	
Illiterate	7925	47.4	11136	51.9
5-9 grade	6221	37.2	7616	35.5
10-12 grade	1595	9.5	1825	8.5
University	43	0.3	20	0.1
Missing Information	928	5.6	864	4.0
**Occupation (> 18 years old)**	5243		5532	
Farmer or Fisherman	1371	26.1	3909	70.7
Salesperson	466	8.9	609	11.0
Construction work occupations	1159	22.1	39	0.7
Coal maker / Lumberjack	603	11.5	173	3.1
Guards/Police/Military	539	10.3	45	0.8
Health professional	85	1.6	133	2.4
Teacher	263	5.0	180	3.3
Miner	106	2.0	9	0.2
Other	651	12.4	435	7.9
**Marital Status (> 14 years old)**	10157		14986	
Single (never married)	6168	60.7	7772	51.9
Married or de-facto union	3690	36.3	4702	31.4
In a polygamic relationship	202	2.0	-	-
Divorced	7	0.1	13	0.1
Separated	138	1.4	541	3.6
Widow	154	1.5	1958	13.1

For peer review only

S. Table 2: Modified household poverty index (PI) based on the following deprivation indicators: 1) lack of electricity, 2) lack or sharing of an improved sanitation facility; 3) lack of access to improved drinking water source, or source only available more than 30-minute walk, round trip; 4) dirt, sand or dung household floors; 5) dung, wood or charcoal used for cooking fuel; and 6) households that do not own a car or truck AND do not own more than one of the following: radio, TV, telephone, bike, motorbike, or refrigerator.

Deprivations	Magude	Motaze	Panjane	Mahele	Mapulanguene	District
0-2	32.1	9.8	1	0.9	13	25.2
3-4	49.4	58.3	55.1	61	70.8	52.3
5-6	18.5	31.9	43.9	38.1	16.2	22.4
Median	3	4	4	4	3	4
Mean	3.1	3.9	4.2	4.2	3.5	3.4

For peer review only

S.Table 3: Number of individuals by age group who reported spending one night outside of Magude the day before the census visit was performed (2015).

		Men		Women		All	
Age group	Previous night spent outside of Magude	N	(%)	N	(%)	N	(%)*
0 - 14	Yes	592	5.1	580	5.0	1172	43,0 *
	No	10874	94.0	10970	94.3		
	NA±	96	0.8	85	0.7		
15 – 29	Yes	324	6.3	362	5.6	686	25.1 *
	No	4774	92.7	6022	93.3		
	NA±	52	1.0	70	1.1		
30 – 44	Yes	298	11.3	197	5.3	495	18.1 *
	No	2323	87.8	3497	93.9		
	NA±	24	0.9	30	0.8		
45 – 64	Yes	133	8.6	149	4.9	282	10.3 *
	No	1393	90.5	2839	93.8		
	NA±	13	0.8	39	1.3		
> 65	Yes	36	4.2	57	3.1	93	3.4*
	No	818	94.7	1744	95.6		
	NA±	10	1.2	23	1.3		
All ages	Yes	1383	6.4	1345	5.0	2728	5.6
	No	20182	92.7	25072	94,0	45254	93.5
	NA±	195	0.9	247	1.0	442	0.9

* Percentage of those who reported travelling (2,728)

± Missing Information

For peer review only

S.Table 4: Household-level health and malaria prevention indicators in Magude district (2015).

Administrative Post	Magude-Sede		Motaze		Panjane		Mahele		Mapulanguene	
Household size	4 [3-6]		4 [3-7]		4 [2-6]		4 [2-6]		3 [2-5.5]	
Members per net Median [IQR]	2.0 [1.4-4.0]		2.0 [1.3-3.0]		2.0 [1.6-5.5]		2.0 [1.5-4.0]		2.0 [1.3-4.0]	
Universal ITN coverage *	52.7%		59.9%		44.1%		52.7%		52.1%	
IRS in past 12 months	N	%	N	%	N	%	N	%	N	%
Yes	3925	49.0	1162	79.0	258	41.1	230	61.0	147	30.7
No	3627	45.3	293	19.9	352	56.1	133	35.3	314	65.6
Unknown	459	5.7	16	1.1	17	2.7	14	3.7	18	3.8

** One net for every 2 members of the households

For peer review only

Supplementary Figures

S.Figure 1: Household Assets in Magude district in 2015.

Percentage of households in Magude district with at least one item per asset, and median and interquartile range of the assets for which the number of items available was higher than 1.

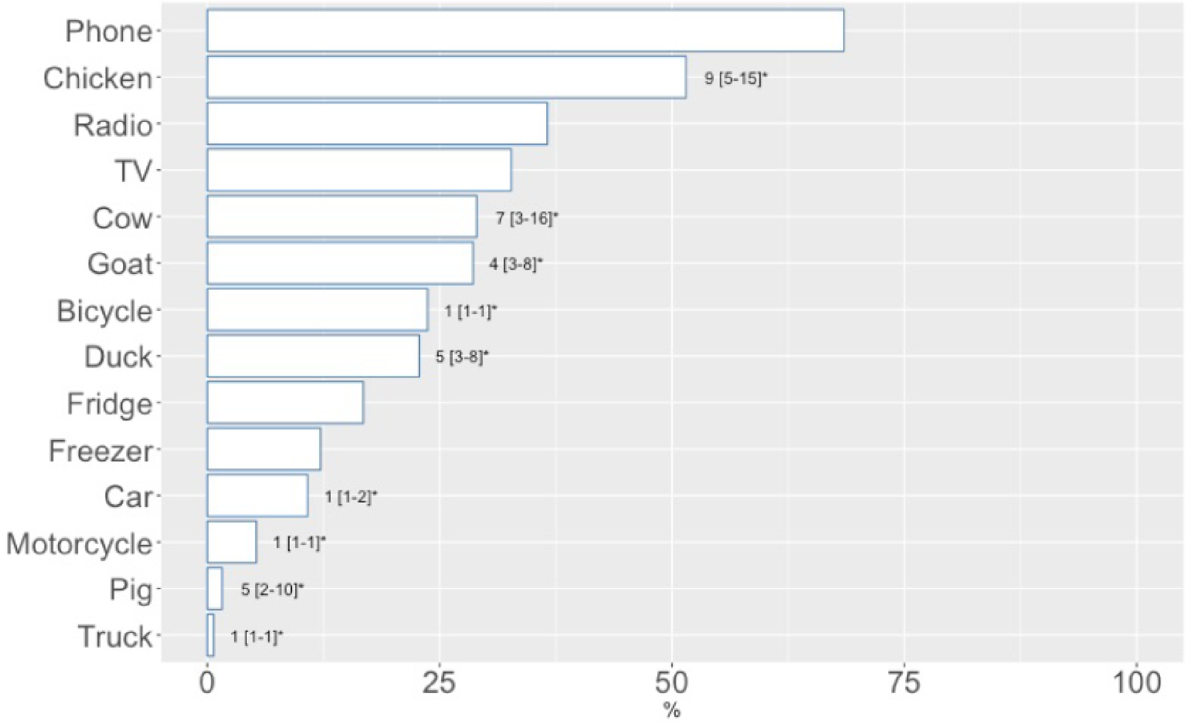

For peer review only - http://bmjopen.bmj.com/site/about/guidelines.xhtml

S.Figure 2: Mobility patterns of Magude's population in 2015.

Main destinations per age group among those who reported travelling outside of Magude in 2015.

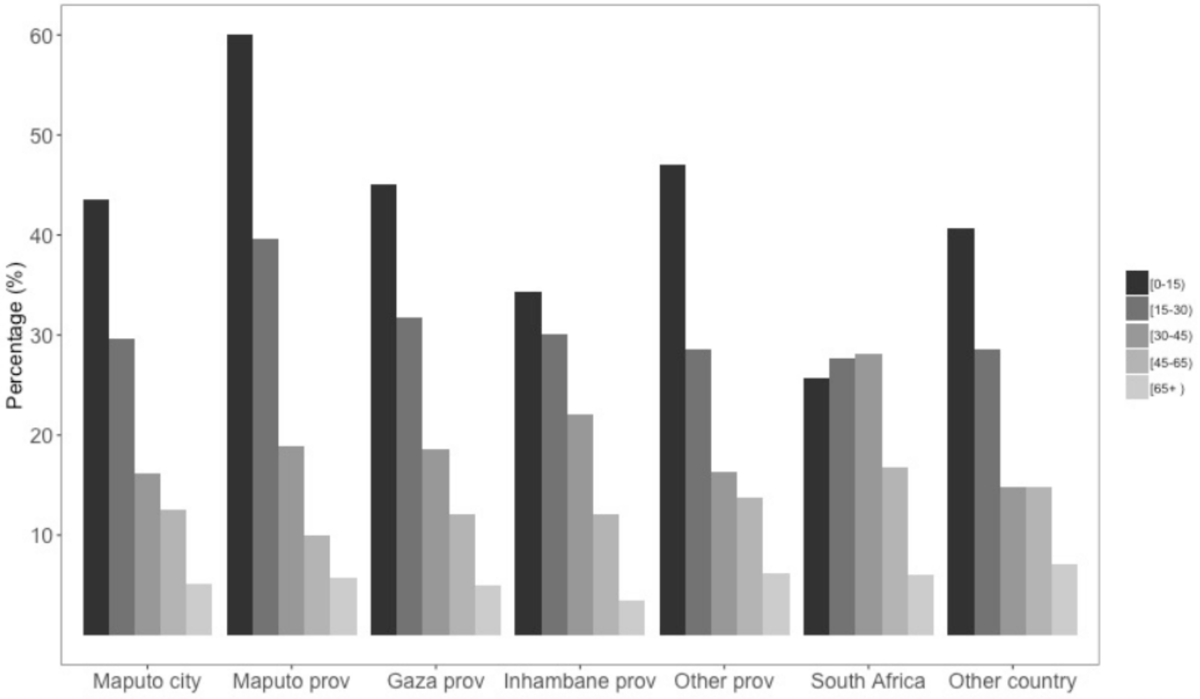

review only

STROBE Statement—Checklist of items that should be included in reports of ***cross-sectional studies***

	Item No	Recommendation	Check
**Title and abstract**	1	(*a*) Indicate the study's design with a commonly used term in the title or the abstract	Yes Page 1
		(*b*) Provide in the abstract an informative and balanced summary of what was done and what was found	Yes Page 3
**Introduction**			
Background/rationale	2	Explain the scientific background and rationale for the investigation being reported	Yes Page 6
Objectives	3	State specific objectives, including any prespecified hypotheses	Yes Page 6
**Methods**			
Study design	4	Present key elements of study design early in the paper	Yes Page 7-8
Setting	5	Describe the setting, locations, and relevant dates, including periods of recruitment, exposure, follow-up, and data collection	Yes Page 7
Participants	6	(*a*) Give the eligibility criteria, and the sources and methods of selection of participants	Yes Page 8
Variables	7	Clearly define all outcomes, exposures, predictors, potential confounders, and effect modifiers. Give diagnostic criteria, if applicable	NA Pages 8-9
Data sources/ measurement	8*	For each variable of interest, give sources of data and details of methods of assessment (measurement). Describe comparability of assessment methods if there is more than one group	Yes Pages 8-9
Bias	9	Describe any efforts to address potential sources of bias	Yes Page 4
Study size	10	Explain how the study size was arrived at	NA
Quantitative variables	11	Explain how quantitative variables were handled in the analyses. If applicable, describe which groupings were chosen and why	Yes Page 8
Statistical methods	12	(*a*) Describe all statistical methods, including those used to control for confounding	Yes Page 8
		(*b*) Describe any methods used to examine subgroups and interactions	NA
		(*c*) Explain how missing data were addressed	Page 8
		(*d*) If applicable, describe analytical methods taking account of sampling strategy	Yes Page 8
		(*e*) Describe any sensitivity analyses	NA
**Results**			
Participants	13*	(a) Report numbers of individuals at each stage of study—eg numbers potentially eligible, examined for eligibility, confirmed eligible, included in the study, completing follow-up, and analysed	Yes Page 9
		(b) Give reasons for non-participation at each stage	Yes Page 9
		(c) Consider use of a flow diagram	No
Descriptive data	14*	(a) Give characteristics of study participants (eg demographic, clinical, social) and information on exposures and potential confounders	Yes Page 9-10
		(b) Indicate number of participants with missing data for each variable	Yes

For peer review only - http://bmjopen.bmj.com/site/about/guidelines.xhtml

		of interest	Table 2, S.Table1, S.Table3
Outcome data	15*	Report numbers of outcome events or summary measures	Yes Pages 9-12
Main results	16	(*a*) Give unadjusted estimates and, if applicable, confounder-adjusted estimates and their precision (eg, 95% confidence interval). Make clear which confounders were adjusted for and why they were included	NA
		(*b*) Report category boundaries when continuous variables were categorized	Yes Table2, S.Table1-3
		(*c*) If relevant, consider translating estimates of relative risk into absolute risk for a meaningful time period	NA
Other analyses	17	Report other analyses done—eg analyses of subgroups and interactions, and sensitivity analyses	NA
**Discussion**			
Key results	18	Summarise key results with reference to study objectives	Yes Pages 12-15
Limitations	19	Discuss limitations of the study, taking into account sources of potential bias or imprecision. Discuss both direction and magnitude of any potential bias	Yes Pages 12-15
Interpretation	20	Give a cautious overall interpretation of results considering objectives, limitations, multiplicity of analyses, results from similar studies, and other relevant evidence	Yes Pages 12-15
Generalisability	21	Discuss the generalisability (external validity) of the study results	Yes Pages 12-15
**Other information**			
Funding	22	Give the source of funding and the role of the funders for the present study and, if applicable, for the original study on which the present article is based	Yes Page 17

*Give information separately for exposed and unexposed groups.

Note: An Explanation and Elaboration article discusses each checklist item and gives methodological background and published examples of transparent reporting. The STROBE checklist is best used in conjunction with this article (freely available on the Web sites of PLoS Medicine at http://www.plosmedicine.org/, Annals of Internal Medicine at http://www.annals.org/, and Epidemiology at http://www.epidem.com/). Information on the STROBE Initiative is available at www.strobe-statement.org.

For peer review only

For peer review only - http://bmjopen.bmj.com/site/about/guidelines.xhtml
